# Supplementary material for: The rise and transformation of Bronze Age pastoralists in the Caucasus
Source: Nature. 2024 Oct 30;635(8040):917–25. doi: 10.1038/s41586-024-08113-5 (PMC11602729; doi:10.1038/s41586-024-08113-5)
Supplement: Supplementary file 1 — Supplementary Notes Sections 1–5 (see contents for details). [file 41586_2024_8113_MOESM1_ESM.pdf]

nature portfolio

https://doi.org/10.1038/s41586-024-08113-5

Supplementary information

The rise and transformation of Bronze Age pastoralists in the Caucasus

In the format provided by the authors and unedited

SUPPLEMENTARY INFORMATION

The rise and transformation of Bronze Age pastoralists in the Caucasus

Ayshin Ghalichi[1,29,*], Sabine Reinhold[2,29,*], Adam B. Rohrlach[1,3], Alexey A. Kalmykov[4], Ainash Childebayeva[1,5], He Yu[1,6], Franziska Aron[1], Lena Semerau[1], Katrin Bastert-Lamprichs[2], Andrey B. Belinskiy[7], Natalia Y. Berezina[8], Yakov B. Berezin[8], Nasreen Broomandkhoshbacht[9], Alexandra P. Buzhilova[8], Vladimir R. Erlikh[10], Lars Fehren-Schmitz[9,11], Irina Gambashidze[12], Anatoliy R. Kantorovich[13], Konstantin B. Kolesnichenko[7], David Lordkipanidze[12], Rabadan G. Magomedov[14], Katharina Malek-Custodis[15], Dirk Mariaschk[2], Vladimir E. Maslov[16], Levon Mkrtchyan[17], Anatoli Nagler[2], Hassan Fazeli Nashli[18], Maria Ochir[19], Yuri Y. Piotrovskiy[20], Mariam Saribekyan[17], Aleksandr G. Sheremetev[21], Thomas Stöllner[22,23], Judith Thomalsky[2], Benik Vardanyan[17,24], Cosimo Posth[1,25,26], Johannes Krause[1,27], Christina Warinner[1,27,28], Svend Hansen[2], Wolfgang Haak[1,*]

This PDF file includes

Supplementary Text

1 Archaeological background of the Caucasus cultures 3
1.1 Archaeological background and chronology 3
1.2 Changing economies of Neolithic, Eneolithic, and Bronze Age Caucasus 7
1.3 Archaeological cultures and genetic groupings 8
2 Archaeological site and sample descriptions 8
2.1. Armenia 10
2.1.1 Lernakert 10
2.2. Georgia 11
2.2.1 Arukhlo 1 11
2.2.2 Arukhlo 2 13
2.2.3 Balitshi-Dzedzvebi 13
2.3 Russian Federation 15
2.3.1 9 Nasypey 15
2.3.2 Aygurskiy 2 16
2.3.3 Bamut 18
2.3.4 Boykoponura 18
2.3.5 Ergeninskiy 1 21
2.3.6 Essentukskiy 1, Kurgan 22
2.3.7 Gatyn-Kale 24
2.3.8 Ginchi 25
2.3.9 Ilinskiy 1 26

2.3.10 Ipatovo 3 27
2.3.11 Kaluzhny 1 28
2.3.12 Kammenomostskoe 29
2.3.13 Khutor Belyy 29
2.3.14 Komsomolec 1 - Marfa 30
2.3.15 Konstantinovskiy 4 33
2.3.16 Konstantinovskiy 6 33
2.3.17 Krasnogvardeyskoe 34
2.3.18 Kurganniy 1 36
2.3.19 Magas-Puteprovod 37
2.3.20 Marinskaya 5 38
2.3.21 Nalchik graveyard 41
2.3.22 Nevinnomyskiy 3 42
2.3.23 Poselenie Kravchenko XII 44
2.3.24 Sengeleevskiy 7 45
2.3.25 Sharakhalsun 3 45
2.3.26 Satanaj grotto (Gubsk rock shelter VII) 46
2.3.27 Shushuk 50 47
2.3.28 Tipki 1 48
2.3.29 Vinogradny 1 (Rostov) 49
2.3.30 Vinsady 4 49
2.3.31 Vinsady 5 50
2.3.32 Zimnyaya Stavka 1 52
2.3.33 Zolotaryevka 1 53
3 Archaeological Site and Sample Information for the non-Caucasus Sites 54
3.1 Iran 54
3.1.1 Bozroodpey – Ghal-e Ben 54
4. Summary information on genetic analyses 56
4.1. The Mesolithic-Neolithic transition 56
4.2 Eneolithic (5th millennium BC) 57
4.3 Late Eneolithic and Early Bronze Age (4th millennium BC) 57
4.4 From Early to Middle Bronze Age (3rd millennium BC) 58
4.5 Final Middle and Late Bronze Age (2nd millennium BC) 59
4.6 Y-chromosome haplogroup diversity through time 60
4.7 Mitochondrial haplogroup diversity through time 61
5 Supplementary References 63

1 Archaeological background of the Caucasus cultures

Since ancient times, the Caucasus has been considered one of the most diverse mountainous regions in Eurasia. It is undoubtedly a central interface between the ancient civilizations of Southwest Asia and the cultures of Europe and the Eurasian steppe zone. Alongside its geographical integration, high biodiversity and diverse ecotones characterises the various habitats in the lowlands, foothills, and high mountains[1,2]. Glaciers cover the highest peaks of the Greater Caucasus in the north. This results in a tight network of drainage systems like the Kuban and Terek rivers. They drain into the Black Sea and the Caspian Sea respectively. The Lesser Caucasus and the adjacent highlands are more continental. Nevertheless, the headwaters of rivers like Kura, Araxes, Euphrates and Tigris are situated here. Caucasia thus geographically connects the distant poles of Eurasia and Ancient Mesopotamia with its diverse cultures[3]. They emerged in the interplay of global and local processes and were repeatedly characterised by migrations from outside and within.

One of the pull factors in populating the Caucasus were its rich mineral resources. Obsidian has been extracted from volcanic sources in the South Caucasus since the Neolithic. It was traded via extensive networks to the south, west and east[4]. Metals like copper but likewise gold and silver, precious stones, and salt were exploited from the 5th and 4th millennia BC onwards[5]. We can also surmise perishable trade goods such as timber, herbs, skins, furs, and others. Resource extraction was closely integrated into finely tuned networks of agricultural settlements, mining zones, pastoral territories and routes, as well as small-scale and long-distance trading networks.

1.1 Archaeological background and chronology

The Caucasus is not only characterised by a high level of biodiversity, but also by great diversity in the cultures present in the region[6,7]. Both of these phenomena are a likely result of its small-scale geography. This study focuses on the cultures of the Greater Caucasus, the bordering steppe region to the north, and the southern intermontane zone, from the Neolithic to the Late Bronze Age (Fig. 1a). This includes the Caucasian mountain Neolithic (6100-5650 BC), South Caucasian Neolithic or Shomutepe Shulaveri culture (6200/6000-5400 BC), the Darkveti-Meshoko Eneolithic (4900/4700-3900 BC), the Steppe Eneolithic (4350/4300-4100/4050 BC), the Maykop phenomenon of the North Caucasus (3950/3900-2900 cal. BC), the South Caucasus Kura-Araxes culture (3600/3500-2500 BC), the North Caucasian cultural formation (2850/2800-2400 BC), the Yamnaya (3300-2600/2500 BC) and Catacomb grave or Catacomb cultural communities (2900/2800-2200 BC), several cultural groups of the post-Catacomb horizon such as the Lola, Kuban and Arkhon groups, as well as the Ginchi culture in mountainous Dagestan (2300/2200-1700/1650 BC), and several LBA formations such as Srubnaya (1900-1200 BC), Belozerka (1200-1000 BC) and local LBA groups in the mountains (1700/1550-1000 BC). From these cultures we assembled the time slices for this study according to the average dating and the logic of the genetic groupings. Some of the cultures have an offset with regard to the given timeframe for these time

periods (Fig. 1b), since the respective cultures have an individual start and end. The archaeological and genetic affiliation of each individual is given therefore in Supplementary Table 1.

The dating of all cultural groups is supported by the 78 new radiocarbon dates (Supplementary Table 1) and the current literature, with the new data largely confirming previously published time estimates. In some cases, however, we were able to be more precise with our data. We report the radiocarbon dates as unmodeled data with a 95.4% (2σ) calibration interval and median value.

Early Holocene human occupation is associated with hunter-gatherer groups of the Mesolithic, and Epipaleolithic or local Neolithic[8,9]. While the number of radiocarbon dates is still relatively few, the Mesolithic can be dated to a wider interval of 12000 to 6200 BC, and the Neolithic sites in the mountains can be dated to between 6100 and 5650 BC, based on dating from Mezmayskaya cave[8] and the Cmi open air site[10]. The definition of this local Neolithic period, and its stone tool assemblages, dating, and the integration into the wider frame of Epipaleolithic and Pre-Pottery Neolithic cultural trends remains under discussion. Along with the two Mesolithic individuals from Satsurblia and Kotias klde[11], the human remains from Satanaj Grotto are central to this study. This individual dates to 6223-6080 BC and is thus situated between the latest Mesolithic date of Cmi dating to 6564-6257 BC (LuS-7763: 7570±50 BP) and the earliest Neolithic dates from Mesmayskaya 6067-5760 BC (SPb-1339: 7060±80 BP) and Cmi 5990-5757 BC (LuS 7762: 7010±50 BP). This individual lived in a transitional period between Mesolithic and local Neolithic traditions in the Northwest Caucasus. These local Neolithic traditions included the first pottery[8,10] and possibly also the first domesticates arriving in these mountain hunter-gatherer communities[12]. Nevertheless, they are like other Neolithic traditions of the Eurasian forest, forest steppe and steppe[13], an independent development and must be separated from the Neolithic of West Asian character that was introduced in the Caucasus by immigration[14,15]. The Eurasian Neolithic, like the Near Eastern Neolithic, witnessed far-reaching changes in the economy and in the spectrum of artefacts, yet the economy remained one of foraging, including a seasonally mobile way of life[16]. Key artefacts are flat-based and pointed-based pottery, which were developed as early as the 8th and 7th millennia BC in Northern Eurasian, and which differ significantly from the ceramic introduced to the South Caucasus from Near Eastern traditions[17]. The intertwining of these two Neolithic lifeways started with the advance of Near Eastern Neolithic groups into the South Caucasus and continued at its north-western and north-eastern periphery during the 5th millennium BC. The actual adaptation processes and their timing are still difficult to track but they are decisive for the question of whether, and to what extent, Neolithic cultural techniques spread by means of knowledge transfer and local adaptation, or by migration.

The Neolithization of the South Caucasus and the related Shomutepe-Shulaveri culture is meanwhile well studied and dated[15,18]. First settlements with circular mudbrick architecture are found dating to ca. 6200 BC, whereas most of the sites date between 6000 and 5400 BC. Culturally, the actual immigration process is still not fully understood, as there are major gaps and considerable differences between the presumed area of origin in Northern Mesopotamia and the settlements in the valleys of Araxes and

Kura. However, these groups established a fully Neolithic economy with agriculture and animal husbandry for about 500 years. Following this, their settlements disappeared[19]. The Eneolithic cultures of the 5th millennium BC in the South Caucasus remain vague and are difficult to identify[20]. The six individuals in this study originate from the key Neolithic sites of Arukhlo 1 and 2 and were found scattered in the cultural debris of the sites.

The North Caucasian Eneolithic is divided into the Darkveti-Meshoko Eneolithic (4500-4000 BC) in the mountain foothills[21] and the Steppe Eneolithic (4350/4300-4100/4050 BC) in the lowlands[22]. The first cultural grouping is characterised by small and partly fortified settlements on hills and promontories. It is the first Neolithic culture of West Asian character in the north. Animal bone assemblages[23] and first archaeobotanical studies[24] confirm an agro-pastoral economy. The second Eneolithic cultural formation is characterised by low burial mounds dispersed in the steppe zone between Lower Don, Lower Volga and the North Caucasus. The dead covered densely with red ochre are emblematic. We could enlarge our former sample with additional nine individuals that passed genetic quality thresholds from the areas along the northern Caucasus-Steppe interface. Among these are several individuals from the westernmost site of Khutor Beliyy, situated near the Black Sea coast, which were found in a mass grave. Additionally, we were able to successfully sample two individuals from the largest cemetery of this epoch at Nalchik. This key site is situated in the central North Caucasus foothills and its inventories have close parallels to the Lower Volga Eneolithic with its central site of Khvakynsk[25].

Meanwhile 38 individuals, of which 20 are new, represent the Maykop epoch (3950/3900-2900 BC)[6]. Their radiocarbon dates confirm an earlier (3950-3400 BC) and later (3400-2900 BC) sub-phase, yet the newly dated complexes substantialized a mid-position between ca. 3650-3400 BC. The archaeological material provides few indicators for this middle group. It is possible that it is the result of the integration of two plateaus on the radiocarbon calibration curve, which makes more precise dating difficult in the 4th millennium BC[26]. As all Maykop_main individuals have rather homogeneous genetic profiles, for our study a more precise chronological discussion is not crucial. We used terms like ‘early’ and ‘late’ for the first and the second half of the 4th millennium BC (Supplementary Table 1). Among these, we find individuals from burial mounds in the piedmont zone with classical Maykop inventories e.g. from the sites of Essentukskiy 1 or Vinsady 4 and 5. Conversely, corresponding material culture and burial practices are also found among burials in the steppe, such as the graves from Aygurskiy 2[27] or Kurganny 1. However, graves with Eneolithic traditions in burial practice but 4th millennium BC dates have been noted e.g., from Konstaninovskiy 4, Ipatovo 3, and Zolotarevka 1. This epoch reflects a period where some cultural elements such as Maykop pottery, metal artefacts or socially stratified burial practices were shared over wide areas[28], whereas regional traditions were sometimes preserved. We coined from this a Maykop main and a Steppe Maykop variant[29], which still holds from a cultural point of view. However, we must also note that defining archaeological cultures by artefact combinations and burial practices is at its limit, especially when considering the high genetic diversity

of the populations of this epoch. We cannot establish correlations of cultural ensembles with specific biological groups, nor do any genetic groups in our 4th millennium BC dataset correlate directly with Late Eneolithic or Early Yamnaya traditions postulated from respective burial practices[30]. Economic practices such as using sheep for making dairy-products were shared[31], while pastoral areas were divided between steppe and piedmont territories[32]. The later phase of the Maykop culture is, however, the time when wide-ranging networks between Central Asia, the Near East and the Pontic area were established in which new technologies and new objects such as massive metal tools from alloyed copper[33], new social displays[34] and the technologies related to early mobile pastoralism were circulating and spread beyond the actual Caucasus area.

Evidence for the Yamnaya cultural formation (3300-2600/2500 cal. BC) from the North Caucasian steppe zone was strengthened by the analysis of seven new individuals. Among these are three graves from the Krasnogvardeyskoe mound, with one representing a wagon burial[35]. These Yamnaya graves correspond well to comparable burials further west[36]. Their dating between 3330 and 2630 BC also matches the time frame currently accepted for this period. In this study we increased the number of Kura-Araxes individuals from the South Caucasus by seven individuals from the Balitshi-Dzedzvebi site, a worker's settlement for processing the gold from the world's oldest discovered gold mine at Sakdrisi, Georgia[37]. Kura Araxes sites in the Caucasus are dated between 3600/3500 and 2500 BC with a double or tripartite internal division[38]. The dates for Balitshi-Dzedzvebi fall into the earlier phase and range between ca. 3600 and 2900 BC[37]. The studied burials are dated in the centuries around 3000 BC (Supplementary Table 1). This period witnessed the start of the expansion of Kura-Araxes communities. They played a key role in the diffusion of innovations and technologies like wheeled transport[39] and massive metal objects, as well as new economic practices such as wine making, into the areas southwest and southeast of the Caucasus[40].

The North Caucasian cultural groups (2850/2800-2400 BC) in the piedmonts and mountains, together with the nearly contemporaneous Catacomb culture (2900/2800-2200 BC), represent the mobile MBA communities in the central and western mountains and piedmonts as well as the North Caucasus steppe, whereas in the north-eastern Dagestan local mountain dwellers resumed own agro-pastoral lifeways since the Eneolithic and EBA. The number of studied MBA individuals is supplemented by 48 new ones, with a major group originating from the big mound of Komsomolec 1-Marfa. In this 10 m high barrow a chronological sequence from the late Maykop to the Sarmatian era was excavated (Extended Data Fig. 8b). The MBA graves are proper North Caucasian graves with characteristic features such as stepped burial shafts with wooden ceilings, burials in supine positions and inventories typical for this epoch. But Catacomb type of burials with deceased in flexed positions exist as well. In some graves a blending of these features was observed. Radiocarbon dates from these graves result in a chronological sequence with a transitional period when both shaft and catacomb graves were used. This confirms earlier observations in these mounds[41] (Extended Data Fig. 8a).

One focus of this study is the exploration of the processes that led to the genetic formation of today's mountain populations in the Greater Caucasus[42,43]. This begins with a cultural horizon that has only been outlined in the last decade. The so-called post-Catacomb horizon (2300/2200-1700/1650 BC) characterises an epoch of cultural fragmentation in which numerous regional groups with local characteristics emerged[44,45]. At the same time, close links between the mountains and the steppe can be identified. They are interpreted as indicators of migration but could also be the manifestation of intensified exchange networks[46]. The transformation in metallurgy from arsenic-alloyed to tin-alloyed bronzes is highlighted as another indication of changing interaction spheres, and this is the time in which domesticated horses rapidly spread all over Eurasia and the Near East[47]. The relevant groups are the Lola culture situated in the steppe zone, the Kuban group along the lower course of the Kuban River, and the Arkhon group in the central foothills of the Greater Caucasus. We included an additional 16 individuals from sites such as Poselenie Kravchenko in the western Kuban area, to the central parts of the North Caucasus foothills near Magas in Ingushetiya. The majority of the burials in the Ginchi necropolis and the related agro-pastoral culture in the high mountains of Dagestan date to the same period, from which we studied eight individuals. Our data indicates, however, a longer use of the crypts between 2400 and 1550 BC.

Finally, after ca. 1700/1600 BC, the LBA emerged as a cultural horizon in the high mountain zone of the Great Caucasus[45]. Its transition to the Early Iron Age is dated to ca. 1100 BC. In the South Caucasus, LBA groups date from 1550 BC onwards[48]. During this epoch, resettlement and the transformation to a highly efficient combined mountain economy paved the way for the emergence of complex societies and growing social stratification[49,50]. For this epoch we have little evidence for burials from the North Caucasus with only three newly studied individuals. But a series of five LBA graves from the South Caucasus site of Lernakert associated with the Lchashen-Metsamor culture (1530-1150 BC) were included in this study. Isolated Srubnaya and Belozerka complexes have been recorded along the Kuban dating to the mid-2nd millennium BC and represent the last Bronze Age steppe populations in the area and added two new individuals to our study. Both Srubnaya and Belozerka cultures are phenomena native to the Pontic Steppe and the Forest Steppe[51,52].

1.2 Changing economies of Neolithic, Eneolithic, and Bronze Age Caucasus

Three major tendencies are fundamental for the economies between 6200 and 1000 BC. On the one hand, they form the background to Caucasus population dynamics, but on the other hand also initialise some of them. This is the introduction of the Neolithic way of life with agriculture and animal husbandry, including West Asian domesticated animals and plants by ca. 6200/6000 BC[18,53]. The second major process is the emergence of mobile pastoralism, namely the combination of a herding economy in steppe environments based on the exploitation of grassland adapted herbivores with an intensive use of dairy products[31], a mobile lifestyle maintained by wooden wagons[41,54] and the successive domestication of horses[47], and mobile architecture using wool and plant materials[55]. This process started

around 4300 BC, accelerated from 3500 BC and made it possible to advance into the dry zones of the Eurasian steppe after 3300/3000 BC. In the area we studied, only the high mountains of Dagestan make an exception. Mountain agriculture developed there in the 4th millennium BC at the latest, and was based on extensive agricultural terracing[56]. The third economic transformation started in the highlands at around 1700/1600 BC. The mobile pastoral economy was transformed into a semi-stationary, combined mountain agriculture[50]. It enabled an intensification of animal husbandry, the development of territorially anchored communities, a growing exploitation of metal and mineral resources, and resulted in a general increase in archaeologically verifiable sites during the Late Bronze Age (LBA). At this time, the archaeological evidence of habitation in the steppes north of the Caucasus disappeared for more than a millennium[57]. The reason for this were probably the after-effects of the 4.2 ka BP climate event, which were accompanied by an aridification of the steppe[58]. These presumably continued to have a negative impact on grassland productivity afterwards.

1.3 Archaeological cultures and genetic groupings

The challenges of correlating archaeological and genetic terminology are meanwhile a recognized problem[59] as well as the risk of transferring outdated essentialist and ethnic concepts with a classification of genetic groups based on archaeological terms[60]. The identification of genetic ancestry profiles is based on clearly defined criteria and can be validated by statistical tests[61]; the criteria for archaeological 'cultures' are the interplay of various elements such as similarities in materiality, concurring cultural and economic practices. Due to these fuzzier criteria, which must be discussed on a case-by-case basis, the concept of archaeological culture is meanwhile perceived rather skeptically[62]. Our case study demonstrates that there is no linear correlation between specific genetic ancestry profiles and archaeological groups or cultures. We have therefore decided to systematically use the archaeological and genetic group designations on an equal footing (Supplementary Table 1). This enables us to dissolve the biological determination of archaeological phenomena and to approach the actual social interactions.

2 Archaeological site and sample descriptions

The information for sites and complexes is kept to a minimum, but is critical for understanding the context of the studied individuals. Only find complexes that are part of the genetic analysis have been outlined. Several complexes have multiple radiocarbon dates. In these cases, the date highlighted in bold in the catalogues of the site descriptions below is considered the most appropriate based on contextual stratigraphic information or archaeological parallels and is thus used for further analysis in this study. This date and its quality criteria are indicated in the Supplementary Table 1. The specific sample material that was used for ^{14}C dating is indicated in the catalogue and Supplementary Table 1 by: ab – animal bone, hb – human bone, charcoal or leather remains. With the exception of two all new

radiocarbon dates reported in this paper were generated at Curt-Engelhorn-Center for Archaeometry e.V. CEZA, Mannheim, Germany. According to the CEZA protocol, the ^{14}C content was measured using a MICADAS AMS system. In total, we report 84 new radiocarbon dates that were used to contextualise the findings and to refine the regional chronologies. The $^{14}C/^{12}C$ and $^{13}C/^{12}C$ isotope ratios of samples, calibration standards (oxalic acid II), blanks and control standards were measured simultaneously. The determined ^{14}C ages are normalised to $\delta^{13}C$=-25‰[63] and calibrated using IntCal20[64] and the Oxcal software v4.4.2. For better readability we report the OxCal 'whole range' option with probability ranges of 68.3% and 95.4%. The 95.4% probabilities are given as start and stop values in Supplementary Table 1.

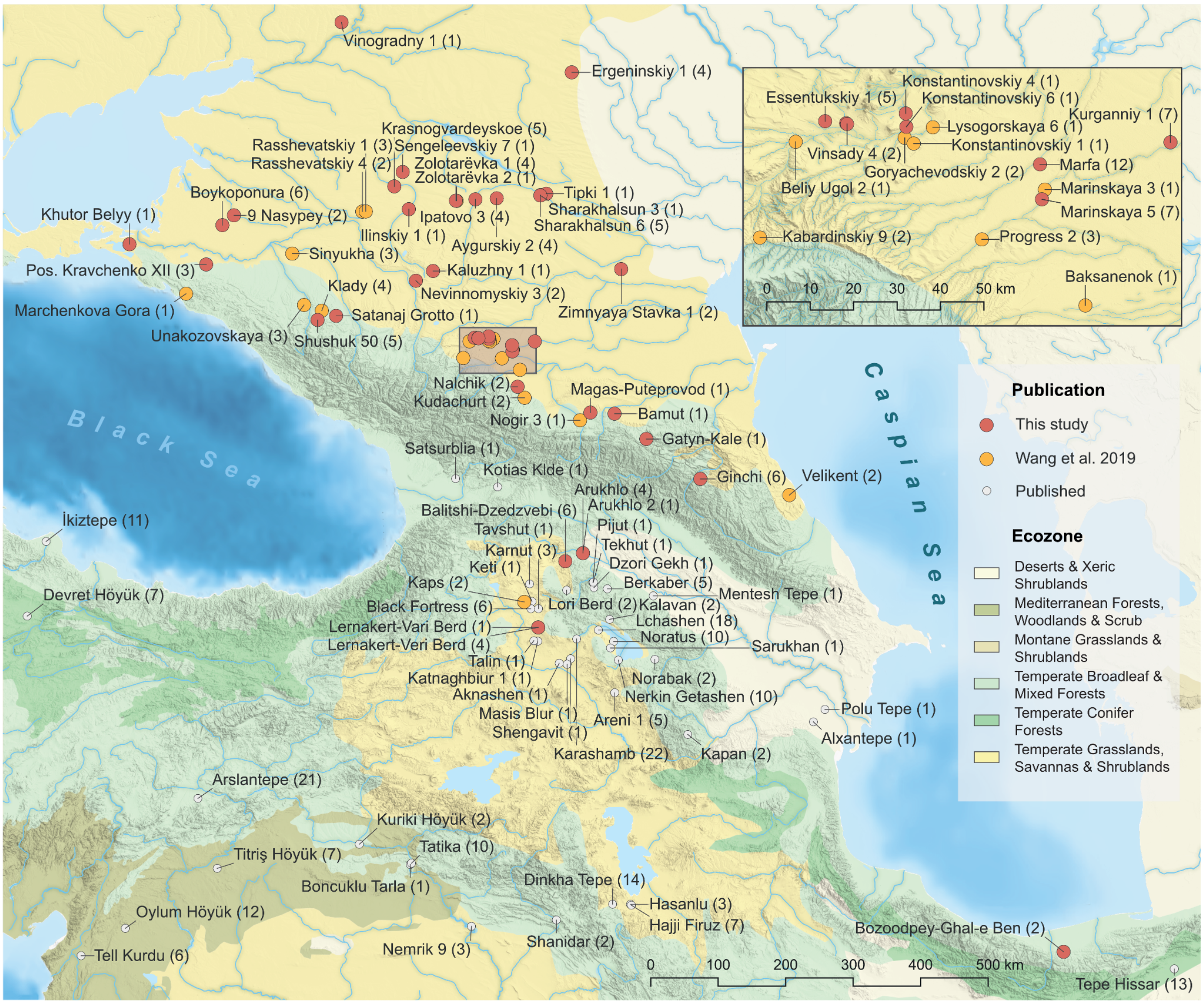

Supplementary Fig. S1. Archaeological sites and ecozones of the Caucasus. Map of the wider Caucasus region showing the locations of the sampled sites with full site names and number of individuals in parentheses (red: new data, yellow: published data[29], white: other relevant published data (Supplementary Table 1 and 5). The map was generated using Base Relief: Mapzen, OpenStreetMap, and rivers, lakes and borders were added using free vector and raster map data from naturalearthdata.com, and shows the ecozones of the wider Caucasus region adapted from https://ecoregions.appspot.com/. OpenStreetMap is open data, licensed under the Open Data Commons Open Database Licence by the OpenStreetMap Foundation.

2.1. Armenia

2.1.1 Lernakert

Country: Armenia

Region: Shirak region

Coordinates: 40.54902°, 43.94583°

Excavation details: Excavation of the Institute of Archaeology and Ethnography NAS RA (license №2019-13, Benik Vardanyan), Shirak Armenology Research Center of Armenian Academy of Sciences

Site information: The archaeological site of Lernakert is located in the Shirak province, Republic of Armenia, at an altitude of ca. 1980 m above sea level (asl) on the north-western slope of Mount Aragats. The region around Lernakert is a mountainous landscape of volcanic origin, with volcanic cones and rock outcrops. The landscape is rich in montane grasslands and shrublands, springs, and seasonal streams. The archaeological complex of Lernakert includes monuments of different phases of the Bronze and Iron Ages[65]. Among them are two fortresses (Veri Berd, Vari Berd) and settlements with layers mainly from the Late Bronze Age (14th-13th cent. BC) and the Classical epoch (~500-100 BC). Across the entire area clusters of burials are located, which are formed of stone and earthen barrows with stone-circles (cromlechs) of up to 30m diameter. Some stand separately, whereas others are connected but separated from neighbouring clusters by low walls. During surveys in 2019-2022 more than 500 structures were documented.

Between 2019 and 2023, 26 burials were excavated, which date to the Late Bronze Age and the Early Iron Age. Five of them are included in this study, originating from clusters № 1, № 2 and № 4). The first cluster is located to the east of the upper fortress Veri Berd and is fenced in by a partly preserved low wall. One group of graves was constructed with stone and earth mounds; the other group consists of stone mounds with grave chambers covered with 3–5 capstones. Grave cluster № 2 is located on the northeast slope of the same fortress. Tombs are pit graves with cromlechs and covered with 3–5 capstones. Group № 4 is located north of the fortress, on the northern slope of a hill running east-west. This grave cluster is surrounded by a partly preserved fencing wall, inside which the burial mounds were constructed with stones and earthen grave chambers.

- **LER001**, LKVeB19-2-1, Veri Berd, Cluster № 2, Grave 2. *Context information*: The burial was situated in spiral-shaped cromlech with a diameter of 9 m and three capstones on top of the southeast northwest-oriented chamber grave. The skeleton of a 9(±2)-year-old child burial was found in crouched position on the left side, with the head to the northwest, was badly preserved. Animal bones of a cattle and an obsidian arrowhead were found east of the skull. Five complete vessels and two beads were uncovered in the tomb. *Cultural affiliation & dating:* Late Bronze Age, Lchashen-Metsamor culture, MAMS-66472: 3061±15 BP (cal. BC 1385-1286, cal. BC 1398-1266), hb.

- **LER002**, LKVaB19-3-1, Veri Berd, Cluster № 1, Grave 3. *Context information*: Inhumation in a pit grave with a diameter of 6.20 m. The burial chamber is oriented east–west, with the partly preserved skeleton of a 20-29-year-old woman not in anatomical order. Two vessels were found near the lower limbs of the skeleton. *Cultural affiliation and dating*: Late Bronze Age, Lchashen-Metsamor culture, MAMS-66471: 3074±15 BP (cal. BC 1396-1297, cal. BC 1408-1282), hb.
- **LER003**, LKVaB20-3-2, Vari Berd, Grave 1. *Context information*: Inhumation below large caps tones in a stone mound of 5.7-5.4 m diameter. The inhumation, a 20-29-year-old female individual, was found in the initial grave-pit but not in anatomical order. Instead, the extremities were placed side by side and the upper body parts were likewise arranged artificially. The grave was obviously a secondary burial. Grave goods included three pottery vessels. *Cultural affiliation & dating:* Late Bronze Age, Lchashen-Metsamor culture, MAMS-66473: 3058±15 BP (cal. BC 1383-1284, cal. BC 1398-1263), hb.
- **LER004,** LKVeB20-5-1, Veri Berd, Cluster № 4, Grave 5, individual 1. *Context information*: The burial was found in a circular cromlech with a diameter of 6.5 m and a stone mound of 0.4m height. The burial pit was oriented east–west and covered by five capstones and included inhumations of two individuals without anatomical order. Individuals 1 is a 30-35-year-old female. The individuals' ulna and clavicles were located under the skull. Carnelian beads were found under the mandible. The second individual was very poorly preserved. Two bowls were uncovered in the eastern part of the chamber, east of the skull of individual 1. *Cultural affiliation & dating:* Late Bronze Age, Lchashen-Metsamor culture, MAMS-66462: 3068±15 BP (cal. BC 1391-1291, cal. BC 1403-1274), hb.
- **LER005,** LKVeB20-10-1, Veri Berd, Custer№ 4, Grave 10. *Context information*: The burial was found in a circular cromlech with a diameter of 6 m, with an east–west oriented burial pit, covered by two capstones. Remains of an inhumation of a 40-49-year-old male individual without anatomical order. In the eastern part of the chamber three bowls and scattered bones were found, A large pitcher was placed below the western head wall of the tomb. *Cultural affiliation & dating:* Lchashen-Metsamor culture. Late Bronze Age, Lchashen-Metsamor culture, MAMS-66463: 3079±14 BP (cal. BC 1399-1302, cal. BC 1411-1287), hb.

2.2. Georgia

2.2.1 Arukhlo 1

Country: Georgia
Region: Kvemo Kartli
Coordinates: 41.46960°, 44.67920°

Excavation details: Excavation DAI Eurasia-Department & Georgian National Museum, Othar Lordkipanidze Centre of Archaeological Research, 2005-2016

Site information: The Neolithic site Arukhlo 1, dating to 5800-5400 BC, is situated only a few 100 m north of the terrace edge of a river floodplain about 50 km southwest of Tbilisi in present-day Georgia. Here, the Chrami and Mashavera rivers, which rise in the mountains of the Lesser Caucasus, join and flow a little further east into the Kura River, which in turn drains into the Caspian Sea. The archaeological excavations at Arukhlo 1 started in 1966 under the direction of T. N. Chubinishvili, and was continued from 1978 to 1985 by D. Gogelia. The work was resumed from 2005 to 2016 as cooperation between the Eurasia Department of the German Archaeological Institute and the Archaeological Centre of the Georgian National Museum.

Arukhlo 1 is the northernmost settlement of the so called Shulaveri-Shomutepe culture, which was dominant in the region during the 6th millennium BC[14]. It is a tell site formed from accumulated architecture and cultural layers. Characteristic are building complexes consisting of at least two to three round houses built of plano-convex unfired yellow and brown mud bricks. Their diameter ranges from 1.8 m (storehouses?) to 6 m (dwellings?), and are plastered with a 1-2 cm thick layer of clay on the outside. Striking are the numerous ditches of unknown function (defence, water supply, ritual activities?) running through the settlement. These have been documented at Arukhlo 1 for the first time in the South Caucasus. Their maximum depth is up to 4.8 m with a width of 4.5 m. The ditches cut through several settlement layers, traversing the settlement in irregular windings and slopes, and were backfilled and re-excavated after a short time. Some small circular buildings were built on top of backfilled ditches.

The human bones reported here have been found as small pieces in several areas across the site. They were deposited in the cultural layers or in pits and selected from the animal bone assemblages. The origin of these fragmented human bones is unknown. Individual or collective graves exist at other sites of the same period, but none were found at Arukhlo so far. Here only a cremation burial has been documented in one of the buildings.

In total we collected samples from 14 individuals, of which five yielded sufficient ancient human DNA for further analyses (Supplementary Table 1). The remaining nine samples did not yield sufficiently high amounts of human DNA molecules and were thus not further pursued after initial SG screening (Supplementary Table 2).

- **ARO006,** BZNK-0913/1, Arukhlo settlement. *Context information:* Human bones from a cultural layer, context AR11C082, *Cultural affiliation & dating:* Shulaveri-Shomutepe culture, MAMS-42356: 6699±22 BP (cal. BC 5637-5567, cal. BC 5664-5561), hb.
- **ARO008,** BZNK-0915/1, Arukhlo settlement. *Context information:* Human bones from a pit in cultural layer NL07, *Cultural affiliation & dating:* Shulaveri-Shomutepe culture, MAMS-42357: 6550±25 BP (cal. BC 5530-5477, cal. BC 5608-5474), hb.

- **ARO011,** BZNK-0918/1, Arukhlo settlement. *Context information:* Human bones from a pit in cultural layer NL06. *Cultural affiliation & dating:* Shulaveri-Shomutepe culture, MAMS-42358: 6676±25 BP (cal. BC 5627-5565, cal. BC 5641-5536), hb.
- **ARO012,** BZNK-0919/1, Arukhlo settlement. *Context information:* Human bones from a cultural layer, context AR11C082, *Cultural affiliation & dating:* Shulaveri-Shomutepe culture.
- **ARO013,** BZNK-0920/1, Arukhlo settlement. *Context information:* Human bones from cultural layer NL08. *Cultural affiliation & dating:* Shulaveri-Shomutepe culture, MAMS-42359: 6590±22 BP (cal. BC 5605-5483, cal. BC 5612-5480), hb.

2.2.2 Arukhlo 2

Country: Georgia

Region: Kvemo Kartli

Coordinates: 41.46960°, 44.67920°

Excavation details: Excavation DAI Eurasia-Department & Georgian National Museum, Othar Lordkipanidze Centre of Archaeological Research (S. Hansen, G. Mirtskhulava), 2012-2013

Site information: In the immediate vicinity of Arukhlo 1 were two other small archaeological sites, of which only one (Arukhlo 2) still existed in a heavily destroyed form. A brief archaeological investigation was carried out in 2012/13. The few finds confirmed a contemporaneous occupation to Arukhlo 1. The archaeological material is unpublished.

We collected two human bone samples, of which one yielded sufficient ancient human DNA for further analyses and is reported here. The second sample did not pass the quality criteria of the initial SG screening (Supplementary Table 2).

- **AO2001,** BZNK-0924/1, Arukhlo 2 settlement. *Context information*: Human bones from settlement layers. Cultural affiliation & dating: Shulaveri-Shomutepe culture, MAMS-42360: 6943±23 BP (cal. BC 5878-5772, cal. BC 5887-5741), hb.

2.2.3 Balitshi-Dzedzvebi

Country: Georgia

Region: Bolnisi municipality

Coordinates: 41.3718488°, 44.3915731°

Excavation details: Excavation Deutsches Bergbau Museum & Georgian National Museum, Othar Lordkipanidze Centre of Archaeological Research (Th. Stöllner, I. Gambashidze).

Site information: The Dzedzvebi Plateau is situated south of the village of Balichi and the small town of Kazreti in southeast Georgia[37,66]. The basaltic plateau stretches 1730 m, from 708 m asl to 768 m asl. The elongated plateau lies between the Mashavera and the Ukangorula (Dampludka) rivers, dominating the intermontane valleys south of Kazreti. At its widest point, the plateau is approximately 400 m wide, while at its narrowest it is just over 100 m wide. Due to its location, it also cordons off the north-south

connection of the valley. On its northern side, the small Dampludka River flows into the Mashavera, naturally protecting access to the northern plateau. To the south, the plateau between Mashavera and Ukangorula (Dampludka) narrows considerably.

The terrain gently rises towards the south, where it has been protected by a fortification rampart since the Iron Age at the latest. The plateau thus encloses a settlement area of approximately 60 ha and can be divided into four distinct areas. In the north, the plateau ends in a spur-like plain (Area I) with an Iron Age settlement and an access road on its eastern slope, which continues to provide access from the northeast to this day. A relatively broad slope area rises slightly to the south (Area II), towards the almost flat, narrower Area III, which eventually merges into the southern plateau (Area IV) after a first plateau constriction from both river valleys in the west and the east.

Several settlement nuclei were established at different locations on the plateau during different chronological phases. So far, a Chalcolithic settlement of the 5th millennium BC has only been found on the southern plateau, but not in other excavation areas (areas IV.1–2, IV.4–6) on the southern plateau. This suggests that settlement activity in the Chalcolithic was certainly lower than in the Iron Age. The next chronological focus is the Kura-Araxes occupation, which can be dated between 3700-2600 BC. These settlements are mainly distributed on the southern plateau (Area IV) and along the slopes of Dzedzvebi II and the northern part of Dzedzvebi III. This area can be characterised as a settlement dominated by crafting activities with smaller, interspersed burial groups. Its temporal overlap with mining activity at Sakdrisi and additional evidence of artefact and metallurgical features (e.g., stone tools, crucibles, workshops). The settlement is related to the copper and gold exploitation of the Madneuli-Sakdrisi deposit district. Isolated Middle Bronze Age (MBA) occupations have also been found in the same area, mainly represented by Trialeti burials and ritual deposits. In contrast to the settlement in Areas II and III, the Kura-Araxes occupation of the southern plateau is difficult to assess. Like in Dzedzvebi II and III, evidence of occupation during the oldest Kura-Araxes period, dating to the early 3rd millennium BC, has been found on the southern plateau, represented by late Kura-Araxes pottery in the style of Shengavit ware. A few indications of MBA occupation have been found there as well, but no regular pits but some unspecific architectural features from this period have been identified thus far. The Iron Age occupation of the northern plateau from ~1100 BC onwards has not yet been investigated, but significantly disturbed the older Chalcolithic and EBA settlement features, mainly due to its deep storage pits.

- **A19270**, Area II.4, Collective burial 6, Individual 19. *Context information*: collective burial, female individual, bone package at the western end of the chamber. *Cultural affiliation & dating:* Kura-Araxes, MAMS-24766: 4378±23 BP (cal. BC 3016–2928, cal. BC 3085–2915), bone, MAMS-24767: 4401±22 BP (cal. BC 3086–2934, cal. BC 3090–2926), bone[37].
- **A19278**, Area II.4, Collective burial 6, Individual 24. *Context information*: collective burial, female individual, bone package at the western end of the chamber. *Cultural affiliation &*

dating: Kura-Araxes, MAMS-24766: 4378±23 BP (cal. BC 3016–2928, cal. BC 3085–2915), bone, MAMS-24767: 4401±22 BP (cal. BC 3086–2934, cal. BC 3090–2926), bone[37].

- **A19269.73.74**, Area II.4, Collective burial 6, Individual 15. *Context information*: collective burial, main crouched burial, male. *Cultural affiliation & dating:* Kura-Araxes, MAMS-24766: 4378±23 BP (cal. BC 3016–2928, cal. BC 3085–2915), bone, MAMS-24767: 4401±22 BP (cal. BC 3086–2934, cal. BC 3090–2926), bone[37].
- **A19271.79**, Area II.4, Collective burial 6, Individual 21. *Context information*: collective burial, male individual, bone package at the western end of the chamber. *Cultural affiliation & dating:* Kura-Araxes, MAMS-24766: 4378±23 BP (cal. BC 3016–2928, cal. BC 3085–2915), bone, MAMS-24767: 4401±22 BP (cal. BC 3086–2934, cal. BC 3090–2926), bone[37].
- **A19268**, Area III.1, Collective burial 3, Individual 5. *Context information*: collective burial, main crouched burial, male. *Cultural affiliation & dating:* Kura-Araxes, MAMS-24764: 4333±22 BP (cal. BC 3008–2902, cal. BC 3012–2899), bone[37].
- **A19272**, Area III.1, Collective burial 3, Individual 11. *Context information*: collective burial, female individual, bone package at the northwestern end of the chamber. *Cultural affiliation & dating:* Kura-Araxes, MAMS-24764: 4333±22 BP (cal. BC 3008–2902, cal. BC 3012–2899), bone[37].

2.3 Russian Federation

2.3.1 9 Nasypey

Country: Russian Federation
Region: Timashevsk district, Krasnodar region
Coordinates: 45.49304°, 38.95225°
Excavation details: Expedition 'Research Institute of Archaeology and Ancient History of the North Caucasus', Stavropol 2017, licence № 2017-2206 (S. V. Myachin)
Site information: The barrow cemetery Nasypey 9 is located in the centre of the Krasnodar region. The grave mounds are situated at the left flank of the watershed along an old riverbed of the Kirpili river, approximately 200 m southeast of the village Derbentskiy. The site was discovered in 1992 and was registered under the name “Barrow group 9 Nasypey”. During construction work at the nearby railway line in 2014 barrows 19 and 20 were excavated. Prior to excavation mound 19 had a height of 0.22-0.61 m with a diameter of 45 m. In the barrow 11 graves were discovered. Grave 7 is a late Yamnaya grave dug into the first mound shell. Burial mound 20 was heavily ploughed. Before excavation, the mound was only 0.05-0.16 m high and had a diameter of 30 m. It was built in one construction phase. This mound was built on top of Yamnaya grave 3 in the centre of the mound. The other four burials in the mound were of equal or later date. The archaeological material is unpublished.

Samples from four individuals were screened (Supplementary Table 2), of which three produced genome-wide data (Supplementary Table 1):

- **9NP002,** BZNK-1036/1, kurgan 19, grave 7. *Context information*: Single inhumation in a rectangular grave-pit with rounded corners, originally probably with a wooden cover (organic remains); individual in supine extended position with his knees slightly bent to the right, head towards the northwest, ochre and charcoal. *Cultural affiliation & dating:* Late Yamnaya (according to the report).
- **9NP003,** BZNK-1037/1, kurgan 20, grave 3, individual 1. *Context information*: Multiple inhumations with four individuals in an irregular rectangular grave-pit, at the bottom 1.62 x 1.20 m. The skeleton of individual 1 was poorly preserved and was found in a crouched position on the left side, with the head in the north. Fourteen perforated astragals were found scattered between all four skeletons. *Cultural affiliation & dating:* Yamnaya, MAMS-42345: 4205±19 BP (cal. BC 2886-2707, cal. BC 2891-2700), hb; **MAMS-51685: 4202±16 BP (cal. BC 2883-2708, cal. BC 2887-2702), ab**.
- **9NP004,** BZNK-1038/1, kurgan 20, grave 3, individual 3. Context information: the skeleton was poorly preserved. *Cultural affiliation & dating of both individuals:* Yamnaya, MAMS-42346: 4200±19 BP (cal. BC 2884-2707, cal. BCE 2890-2697), hb; **MAMS-51685: 4202±16 BP (cal. BC 2883-2708, cal. BC 2887-2702), ab**.

2.3.2 Aygurskiy 2

Country: Russian Federation

Region: Apanasenkovo district, Stavropol region

Coordinates: 45.69000°, 43.26200°

Excavation details: Excavation 'Nasledie' 1998-2001, Stavropol, licence № 1998-176; №1999-804; №2000-377 & №2001-776 (V. A. Babenko)

Site information: The Aygurskiy burial mound, cemetery 2, was situated on a promontory overlooking the small river of Aygurskiy, about 22 km east of the city of Ipatovo. The area is a slightly hilly landscape, today in a desert steppe environment. However, as shown in the vicinity of the Ipatovo mound, vegetation zones might have shifted with changing climate conditions. The valley belongs to the river Kalaus drainage system. All 37 burial mounds of the cemetery, of which mound 22 was the largest and placed slightly beside the others, were excavated by the local heritage organisation 'Nasledie' in 1998-2001. Material was analysed from mounds 17, 22 and 37. Mound 17 was located on the ridge of the watershed. Its diameter was 28 m, its height reached 0.5 m. A total of nine burials and two mound shells were revealed in the barrow. Together with grave 2c, burial 6 was dug into the virgin soil. Grave 6 was probably associated with a low stone shell above. Mound 22 was slightly oval, had a diameter of 38 m and a maximal height of 1.8 m. It comprised three major mound construction phases of which the first was a complex stone construction dating to the Maykop epoch [27]. The sampled

individuals stem from this first mound phase. The first interments in the mound, grave 8 and 8a, were found in a massive stone box covered by a cairn-like stone package. It was surrounded by a large circle of vertical stone slabs, some of which had collapsed to the interior. Graves 9, 15 and 16 were added inside the circle, and some were covered by collapsed stones of the stone circle. The entire construction was then filled with a layer of topsoil. It was covered by a stone shell and an earthen mound-shell (shell 1). The remaining 12 graves date chiefly to the Middle and Late Bronze Age and are associated with the North Caucasian cultural formation (graves 4, 10), the Catacomb cultural formation (graves 7, 12-12) and a late or post-Catacomb horizon (graves 1, 3, 11, 12). Graves 2, 5 and 6 are uncertain in date. The Maykop individuals were part of a paleoanthropological study and investigated with regards to their radiocarbon and stable isotope composition as well as for paleoproteomic analysis[31,67]. The earliest predecessor of the DOM2 horse lineage in the Caucasus-Volga-Don steppe has been retrieved from grave 16[47]. Paired radiocarbon dates on human and animal bones, chiefly herbivores, revealed radiocarbon off-sets for some bones (see comments below), but confirm a mid to late Maykop date for all graves The particularities of inventories and grave construction together with the location of the site in the grass-steppe zone associate these complexes with the 'Steppe Maykop' cultural formation. Mound 37 is situated approximately 50 m northeast of mound 22 at the slope of the watershed. The low mound was built on top of a catacomb grave inside a rectangular ditch-construction. The ditch was partly covered with stones.

In this study, we report new genome-wide data from three individuals from two different barrows. Two individuals were reported in our previous study[29], but are also listed here for context, as these were also included in biological relatedness estimations.

- **AY2001**[29]**,** BZNK-0290/1, kurgan 22, grave 15. *Context information*: grave of a 55-65-year-old female individual in a long-oval pit with entrance shaft, partly covered by stones and associated with a ceramic vessel. Ceramic vessel, snake bones. *Cultural affiliation & dating:* Steppe Maykop, early, OxA-16182: 4698±32 BP (cal. BC 3522-3378, cal. BC 3623-3371), hb; **OxA-16183: 4608±31 BP (cal. BC 3493-3354, cal. BC 3514-3193), ab**; GIN-11811: 4480±50 BP (cal. BC 3335-3095, cal. BC 3361-2970), hb. *Comment:* 4th-5th-degree related to NV3003.
- **AY2003**[29]**,** BZNK-0289/1, kurgan 22, grave 9. *Context information*: The burial of a 1.5-year-old child in an oval burial pit covered by stones and associated with two ceramic vessels, a flint flake and an astragalus. Two ceramic vessels, a flint flake and an astragalus. *Cultural affiliation & dating:* Steppe Maykop, early, **OxA-16201: 4798±33 BP (cal. BC 3638-3531, cal. BC 3641-3525), ab**; OxA-16147: 4726±31 BP (cal. BC 3622-3381, cal. BC 3630-3376), hb. *Comment:* 5-7th degree related to SA6001.
- **AY2004,** BZNK-1002/1, kurgan 22, grave 16. *Context information*: Single inhumation in an oval grave-pit, partly covered by stones of the cromlech, skeleton in crouched position on the left side, head towards northeast. The skeleton was surrounded by a collection of 15 animal bones, among them that of a horse, an ass and the scapula and astragal bones of several sheep.

Between the stones of the mound shell several fragments of ceramic vessels and a stone pestle(?) were uncovered. *Cultural affiliation & dating:* Steppe Maykop, early, **OxA-16185: 4724±31 BP (cal. BC 3621-3381, cal. BC 3630-3376), ab**; OxA-16136: 4720±43 BP (cal. BC 3623-3379, cal. BC 3632-3373), hb. *Comment:* 5-7th degree related to SA6001; one of the horse bones revealed the oldest ancestors of the DOM2 horse lineage.

- **AY2005,** BZNK-1013/1, kurgan 37, grave 1. *Context information*: Well-preserved single inhumation in a catacomb with broad shaft; individual in crouched position on the left side, with the head in southeastern direction. The burial inventory included a complete ceramic vessel, fragments of a second in the fill, a bronze dagger in front of the arms and two whetstones(?) at the feet. *Cultural affiliation & dating:* Lola/post-Catacomb horizon, **MAMS-45944: 3702±21 BP (cal. BC 2137-2039, cal. BC 2196-1986), hb**, OxA-16130: 3676±29 BP (cal. BC 2135-1982, cal. BC 2142-1958), hb; OxA-16131: 3720±30 BP (cal. BC 2196-2039, cal. BC 2204-2027), hb. *Comment*: Paleoproteomic analysis revealed the consumption of dairy products from sheep and goat milk[31].
- **AY2006,** BZNK-1116/1, kurgan 17, grave 6. *Context information*: Single inhumation in supine position with bent up knees in an oval burial pit with a sloping ground, and the head in eastsoutheast direction. The skull and the lower part of the skeleton were covered with ochre. Beside the head a pendant made of boar tusk, a bone pin, a long silex blade and a small copper button were found. *Cultural affiliation & dating*: Steppe Eneolithic, MAMS-51692: ^{14}C date failed due to insufficient collagen preservation, hb.

2.3.3 Bamut

Country: Russian Federation

Region: Sernovodsk district, Chechnya

Coordinates: 43.16350°, 45.197704°

Excavation details: The Bamut burial mound 6 is part of a mound necropolis near the eponymous village in the hilly piedmonts of the Caucasus foreland dating to the Early and Middle Bronze Age. The central grave 1 in mound 6 dates to the Eneolithic period[22]. The barrow was 1.55 m high with a diameter of 21 m. At a depth of 1.6 m a grave-pit with a size of 0.8×0.55 m and a depth of 0.65 m was excavated.

- **BMT001,** kurgan 6, grave 1. *Context information*: Single inhumation lying on the back with bent up legs. The whole skeleton was packed in a thick layer of red ochre. A flint plate with retouch along the edges was found near the right forearm (arrowhead?), and paste beads were scattered all over the grave. *Cultural affiliation & dating:* Steppe Eneolithic, MAMS-55777: 5221±23 BP (cal. BC 4045-3986, cal. BC 4159-3969), hb.

2.3.4 Boykoponura

Country: Russian Federation

Region: Kalininsky district, Krasnodar region

Coordinates: 45.37964°, 38.76285°

Excavation details: Excavation 'Research Centre for Cultural Heritage Preservation' 2017, Saratov, licence №2017-1160 (A. G. Sheremetev).

Site information: Near the village Boykoponura in the Kuban plain north of the eponymous river several barrow groups with up to 20 mounds are located. The studied material originates from kurgans 2 and 3 of a group with three mounds, situated about 1.25 km north of the village and east of the small nearby river Ponura[68]. All kurgans were heavily ploughed-up/over with only a few remains of the embankments remaining. Kurgan 2 is central to a line that runs in a south-east to north-west direction. Determining its original size is problematic, visible were mound shells with a diameter of approx. 75 m with a remaining height of 1.5 m. As a gas pipeline runs through the south-western part of the mound, this area was not excavated. The three mound-shells of kurgan 2 encompassed nine burials. Graves 3 and 8 date to the Early Bronze Age, graves 4, 5 and 9 date to the Middle Bronze Age, and grave 7 dates to the Late Bronze Age, while graves 1 and 2 are assigned to the Sarmatian period. Kurgan 3 was circular with a visible diameter of ca. 55 m and a preserved at a height of 0.9 m. A first mound-shell was built for Early Bronze Age grave 6, graves 12 and 13 are secondary and covered by a second mound-shell. Graves 4, 7 and 14 date to the Middle Bronze Age, graves 2, 3, 9 and 11 are Sarmatian, and grave 1 was interred during the Middle Ages.

The archaeological material is unpublished.

In total we sampled 17 individuals from this site (Supplementary Table 2), of which nine are reported here and one was excluded due to an Iron Age date. The remaining eight samples did not contain sufficiently high DNA yield/quality and were thus not further pursued after initial SG screening.

- **BOK003,** BZNK-1196/1, kurgan 3, grave 6, individual 3. *Context information*: This is the initial grave of the mound, which contained the remains of an adult with two infants on either side in a rectangular burial pit, with the heads of all individuals directed to the east. The adult was placed in a slightly crouched position with the upper body on the back. The children were placed on their back, the northern individual was ca. 2-4 years old, the southern child ca. 6-7 years of age. Below the deceased organic matter and spots of red ochre were found. Four bone pins and a shell were documented at the shoulder of the adult, 11-12 lumps of red ochre coloured red with ochre beside the northern child and a ceramic vessel at the head of the southern child. *Cultural affiliation & dating:* Yamnaya, MAMS-55778: 4213±20 BP (cal. BC 2889-2775, cal. BC 2895-2701), hb.
- **BOK004,** BZNK-1197/1, kurgan 2, grave 3. *Context information*: Single inhumation of an adult in a rectangular burial pit. The deceased was placed in a right-side, crouched position, with the head in the south-east. No grave goods were found, but white and grey organic matter was found below the skeleton, and red ochre in front of the deceased's body. *Cultural affiliation & dating:* Early Bronze Age.

- **BOK009,** BZNK-1202/1, kurgan 2, grave 7. *Context information*: Remains of an inhumation, destroyed in antiquity, with only a few bones preserved. Burial gifts include a bronze dagger or knife, one bronze pricker(?). *Cultural affiliation & dating:* Late Bronze Age, Steppe, MAMS-55779: 2908±19 BP (cal. BC 1153-1048, cal. BC 1200-1014), hb.
- **BOK010,** BZNK-1203/1, kurgan 2, grave 8. *Context information*: The grave is initial for the second mound shell in mound 2, and contained a single inhumation in a deep, rectangle burial pit covered with wooden planks. The individual was placed in crouched position, with the head directed to north-west. No inventory was found, but remains of a caprine (head, legs) were placed on top of the ceiling, and red ochre and organic matter were documented below the skeleton. *Cultural affiliation & dating:* Early Bronze Age (Novotitarovskaya?).
- **BOK011,** BZNK-1204/1, kurgan 2, grave 9. *Context information*: With a few bones of the upper body, only remains of an inhumation were preserved. The individual was likely placed in a crouched position, with the head to the north. No inventory. *Cultural affiliation & dating:* Catacomb, late/post-Catacomb Kuban group, MAMS-55780: 3754±20 BP (cal. BC 2202-2139, cal. BC 2279-2045), hb.
- **BOK014,** BZNK-1207/1, kurgan 3, grave 10. *Context information*: Single inhumation of a child in supine position in an oval burial pit. The leg bones were destroyed, but a grinding stone near the head and a spot of red ochre near the right arm were documented. *Cultural affiliation & dating:* Catacomb, late.
- **BOK015,** BZNK-1208/1, kurgan 3, grave 12. *Context information*: The grave is the initial burial of the second mound-shell of mound 3, and contains a poorly preserved single inhumation in a rectangular burial pit. The individual was placed in a right-side, flexed position with the head in the north. A mat coloured with dark red ochre was documented below the skeleton, and a head of a caprine at. the human head. *Cultural affiliation & dating:* Bronze Age.
- **BOK016,** BZNK-1209/1, kurgan 3, grave 13. *Context information*: Single inhumation in a rectangular burial pit. The individual was placed on the right side, with the head directed to the south-west. Organic matter was documented below the skeleton and red ochre at the knees. No inventory. *Cultural affiliation & dating:* Bronze Age.
- **BOK017,** BZNK-1210/1, kurgan 3, grave 14. *Context information*: Catacomb grave with a single inhumation in a deep, T-shaped catacomb with a broad shaft. The individual was placed in a crouched position on the right side, with the head tilted forward to the chest. The deceased's feet were stained with red ochre and organic matter was documented below the skeleton. A fragmented ceramic vessel was found at the entrance of the shaft, and a skull of a caprine placed on the leg bones in the southeastern corner. *Cultural affiliation & dating:* Catacomb, early, MAMS-55781: 4209±26 BP (cal. BC 2889-2707, cal. BC 2898-2679), hb.

2.3.5 Ergeninskiy 1

Country: Russian Federation

Region: Ketchenerovskiy district, Kalmykia

Coordinates: 47.11452°, 44.49564°

Excavation details: Excavation Kalmyk Scientific Centre of the Russian Academy of Sciences (M. Ochir) and Eurasia-Department DAI Berlin (K. Malek-Custodis), 2008-2008, licence (M. Ochir).

Site information:

The Ergeninskiy group of burial mounds is located on the Ergeninskiy plateau in the Caspian plain, about 2-2.5 km north of the village and a small river Keke Buluk. The mounds form an elongated group or line, running north to south perpendicular to the river. Soviet archaeologist Valentin P. Shilov conducted excavations of 12 of the Ergeninskiy kurgans in 1981–1986[69]. Between 2006 and 2008, a joint expedition of the Kalmyk Scientific Centre of the Russian Academy of Sciences and the Eurasian-Department of the German Archaeological Institute excavated barrows 13 and 14[70]. With a few exceptions, all currently excavated mounds belong to the East Manych Catacomb culture and were built in this epoch. That is an unusual feature, as the Catacomb culture communities usually reused mounds built by the preceding Yamnaya culture. The kurgans vary in size and number of graves. The barrows partly contain sacrificial sites related to graves. They are made up of bovine skulls, lower extremities and sometimes incense burning bowls.

Kurgan 2 had a diameter of 21 m and was 0.5 m high, and contained one interment. Kurgan 5 had a diameter of 50 m in diameter, was 1.85 m high, and contained nine interments. Kurgan 6 had a diameter of 59.5 m and was 5.45 m high, and contained five interments. Lastly, kurgan 13 had a diameter of 49 m and was 3.34 m high, and contained nine interments.

In total we sampled and screened samples from 12 individuals from this site (Supplementary Table 2), of which 7 yielded sufficient ancient human DNA for further analyses after 1240k SNP capture (Supplementary Table 1).

- **ERG001,** BZNK-1082/1, kurgan 2, grave 1. *Context information*: Single inhumation in a catacomb grave with entrance pit of skeleton in left-side flexed position, with the head towards the north. A clay turnip-shaped vessel was found in front of the skull, a bronze awl, bronze knife and a mortar in the north west corner, another bronze knife in front of the chest, and a grinding plate in the south east corner. *Cultural affiliation & dating:* Catacomb culture (East Manych variant).
- **ERG005,** BZNK-1231/1, kurgan 4, grave 3. *Context information*: Double inhumation of an adult and a child in a catacomb grave with entrance pit. Both skeletons were found in a left-side flexed position, with the heads in the south. A clay vessel was placed in front of the skulls and in the northwest corner. Clay sherds were documented behind the legs. A mandible and shoulder fragment of a cow and an incense bowl were found in the entrance of the pit. *Cultural affiliation & dating:* Catacomb culture (East Manych variant).

- **ERG007**, BZNK-1233/1, kurgan 5, grave 6. *Context information*: Single inhumation in a catacomb grave with entrance pit. The skeleton was found in a left-side, flexed position, with the head in the east. A clay turnip-shaped vessel was found in front of the skull, and in front of the chest were wooden fragments. In front of the lower part of the body fragments of an organic mat. *Cultural affiliation & dating*: Catacomb culture (East Manych variant).
- **ERG008,** BZNK-1234/1, kurgan 6, grave 2. *Context information*: Single inhumation in left-side position in a cross shaped pit covered with several layers of wooden planks and organic mats. Depressions filled with ash were found in every corner. The skeleton was covered with textile fragments. Numerous grave goods like a bronze knife on a wooden plate, animal bones, a clay turnip-shaped vessel, a bronze hook, two bronze axes, another bronze knife, bronze awl, a bronze chisel, fife stones, and pair of arrow smoothers represent the burial inventory. Small bronze rings were situated above and under the pelvis, and carnelian and glass paste beads around the neck and chest. *Cultural affiliation & dating*: Catacomb culture (East Manych variant).
- **ERG011,** BZNK-1237/1, kurgan 13, grave 3, individual 2. *Context information*: Single inhumation of an unborn child (a 7-month-old foetus) in a catacomb grave with entrance pit and fragments of a wooden wagon[70]. The individual was buried in a left-side flexed position on an organic mat, with the head in the south. A clay turnip-shaped vessel was found in front of the skull, a wooden tray, fragments of wooden vessel and a bronze knife in front of the body, while beads were found around the skull and the chest. *Cultural affiliation & dating:* Catacomb culture, Hd-29181/ETH-39114: 3870±25 BP (cal. BC 2458-22204, cal. BC 2461-2210), unidentified organic matter (plant remains?) from below individual 1.
- **ERG012,** BZNK-1237/1, kurgan 13, grave 5. *Context information*: Single inhumation of a child (up to 1 year) in a catacomb grave with entrance pit. The skeleton was disturbed, but below it remains of an organic mat were found. Burial gifts included three clay vessels, three stones, a complete mollusc and several fragments of others, 20 astragals of sheep, a tubular bone, and two mouthpieces made from bone. Between the bones one bronze glass paste bead was discovered. *Cultural affiliation & dating:* Catacomb, late, KIA-30257: 3853±29 BP (cal. BC 2437-2209, cal. BC 2458-2204), hb.

2.3.6 Essentukskiy 1, Kurgan

Country: Russian Federation

Region: Predgorny district, Stavropol region

Coordinates: 44.06589°, 42.90257°

Excavation details: Excavation 'Nasledie' 2017, Stavropol, licence №2017 (A. A. Kalmykov)

Site information: The huge barrow was located at the north-eastern outskirts of the city Essentukiy in a developing area. Its location marks the border between the steppe zone and the foothills of the North

Caucasus. The mound was built at a gentle slope of the second or third river terrace at the left bank of the Podkumok river. This slope is the watershed between Pokumok and Kuma rivers. Today, the barrow is solitary, but barrows similar in size are still preserved nearby. Together with evidence from older aerial images, this suggests a line of at least 4 large mounds running north-west to south-east, some of which were originally 8-9 m high.

The mound of Essentukskiy 1 (5.5-6.0 m high, 60 m wide) was built over a collective burial pit (grave 9), in a short period during the early Maykop culture. The burial pit was repeatedly opened and cleared out at different times. The earthen shells of the mound consisted of six consecutive earth and three stone structures (two stone packages and a cromlech). Their construction followed a similar plan, using specific techniques to construct the shells with rammed earth and clay [71]. The construction was accompanied by numerous ritual activities. Shortly after the construction of the mound was completed, a child burial with a round-bottom vessel of the Yamnaya culture was made near the outer boundary, but no other Bronze Age burials were interred into the mound. In the south-eastern part of the construction a small group of four burials from the initial period of the pre-Scythian Early Iron Age (Koban culture?) were dug into the mound. The last stage is associated with the Sarmatian period, during which three burials were interred. The archaeological material is still unpublished.

In total we sampled and screened samples from seven individuals from this site (Supplementary Table 2), of which five yielded sufficient ancient human DNA for further analyses after 1240k SNP capture (Supplementary Table 1):

- **ESY001_ESY002,** BZNK-1073/1, grave 9, individual 4. *Context information*: Multiple burial in a rectangular burial pit that was oriented west-east (5 x 3 m) and covered with wooden beams with the remains of at least five individuals. The skeletal remains were displaced by repeated intrusions after the burial, the minimum number of individuals was determined anthropological identification (N.Y. Berezina). Fragments of about half a dozen ceramic vessels were found. with some ceramic fragments also outside the chamber. Gold items were documented in the original reports, with only one gold bead remaining. *Cultural affiliation & dating:* Maykop, early, MAMS-38067: 4807±34 BP (cal. BC 3640-3531, cal. BC 3644-3527), plant remains (unidentified).
- **ESY003_ESY004,** BZNK-1073/4-5, grave 9, individual 3. *Context information and dating*: see above, *Cultural affiliation & dating:* Maykop, early.
- **ESY005,** BZNK-1073/7, grave 9, individual 5(?). *Context information and dating*: see above, *Cultural affiliation & dating:* Maykop, early. *Comment:* 4-5th degree related to individuals MK5001 & MK5004.
- **ESY006_ESY007,** BZNK-1111/1; 3, grave 4. *Context information*: The burial was located in the south-eastern sector of the mound. A grave structure was not traced, the skeleton was found on the stones of the second, later stone package of the barrow. The 18-24-year-old male individual was positioned in a strongly flexed position on the left side, with the head in the

north-east and arms bent in front of the chest. No burial equipment. *Cultural affiliation & dating:* Pre-Scythian Steppe (LBA), MAMS-51683: 2924±20 BP (cal. BC 1194-1055, cal. BC 1211-1021), hb.

- **ESY009_ESY010,** BZNK-1112/1-2, grave 5. *Context information*: The burial was located in the south-eastern sector of the mound, outside the cromlech and the stone packages. No grave structure was traced. The 20-25-year-old male individual was positioned in a left-side crouched position, with the head directed to north-north-east. The left hand was stretched out to the knees, the right hand was bent in front of the chest. A small ceramic goblet was placed in the grave as a funerary offering. *Cultural affiliation & dating:* Pre-Scythian Steppe (LBA, Koban culture?), MAMS-58128: 2866±24 BP (cal. BC 1109-1001, cal. BC 1122-932), hb.

2.3.7 Gatyn-Kale

Country: Russian Federation

Region: Shatoy district, Chechnya

Coordinates: 42.85931°, 45.71865°

Excavation details: Excavation North Caucasian Expedition RAS, Argun branch, Moscow, 1956-1959 (V. I. Markovin)

Site information: The archaeological site of Gatyn-Kala (Gatyn-Kale) near the present-day village Aslanbek-Sheripovo, is a cemetery with Bronze Age graves in a mid-altitude valley at a slope below a steep, forested hillside in a side valley of the Sharo-Argun gorge. At the cemetery 36 stone masonry lined crypts were excavated between 1956 and 1959 under the direction of V. A. Markovin[72]. Most graves were organised parallel with a north-west orientation in lines with some a slightly different orientation. The graves contained multiple interments of up to ten or more individuals and burial gifts including metal ornaments and ceramic vessels. Over 150 individuals have been excavated, yet only a small collection of the anthropological material was kept in the Museum of Anthropology at the Moscow Lomonosov State University[73].

The individual involved in this study is one of two, whose skulls have been kept from collective grave 14. We sampled both, but only yielded sufficient ancient human DNA for further analyses from one individual.

- **GTK001,** Research Institute and Museum of Anthropology of Lomonosov Moscow State University (RIMA) 10896, Gatyn-Kale, collective burial 14. *Context information*: Collective grave in a crypt with stone masonry walls. The grave is in a row of five stone box or crypt graves, and was cut by grave 13. The crypt held three layers of inhumations of at least ten individuals whose bones were in disorder. The overall inventory comprises a bronze pin, temple rings, cardium shells, a collection of 35 astragals and more such items in the grave fill, such as animal bone tools and several ceramic large vessels. The skull studied is that of a child. *Cultural affiliation & dating:* Ginchi culture.

2.3.8 Ginchi

Country: Russian Federation

Region: Shamilskiy district, Dagestan

Coordinates: 42.37700°, 46.60584°

Excavation details: Excavation Mountain Expedition of the Dagestan branch AN SSSR, 1956-1960 (M. Gadzhiev)

Site information: The cemetery of Ginchi is located about 3 km east of the village Tidib in the mountainous part of Dagestan. The location is a flat promontory on the right bank of the Gichinoor creek at the foot of an escarpment. The creek had eroded parts of the site prior to excavation. In 1956-1960 15 crypts, two graves of children, and 15 burials in ceramic vessels were excavated in an area of 350 m^2. The materials were published in a monograph by M. Gadzhiev in 1963[74]. Most crypts have a roundish or square ground plan and are built with stone masonry. All graves contained numerous individuals in several layers on top of each other, together with burial gifts, mainly pottery. Some of the skeletons had been moved to one side, others lay intact side by side. The necropolis is associated with the eponymous Ginchi culture of the late Middle Bronze Age that is a represent settled mountain agricultural communities. Genetic analyses were carried on several skulls that are kept in the Museum of Anthropology, Moscow Lomonosov State University[73]. These originate from crypts 2 and 3.

In total we sampled and screened skulls from nine individuals from this site, of which seven were suitable for 1240k SNP capture, after which six yielded sufficient ancient human DNA for further analyses (Supplementary Table 1).

- **GIN001,** RIMA 11550, crypt 2. *Context information*: Collective burial with several layers of skeletons in a stone crypt built of drystone walls in the form of a large circle of 3.5 to 3.7 m diameter. The complex was disturbed in its upper parts by later agricultural pits. In the upper layer 12 skeletons were recorded, placed in strongly crouched positions together with metal ornaments and many ceramic vessels, among them large storage vessels. Individuals 13 to 31 were found in the middle layers, similarly equipped with metal ornaments and tools as well as ceramic vessels and in crouched position. Individuals 32 to 34 are from r the lower layer, similarly placed as the others. The layers were divided by strata of stone and an internal compartmentation was recorded as well. In the Moscow collection of RIMA curates at least seven skulls associated with this crypt, three of which are included in this study. A full concordance of the museum's collection and the original descriptions in Gadzhiev 1969, however, is not possible. In addition to the skeletons, the crypt contained a wealth of metal ornaments, tools and ceramic vessels. *Cultural affiliation & dating:* Ginchi culture.
- **GIN002,** RIMA 11551, crypt 2. *Context information*: see above. *Cultural affiliation & dating:* Ginchi, MAMS-42348: 3509±19 BP (cal. BC 1884-1775, cal. BC 1893-1750), hb.
- **GIN003,** RIMA 11552, crypt 2. *Context information*: see above. *Cultural affiliation & dating:* Ginchi, MAMS-45945: 3756±24 BP (cal. BC 2269-2136, cal. BC 2283-2042), hb.

- **GIN005,** RIMA-11556, crypt 3. *Context information*: Collective burial with several layers of skeletons in a stone crypt similar to crypt 2. This crypt had the form of an apsis and dimensions of ca. 1.85 x 1.65 m, and was still covered by stone slabs prior to excavation. In crypt 3 16 individuals were documented in four layers that were divided each by strata of earth. For the upper layer two individuals were recorded, in layer 2 four skeletons were preserved in anatomical order, in layer 3 the remains of eight skeletons were scattered, and in layer 4 another two skeletons were found. The individuals were accompanied by metal ornaments, stone beads, and a rich inventory of ceramic vessels and spindle whorls, one possibly a wheel model. Three skulls from this complex are stored in the Moscow collection of RIMA, two of which are included in this study. A full concordance of the museum's collection and the original description in Gadzhiev 1969 is not possible. *Cultural affiliation & dating:* Ginchi, MAMS-42349: 3351±20 BP (cal. BC 1684-1547, cal. BC 1733-1542), hb.
- **GIN006,** RIMA 11556, undetermined crypt. *Context information*: Skull from the crypts excavated in 1959 to 1960 in the collection of RIMA Moscow. The exact number of the crypt is not indicated, the skull likely also stems crypt 2 or 3. *Cultural affiliation & dating:* Ginchi, MAMS-45946: 3758±24 BP (cal. BC 2270-2137, cal. BC 2284-2043), hb.
- **GIN007,** RIMA 11558, crypt 3. *Context information*: see before. *Cultural affiliation & dating:* Ginchi, MAMS-42364: 3345±20 BP (cal. BC 1670-1544, cal. BC 1729-1538), hb.
- **GIN008,** RIMA 11560, undetermined crypt. *Context information*: Skull from the crypts excavated in 1959 to 1960 in the collection of RIMA Moscow. The exact number of the crypt is not indicated, the skull likely also stems from crypt 2 or 3. Cultural affiliation & *dating*: Ginchi, MAMS-45947: 3851±23 BP (cal. BC 2402-2210, cal. BC 2455-2205), hb.

2.3.9 Ilinskiy 1

Country: Russian Federation

Region: Izobilniy district, Stavropol region

Coordinates: 45.56338°, 41.82260°

Excavation details: Excavation 'Nasledie' 1998, Stavropol, licence №1998-550 (Y. B. Berezin)

Site information: The burial mound cemetery was situated on the right bank terrace of the river Egorlyk, 3.9 km south-south-west of the village Ilinskiy. Burial mounds 1, 2 and 4 were located on a meadow prior to excavation, whereas barrows 3, and 5-7 were located on arable land and thus affected by ploughing. The cemetery consisted of two separate barrow clusters, a southern and a northern one, ca. 0.4-0.6 km apart. Before the excavation, mound 1, the largest of the group, had a height of 2.0-1.4 m and a diameter of 50 m. At least 14 burials can be associated with two building phases. The founding grave 5 in the centre dates to the beginning of the Middle Bronze Age and is one of the few complexes with a post-Maykop shaft hole axe. The other graves in the mounds belong to various Bronze Age groups, such as Catacomb and post-Catacomb cultures.

- **ILL001,** BZNK-1026/1, kurgan 1, grave 6. *Context information*: Single inhumation without recognizable traces of a grave-pit of a poorly preserved skeleton in crouched position on the left side, with the head towards southeast. A bronze knife was documented in the right hand, and a pendant in the corner between the left forearm and the left femur. *Cultural affiliation & dating:* Lola/post-Catacomb horizon, MAMS-42350: 3751±18 BP (cal. BC 2201-2138, cal. BC 2276-2044), hb. *Comment*: Paleoproteomic analysis proved the consumption of dairy products from sheep and goat milk[31].

2.3.10 Ipatovo 3

Country: Russian Federation

Region: Ipatovo district, Stavropol region

Coordinates: 45.67661°, 42.91509°

Excavation details: Excavation 'Nasledie', Stavropol 1998-1999, licence №1998-177 (A. B. Belinskiy)

Site information: The big mound of Ipatovo (Ipatovo 3, mound 2) was part of a series of linear alignments of mounds that run west-east crossing the system of the Kalaus river. Today the slightly hilly zone around the site is part of the North Caucasian herb and grass-steppe zone, while the Kalaus valley itself is part of the desert-steppe. However, the vegetation likely shifted between desert and grass steppe in prehistory. During the construction of the mound, the climate was generally warmer and more humid than it is today, and the landscape was more forested. Mound 2 was the largest in a group of at least eleven mounds visible from aerial images. It was excavated due to construction work and yielded a total number of 195 burials in 11 construction phases. 151 of the interments were part of a Nogay cemetery dating to the 18th century AD. One grave held the burial of a splendidly furnished Sarmatian woman[75]. The mound construction and 34 graves were assigned to a Bronze Age date and published in a monograph[76]. Several of the Catacomb-epoch interments were accompanied by wooden wagons. The first three mound shells are associated with graves that belong to an Eneolithic or Steppe-Maykop tradition. Grave 187 is the founding grave of the entire mound. It was attributed to a local Eneolithic tradition in the monograph. However, after re-evaluation of the data, we would now rather group it with Steppe Maykop complexes. The grave of this site was part of a study including in-depth genetic, palaeoproteomic and isotope analysis[31,77], and was shown to have been infected by a human adapted version of *Salmonella enterica*[78].

From Ipatovo 3 we collected samples from 10 individuals during several years. Data from one individual was reported earlier[29], and one was excluded due to a later Iron Age date. Four individuals yielded sufficient ancient human DNA for further analyses and are reported below, whereas the remaining five individuals did not have sufficiently high DNA yield/quality and were thus not further pursued after initial SG screening.

- **IV3002,** BZNK-0293/1, kurgan 2, grave 187. *Context information*: The rectangular-oval pit covered by stone slabs is the central founding grave of the mound. Only scattered bones are left

from the single inhumation, and two bones of an unidentified bird, bigger in size than a goose, were documented. *Cultural affiliation & dating:* Steppe Maykop, late/local Eneolithic, GIN-10297: 4630±50 BP (cal. BC 3511-3358, cal. BC 3617-3124), hb; **OxA-16187: 4865±33 BP (cal. BC 3702-3544, cal. BC 3711-3531), ab (bird?[79])**.

- **IV3005,** BZNK-1034/1, kurgan 2, grave 182. *Context information*: Single inhumation in round to trapezoid grave-pit with walls covered with reed mats. The grave was covered with stone constructions and included a poorly preserved skeleton in left-side crouched position, with the head in the south. Jet beads, charcoal and stone beads were found on the chest. *Cultural affiliation & dating:* Steppe Maykop, late, **MAMS-45948: 4721±23 BP (cal. BC 3605-3380, cal. BC 3626-3377), hb**; GIN-10952: 4390±70 BP (cal. BC 3264-2908, cal. BC 3335-2894), hb.
- **IV3008,** BZNK-1068/1, kurgan 2, grave 162. *Context information*: Single inhumation in a catacomb grave with entrance pit; the skeleton was found on its left-side in strongly flexed position with the head in the north, below chalky matter was found. Charcoal remains and a cranium of a young sheep were documented. In the grave fill and around the human skeleton several skeletons of snakes were excavated. *Cultural affiliation & dating:* Catacomb, early, **OxA-16137: 4035±30 BP (cal. BC 2880-2701, cal. BC 2890-2672), ab (ovis/capra)**; OxA-16138: 4185±30 BP (cal. BC 2880-2701, cal. BC 2889-2636), ab; MAMS-45950: 4192±26 BP (cal. BC 2882-2704, cal. BC 2890-2672), hb.
- **IV3010,** BZNK-1032/1, kurgan 2, grave 183. *Context information*: Single inhumation in a rectangular stepped grave-pit, covered by stones. Skeleton in left-side, crouched position with the head in the south on an organic pillow/mat? No inventory was found. *Cultural affiliation & dating:* Steppe Maykop, late, MAMS-45951: 4552±25 BP (cal. BC 3366-3126, cal. BC 3371-3103), hb.

2.3.11 Kaluzhny 1

Country: Russian Federation

Region: Shpakovsky district, Stavropol

Coordinates: 44.84851°, 42.21984°

Excavation details: Excavation 'Nasledie', Stavropol 2008, licence №2008-1310 (S. V. Lyakhov)

Site information: The kurgan cemetery Kaluzhny 1 is located 3 km southeast of the village Kaluzhny. It was investigated during rescue excavations in 2008. The cemetery included three barrows. With 1.44m in height and a diameter of 28 m prior to excavation, mound 1 was the largest of the group. It was built in two phases and covered 23 interments in total. The majority of graves date to the post-Catacomb Lola culture (n = 11), two to the Middle Bronze Age, one is attributed to the Catacomb culture and four date to the Sarmatian period. Three graves could not be assigned. The archaeological material is unpublished.

- **KLZ001,** BZNK-1029/1, kurgan 1, grave 8. *Context information*: Single inhumation without recognizable traces of a grave-pit set into the mound shell. The skeleton was found in the left-side, crouched position with the head in the northeast. Yellow organic remains were documented on the lower part of the body, brown organic remains under the skeleton, white organic remains to the east of the left shoulder and shinbone. In addition, 41 coloured faience beads, 12 pearl pendants, two animal teeth pendants, charcoal, and ochre around the left hand and shoulder were found. *Cultural affiliation & dating:* Lola/post-Catacomb horizon, MAMS-45952: 3793±25 BP (cal. BCE 2284-2149, cal. BCE 2297-2139), hb.

2.3.12 Kammenomostskoe

Country: Russian Federation

Region: Zolsky district, Republic of Kabardino Balkaria

Coordinates: 43.73659°, 43.04875°

Excavation details: Expedition P.G. Akritas 1954

Site information: Necropolis with flat graves of the Early Iron Age Koban culture[80]. The site is situated in the northern foothills of the Greater Caucasus in the centre of the modern settlement of Kamennomostskoe, atop the confluence of the rivers Kič-Malka and Malka. The cemetery is located on a flat slope of a promontory between the two rivers and is partly covered by gardens and buildings today. The site with an area of 420 m x 90-180 m has been known since the 19th century AD and has been sporadically investigated by several archaeologists from 1914 to 2009. Burial assemblages in the Kamennomostskoe graves included lavish inventories with bronze and iron objects as well as pottery. The analysed individual was sampled from the materials kept in the Museum of Anthropology, Moscow Lomonosov State University. From its inventory ID 1954/3 it should originate from two stone boxes excavated by P.G. Akritas in the southern part of the cemetery. The radiocarbon dating, however, attributes this sample to the MBA. It is possible that this results from a mix-up of remains in the museum, as no MBA contexts are known from the site. We nonetheless kept this sample for its biological information.

- **KMK001**, no BZNK-ID, no *Context information. Dating:* MAMS-51691: 4142±20 BP (cal. BC 2864-2636, cal. BC 2871-2627), hb.

2.3.13 Khutor Belyy

Country: Russian Federation

Region: Temryuk District, Krasnodar region

Coordinates: 45.15869°, 37.23832°

Excavation details: Excavation 'Research Institute of Archaeology and Ancient History of the North Caucasus', Stavropol 2017, Stavropol, licence №2017-823 (S. V. Lyakhov).

Site information: The mound is a singular monument southwest of the hamlet Belyy located on the Taman peninsula. It is situated on a watershed between the Vityazevsky and Starotitarovsky estuaries at the southern slopes of the mud volcano Kamyshevataya. Seven graves and a stone circle were excavated below a mound with a diameter of 45-50 m and a height of 4.63 to 4.78 m. It was built with two mound shells. The initial grave 7 dates to the Eneolithic period, one likely to the Early Bronze Age, four to the Middle Bronze Age, and one dates to the Early Iron Age. The archaeological material is still unpublished.

In total we sampled and screened five individuals from this site (Supplementary Table 2), of which three were suitable for 1240k SNP capture, after which only one yielded sufficient ancient human DNA for further analyses (Supplementary Table 1):

- **KHB001** BZNK-1079/1, kurgan 1, grave 7, individual 8. *Context information*: Collective burial in an irregular, large pit. At least ten skeletons had been placed irregularly in the pit in several layers. The upper parts reveal traces of fire, while the lower skeletons were packed into a thick layer of red ochre. All heads were located at the eastern side of the pit, while the lower parts and the legs were scattered in the west. Skull 8 was placed at the eastern wall of the pit on top the upper layer of the skeletons. No inventory was found. *Cultural affiliation & dating:* Steppe Eneolithic, MAMS-40112: 5387±25 BP (cal. BC 4326-4176, cal. BC 4335-4066), hb (unidentified individual).
- **KHB002,** BZNK-1080/1, kurgan 1, grave 7, individual 5. *Context information*: See above, skull 2 was placed in the centre of the pit on top of the upper layer of the skeletons.
- **KHB003,** BZNK-1081/1, kurgan 1, grave 7, individual 2. *Context information*: See above, skull 2 was placed in the eastern part of the pit on top of the upper layer of the skeletons. MAMS-51700: 5350±17 BP (cal. BC 4310-4070, cal. BC 4318-4057), hb (Ind. 2).

2.3.14 Komsomolec 1 - Marfa

Country: Russian Federation

Region: Kirov district, Stavropol region

Coordinates: 43.97728°, 43.51637°

Excavation details: Excavation 'Nasledie', Stavropol and Eurasia-Department DAI, Berlin 2013-2015, licence №2013-114, №2014-318, №2015-1036 (A. B. Belinskiy)

Site information: The mound Komsomolec 1- Marfa was a single standing mound on the right river terrace of the Zolka creek, located 7.2 km west of the north-western outskirts of the city of Novopavlovsk. The area is located within the piedmont steppe environment today but was densely forested until the 19th century AD. The entire edge of the terrace was formerly covered with barrows, which are lost to modern ploughing. Beside the mound a circular ditch enclosure is visible on aerial and satellite images, as well as from images of a magnetometry survey conducted in 2009 and 2011. At the time of excavations, the oval shaped mound’s height was 8.5 m and a diameter of 62 m along the west-

east axis was 62 m, and 52 m along north-south axis. The mound was built in several stages and 60 graves in total have been excavated in the mound (Extended Data Fig. 8b). Most of these date to the North Caucasian culture and a variant of the Catacomb culture in the piedmont area. However, the initial mound dates to the later Maykop epoch, although the central grave was destroyed. Maykop or transitional graves dated by radiocarbon dates are grave 9, 20, 23 and 43. At least three graves date to premodern times. Beside the burials a ditch with horse skeletons was opened, probably dating to the Sarmatian period. Additionally, a ritual area with human skeletons was discovered beside the mound, from which a postcranial skeleton dates to the Early Iron Age, and two separate skulls are mediaeval in date. The archaeological material is prepared for publication.

We sampled and screened a total of 42 individuals from this site (Supplementary Table 2), of which 14 were suitable for 1240k SNP capture, after which 12 yielded sufficient ancient human DNA for further analyses (Supplementary Table 1):

- **KMM001,** BZNK-0932/1, Komsomolec 1-Marfa, grave 22. *Context information*: Single inhumation in a grave-pit without contours, containing only single bones and a skull fragment, and no inventory. *Cultural affiliation & dating:* Middle Bronze Age, MAMS-50495: 4185±23 BP (cal. BC 2878-2703, cal. BC 2885-2672), hb.
- **KMM002,** BZNK-0933/1, Komsomolec 1-Marfa, grave 23, individual 1. *Context information*: Double inhumation in a rectangular pit with a wooden coffin. Both skeletons were found in supine position with the head in the north. Spots of ochre were documented on the legs of the individual 1, as well as a vessel each at the head of both individuals. *Cultural affiliation & dating:* Northcaucasian culture, MAMS-45953: 4168±27 BP (cal. BC 2875-2696, cal. BC 2882-2633), hb.
- **KMM003,** BZNK-0934/1, Komsomolec 1-Marfa, grave 23, individual 2. *Context information*: See above. *Cultural affiliation & dating:* Northcaucasian culture.
- **KMM008,** BZNK-0939/1, Komsomolec 1-Marfa, grave 28. *Context information*: Single inhumation in a catacomb of a child in right-side crouched position and with the head in the south-east.Below the individual a light brown organic bedding was documented, but no inventory was found. *Cultural affiliation & dating:* Maykop, late, **MAMS-50498: 4505±25 BP (cal. BC 3339-3105, cal. BC 3349-3105), hb**; MAMS-45955: 4476±26 BP (cal. BC 3328-3097, cal. BC 3339-3031), hb.
- **KMM010,** BZNK-0941/1, Komsomolec 1-Marfa, grave 30. *Context information*: Single inhumation in a square pit with a wooden coffin of an 0-3-month-old infant. in supine position with the head in the north-east. A vessel was found at the foot. *Cultural affiliation & dating:* Northcaucasian culture, MAMS-45956: 4163±26 BP (cal. BC 2873-2676, cal. BC 2880-2632), hb.
- **KMM015,** BZNK-0945A/1, Komsomolec 1-Marfa, grave 35. *Context information*: Single inhumation in a catacomb in supine position with head in the south. Below the individual a

square mat of brown organic matter was found and spots of ochre at the lower legs. Cattle bones were documented outside the grave-pit at the step of the entrance shaft. *Cultural affiliation & dating:* Catacomb, early, MAMS-45957: 4079±26 BP (cal. BC 2835-2504, cal. BC 2850-2495), hb.

- **KMM022,** BZNK-0951/1, Komsomolec 1-Marfa, grave 41. *Context information*: Destroyed inhumation in a square stepped pit with wooden cover. The deceased was likely placed in supine position. Apart from some spots of ochre on the ground, no inventory was found. *Cultural affiliation & dating:* Middle Bronze Age, MAMS-50507: 4150±19 BP (cal. BC 2866-2671, cal. BC 2873-2631), hb.
- **KMM023,** BZNK-0952/1, Komsomolec 1-Marfa, grave 43. *Context information*: Single inhumation in a grave-pit without contours in right-side crouched position with the head in the east. A shoulder blade and long bones of a caprine were found. *Cultural affiliation & dating:* Maykop, late, MAMS-45958: 4523±27 BP (cal. BC 3352-3109, cal. BC 3361-3102), hb.
- **KMM028,** BZNK-1088/1, Komsomolec 1-Marfa, grave 42. *Context information*: Single inhumation in a roundish pit in left-side crouched position with the head in the east. Organic bedding was found underneath the skeleton, as well as fragments of the skull between the arms and legs, and small beads of glass or limestone in the thorax area. A second skull was probably placed as an offering into the arms of this individual. *Cultural affiliation & dating:* post-Catacomb horizon/final Northcaucasian Culture*,* MAMS-30831 3702±23 BP (cal. BC 2136-2040, cal. BC 2194-2027), hb; **MAMS-51673: 3620±16 BP (cal. BC 2020-1950, cal. BC 2033-1900), hb**.
- **KMM030,** BZNK-1090/1, Komsomolec 1-Marfa, grave 45. *Context information*: Single inhumation in a grave-pit without contours in supine position with the head in the north-east. Brown organic pads with chalk powder were documented, as well as a vessel and a large ceramic fragment. *Cultural affiliation & dating:* Northcaucasian culture, MAMS-51674: 4191±23 BP (cal. BC 2880-2704, cal. BC 2887-2674), hb.
- **KMM032,** BZNK-1092/1, Komsomolec 1-Marfa, grave 47. *Context information*: Single inhumation supine position and the head in the north in a rectangular pit with wooden box. Below the individual brown organic matter was documented, as well as spots of ochre at the legs*, but* no inventory. *Cultural affiliation & dating:* Northcaucasian culture, MAMS-51675: 4205±23 BP (cal. BC 2886-2707, cal. BC 2893-2680), hb.
- **KMM033,** BZNK-1093/1, Komsomolec 1-Marfa, grave 48. *Context information*: Single inhumation in left-side crouched position with the head in the east in a stepped square pit with wood and reed remains on the walls. The lower leg bones were disarticulated. Remains of a brown organic matter were found beneath the individual, as well as pieces of ochre at the left arm, but no inventory. *Cultural affiliation & dating:* Northcaucasian culture, MAMS-51676: 4173±26 BP (cal. BC 2876-2699, cal. BC 2882-2635), hb.

- **KMM036,** BZNK-1096/1, Komsomolec 1-Marfa, grave 54. *Context information*: Single inhumation in left-side crouched position in a stepped rectangular pit with a wooden box or coffin. The upper part of the body was disarticulated. Below the individual remains of organic bedding and ochre at the feet were documented, but no inventory. *Cultural affiliation & dating:* Northcaucasian culture, MAMS-51677: 4208±21 BP (cal. BC 2886-2708, cal. BC 2893-2699), hb.
- **KMM038,** BZNK-1009/1, Komsomolec 1-Marfa, grave 58. *Context information*: Single inhumation of a child in supine position with the head in the north-west in a stepped pit. A brown organic layer on the ground and beneath the individual a light grey organic layer was documented, as well as ochre spots at the feet, but no inventory. *Cultural affiliation & dating:* Northcaucasian culture, MAMS-51678: 4202±24 BP (cal. BC 2884-2706, cal. BC 2892-2678), hb.

2.3.15 Konstantinovskiy 4

Country: Russian Federation

Region: Zheleznovodsk district, Stavropol region

Coordinates: 44.051815°, 43.132786°

Excavation details: Excavation 'Nasledie' 2013, Stavropol, licence №2012-1507, 2013-164 (Ya. B. Berezin).

Site information: Group of burial mounds on the Konstantinov plateau east of the town Inosemcevo. Several mounds have been excavated during rescue excavation. In mound 4 a destroyed Maykop(?) grave was found, followed by several graves of the Middle Bronze Age. The archaeological material is unpublished.

- **KST001,** BZNK-1044/1, kurgan 4, grave 17. *Context information*: Single inhumation in north-south orientation with a wooden covering in a rectangular grave-pit. The grave was robbed and destroyed in antiquity. but a ceramic vessel was documented. *Cultural affiliation & dating:* Maykop/Late Steppe Eneolithic steppe, MAMS-42351: 5012±19 BP (cal. BC 3907-3714, cal. BC 3939-3709), hb.

2.3.16 Konstantinovskiy 6

Country: Russian Federation

Region: Zheleznovodsk district, Stavropol region

Coordinates: 44.05445°, 43.13455°

Excavation details: Excavation 'Nasledie' 2013, Stavropol, licence №2012-1507, 2013-164 (Ya. B. Berezin).

Site information: Barrow cemetery comprising six mounds, situated in the inner part of the so-called Konstantinovsky Plateau to the east of the town Inosemcevo, which borders the valley of the Podkumok river in the south and east. The site was used as an orchard and is thus heavily destroyed. The mounds were excavated in 2013 by Y. B. Berezin[81]. Mound 6, which had a height of c. 1.75 m and a diameter of c. 40 m prior to excavation, comprised 28 interments. Graves 26 and 28 date to the Eneolithic and were covered by a low mound of ca. 1 m height during the first building phase. Two more mound shells and a stone circle were added later.

- **KT6001,** BZNK-1012/1, kurgan 6, grave 28. *Context information*: Single inhumation in an oval pit. The individual was placed in supine position with bent up knees, with the head in the southeast. The entire skeleton was stained in varying degrees of red ochre. A separate stain of red paint was found in the south-eastern corner of the pit and at the feet of the buried person. The inventory included a long flint blade, a piece of ceramic and shaped pieces of red paint, which disintegrated before salvage. *Cultural affiliation & dating:* Steppe Eneolithic, MAMS-42352: 5383±20 BP (cal. BC 4323-4177, cal. BC 4331-4072), hb.

2.3.17 Krasnogvardeyskoe

Country: Russian Federation

Region: Krasnogvardeysk district, Stavropol region

Coordinates: 45.82500°, 41.58397°

Excavation details: Excavation 'Archaeological laboratory of the Stavropol State Pedagogical University', 1995 (Y. B. Berezin)

Site information: The excavated barrow was a single standing mound situated 3 km to the east of the village Krasnogvardeyskoe on the left terrace above the floodplain, 1 km south of the river Egorlyk. The Egorlyk is a steppe river, linking the Stavropol highlands and the tributaries of the Manych river system. The barrow occupied the top of a small natural promontory that intruded into the floodplain. The mound had possibly never been ploughed and its surface was overgrown. The mound was 2.97 m high and 60 m in diameter. Excavations revealed 23 burials. Three mound shells were identified, providing information on the stages of the construction. Four of the find complexes were associated with the Yamnaya culture (graves 14-15, 20-21), one is attributed to the North Caucasian culture (grave 1), two to the Novotitarovskaya culture (graves 8 and 11), and seven to the Catacomb culture (graves 2, 9-10, 13, 16, 18, 23). Three more graves cannot be dated more precisely than to the Bronze Age (graves 4, 12, 22) and another three are ascribed to the Late Bronze Age, probably associated with the Srubnaya culture (graves 3, 5,17). The final three burials date to the Sarmatian period (graves 3, 5, 17). We sampled and screened six individuals from this site (Supplementary Table 2), of which all were suitable for 1240k SNP capture, after which the following five yielded sufficient ancient human DNA for further analyses (Supplementary Table 1):

- **KNK001,** BZNK-1016/1, Krasnogvardeyskoe, kurgan 1, grave 8. *Context information*: Single inhumation in a stepped pit that is imitating a wagon(?), heavily disturbed by bulldozers. The skeleton was found in the right-side, slightly flexed position, with the head in the northwest. The feet were coloured red with ochre. Below and around the skeleton a layer of dark brown organic matter was documented, and beneath it a larger layer of red-brown organic matter and a thick layer of charcoal around the legs. The floor beneath was burnt and the pit floor covered with lime. The walls were lined with wood, and along the south-eastern wall remains of organic matter with woven pattern (mat?) were documented. A silver earring, a bronze dagger, and bracelet of 50 bronze beads were found. *Cultural affiliation & dating:* Novotitorovskaya/Yamnaya, MAMS-45959: 4158±26 BP (cal. BC 2871-2674, cal. BC 2878-2631), hb.
- **KNK002,** BZNK-1017/1, Krasnogvardeyskoe, kurgan 1, grave 14/1. *Context information*: Multiple inhumations in a stepped pit. Individuals 1 and 2 are adults in supine position with bent legs and heads in the east and feet strewn with ochre. The position of skeleton 3 (a child) is not recognizable, but the head is also in the east. The walls of the pit were covered with wooden beams and lime, the floor plastered with lime layer, and with brown wooden matter on top of it. A vessel, three pebbles, a piece of ochre, and a hematite disc were found as burial gifts. *Cultural affiliation & dating:* Yamnaya, MAMS-45960: 4327±26 BP (cal. BC 3008-2897, cal. BC 3012-2894), hb. *Comment*: Paleoproteomic analysis proved the consumption of dairy products from sheep milk[31].
- **KNK005,** BZNK-1020/1, Krasnogvardeyskoe, kurgan 1, grave 15. *Context information*: Foundation grave of the mound included a single inhumation in a stepped pit with a skeleton in supine position with bent legs and the head in the east. Charcoal was found on the chest and in the pelvis area. The walls were lined with a white organic matter (reed?) and the floor of the grave-pit was covered with light brown organic matter. The inventory included a stone pestle, a vessel and pottery fragments, a piece of ochre, a leather case with six flint tools, three bone tools, a bronze awl, and a stone tool. *Cultural affiliation & dating:* Yamnaya, MAMS-45961: 4173±26 BP (cal. BC 2876-2698, cal. BC 2883-2636), hb, *Comment*: First-degree relative (brother) of individual KNK007.
- **KNK006,** BZNK-1021/1, Krasnogvardeyskoe, kurgan 1, grave 19. *Context information*: Single inhumation in right-side, flexed position with head in the east in a pit, of which the shape was not recognizable. Yellowish organic material (remains of the grave cover?) was found directly on top of the bones, and lime on and around the hand bones. The inventory included two bronze plates, a bronze dagger, a piece of sulphur and a flint flake. *Cultural affiliation & dating:* Belozerka, MAMS-45962: 3075±29 BP (cal. BC 1400-1294, cal. BC 1420-1261), hb.
- **KNK007,** BZNK-1022/1, Krasnogvardeyskoe, kurgan 1, grave 20. *Context information*: Foundation grave of the mound included a single inhumation in a stepped earth pit with remains

of four wheels (a wooden wagon not preserved or a wagon representative) deposited in the corners of the pit. The skeleton was found in supine position with bent legs and the head in the east. Two ochre patches were recorded below the feet, a layer of soot in the torso area, and yellowish organic matter under the bones (remains of clothes?). Dark brown organic matter (felt?) was found above and below the individual on the entire floor of the pit. The upper part of the pit was probably covered with reeds and lime plaster that left a white organic matter with a fibrous structure. The lower part and possibly the floor of the pit were covered by wooden planks. Except of the four wheels (i.e., a wagon), rim sherds, four bone objects, a bone scraper, a small animal bone, a spoon-like bone object, pieces of ochre, a bronze dagger, a stone pestle and an undetermined stone object represent the burial inventory. *Cultural affiliation & dating:* Yamnaya, MAMS-45963: 4212±26 BP (cal. BC 2890-2707, cal. BC 2899-2696), hb, *Comment:* First-degree relative (brother) of individual KNK005. Paleoproteomic analysis proved the consumption of dairy products from sheep and goat milk[31].

2.3.18 Kurganniy 1

Country: Russian Federation

Region: Kirov district, Stavropol region

Coordinates: 44.02265°, 43.88901°

Excavation details: Excavation 'Nasledie', Stavropol 2017, licence №2017-1775 (K. B. Kolesnichenko)

Site information: The mound Kurganniy 1 was located 1 km north of the northern outskirts of the village Kurgan on the left river terrace of the Kura River. The region is today situated in a steppe environment. The mound was 3.2 m high and 44 m in diameter. The kurgan embankment was constructed in two stages on top of grave 17 (mound shell 1) and grave 16 (mound shell 2). A total of 17 burials and two ritual complexes were opened inside the mound. Grave 17 is most likely from the Eneolithic period, while others can be attributed to the steppe variant of the Maykop culture (graves 10, 13-15). Grave 16 dates to the final Middle Bronze Age and one burial to the Sarmatian period, whereas the cultural affiliation of the remaining interments is unclear. The archaeological materials are still unpublished.

All seven individuals sampled yielded sufficient ancient human DNA for further analyses:

- **KUG001,** BZNK-1104/1, kurgan 1, grave 10. *Context information*: Poorly preserved single inhumation in supine position, with the head in the west. The burial pit was difficult to outline, but the floor of the grave was covered with bright red ochre. At either side of the shoulders a ceramic vessel, at the right leg a whetstone, as well as a bronze awl and two flints were documented. *Cultural affiliation & dating:* Steppe Maykop, late, MAMS-51693: 4679±24 BP (cal. BC 3513-3376, cal. BC 3521-3372), hb.
- **KUG002,** BZNK-1105/1, kurgan 1, grave 13. *Context information*: Single inhumation in a small pit with rounded corners. The individual was placed in a sitting position, later collapsed, with the head in the north-west. No inventory was found. *Cultural affiliation & dating:* Steppe

Maykop, late, MAMS-51694: 4580±20 BP (cal. BC 3369-3345, cal. BC 3489-3136), hb. *Comment*: Paleoproteomic analysis proved the consumption of dairy products from sheep milk[31].

- **KUG003,** BZNK-1106/1, kurgan 1, grave 14, individual 1. *Context information*: Double inhumation of two children in a small pit with rounded corners. The individuals were placed in crouched position, individual 1 (western individual) on the left side, individual 2 (eastern individual) on the right side, both heads in the east. A hemispherical, ochre-coloured ceramic bowl was found between the heads of the two individuals. *Cultural affiliation & dating:* Steppe Maykop, late, MAMS-51695: 4546±24 BP (cal. BC 3362-3128, cal. BC 3367-3104), hb. *Comment:* Both individuals are not closely biological related (i.e., first- or second-degree).
- **KUG004,** BZNK-1107/1, Kurganniy 1, kurgan 1, grave 14, individual 2. *Context information*: See above. *Cultural affiliation & dating:* Steppe Maykop, late, MAMS-51696: 4531±16 BP (cal. BC 3354-3124, cal. BC 3361-3105), hb.
- **KUG005,** BZNK-1108/1, kurgan 1, grave 15. *Context information*: Remains of a single inhumation in a large roundish pit or catacomb. Only the skull, vertebrae and small bones were preserved, but not in anatomically correct position. Organic matter was documented below some of the bones, and a ceramic vessel was the sole burial inventory. *Cultural affiliation & dating:* Steppe Maykop, late, MAMS-51696: 4531±16 BP (cal. BC 3354-3124, cal. BC 3361-3105), hb.
- **KUG006,** BZNK-1109/1, kurgan 1, grave 16. *Context information*: Single inhumation in a deep, rectangular burial pit. The poorly preserved skeleton was positioned in a left-side, crouched position with the head in the east. Below the skeleton a multi-layered organic bedding on top of chalk and ochre layers was documented. Four elongated pits in the floor of the grave, which indicate the placement of a wooden wagon that is not preserved on top of the deceased. Two ceramic vessels, a bronze hook, a bronze dagger or knife, a bronze awl and a bronze arrow-head represent the grave inventory. *Cultural affiliation & dating:* Catacomb, late, MAMS-51698: 4041±16 BP (cal. BC 2578-2495, cal. BC 2622-2476), hb.
- **KUG007,** BZNK-1110/1, Kurganniy 1, kurgan 1, grave 17. *Context information*: Single inhumation in supine position with the head in the east in an elongated, oval burial pit. Plenty of red ochre powder was found below the skeleton on the floor of the grave and the bones of the skeleton were as well painted with ochre, in particular the skull, the thorax and the legs. No inventory. *Cultural affiliation & dating:* Steppe Eneolithic, MAMS-51699: 5375±22 BP (cal. BC 4321-4173, cal. BC 4329-4062), hb. *Comment*: Paleoproteomic analysis proved the consumption of dairy products from sheep and goat milk[31].

2.3.19 Magas-Puteprovod

Country: Russian Federation

Region: Nasyr-Kortskiy district, Ingushetia

Coordinates: 43.17661°, 44.80217°

Excavation details: Excavation 'Ingushetia Expedition, Institute Archaeology RAS', Moscow, 1996 (K. V. Voronin)

Site information: The site was excavated during construction work for the newly built capital Ingush Magas in 1996[82]. The monument is a flat, not quite round mound of stones with one grave (grave 7) in the centre and seven other burials arranged in a circle around it. The graves are simple pits filled with stones in which the individuals were buried in a crouched position.

One individual from grave 4 provided genome-wide data.

- **PUT001,** BZNK-0956/2, Magas-Puteprovod, Grave 4. *Context information*: Single inhumation of a 4-5-year-old child in left-side crouched position and head in the east in an earth pit with stone lined walls. A thin layer of grey-brown organic matter directly above the skeleton was found in the entire pit. Burial gifts included turquoise glass and fragmented stone beads. *Cultural affiliation & dating:* Arkhon-Kudakhurt group/post-Catacomb horizon, MAMS-42354: 3643±19 BP (cal. BC 2034-1960, cal. BC 2129-1943), hb.

2.3.20 Marinskaya 5

Country: Russian Federation

Region: Kirov district, Stavropol region

Coordinates: 43.92500°, 43.53000°

Excavation details: Excavation 'Lomonosov Moscow State University', 'Institute of Archaeology RAS', 'Nasledie' 2009, licence № 2009-745 (A. R. Kantorovich), 2009-762 (V. E. Maslov)

Site information:

The huge, single-standing burial mound of Marinskaya 5, situated 2.3 km southwest of the mound group Marinskaya 3 was excavated in 2009 by a team of the Lomonosov Moscow State University, the Institute of Archaeology RAS and the local heritage organisation 'Nasledie'. The kurgan is situated on the high terrace of the river Kura. The mound was slightly oval with a diameter of 34-40 m and 4.3 m high. At a distance of 10-16 m, a ditch of 1.5 m depth surrounded the mound, which was detected on aerial images and excavated later. In the central part of the mound, the excavations uncovered three mound-shells or construction phases dating to the Early Bronze Age Maykop epoch and one that was constructed during the Middle Bronze Age. The first constructive feature in the mound was the above-ground burial vault of grave 33, surrounded by an oval fencing built from river pebbles. The first shell, an earth and stone construction, was built directly on top of this construction. Two following early Maykop burials 32 and 34 were entrenched in the centre. The second mound-shell was associated with late Maykop burials 12 and 16 and was added later. The third mound-shell was constructed after the interment of late Maykop grave 25. These mound-shells were complex earthen constructions, and each was covered by a stone shell. All burials except grave 25 were situated on top of each other in the centre

of the mound. Three burials were dendro-chronologically dated, all other inhumations were radiocarbon dated. The Maykop stratigraphy has recently been published in Russian[83]. In several graves, among them late Maykop grave 25 and North Caucasus graves 19, 23, 30/30a, paired cattle skulls were found. Thus, Marinskaya 5 is one of the earliest sites where the use of cattle as draught animals is documented in a chronological sequence[41]. After a hiatus of 600 years, 18 graves (no. 3, 10, 13, 15, 17-24, 26-29, 30A, and 31) dating to the North Caucasian cultural formation were added, including another mound shell. Two graves (no. 4 and 14) date to the Late Bronze Age/post-Catacomb epoch based on burial practice and radiocarbon dates, and two nearly destroyed burials (no. 8 and 11) perhaps also date to the Bronze Age. One catacomb grave, also dating to the local Middle Bronze Age, intersected the ring of the North Caucasian graves and destroyed an earlier interment (no. 30/30A). During the Late Iron Age five additional graves were added in the centre of the mound. which can be attributed to the Sarmatian epoch. Grave 1 is associated with the Early Iron Age of the late 8th to early 7th century BC and the others (no. 2, 5, 6, 9) date to the early Sarmatian epoch, i.e., the 3rd to 1st century BC. The site is central to a series of multidisciplinary studies, including stable isotope analysis, physical anthropology and archaeogenetic investigations[29,31,32].

We sampled and screened a total of 24 individuals, including 12 newly sampled individuals from this site (Supplementary Table 2), of which eight were not suitable for 1240k SNP capture. The remaining four and two repeated attempts from a previous batch were used 1240k SNP capture, after which three yielded sufficient ancient human DNA for further analyses (Supplementary Table 1):

- **MK5001[29],** BZNK-0065/3, kurgan 1, grave 12. *Context information*: Partly disturbed inhumation in an oval pit framed by wooden beams, grave bottom formed by a pebble layer of the ceiling of grave 32 covered with organics and white sand. The skeleton was partly destroyed but placed in a flexed position on the left side, with the head in the southwest. On top and below the skeleton red ochre was documented. The grave was possibly robbed before the Middle Bronze Age graves above were dug into the mound. Burial gifts included a bronze dagger, two flint arrowheads, a ceramic vessel, and sheep bones. In the fill of grave 10, small fragments of wood, ceramics, two faience (?) beads, animal bones and cattle teeth with bronze oxide remains originate probably from this burial. *Cultural affiliation & dating:* Maykop, late, dendro-dated to 3279±25 BC.
- **MK5003,** BZNK-0247/3, kurgan 1, grave 23. Single inhumation in a deep, rectangular stepped burial pit inside a wooden chamber surrounded by a layer of river pebbles, chamber roofing with four massive timbers and wooden planks. Timber log constructions were documented on the sides of the chamber and vertical posts in the corners. The skeleton was found in a prone position laid diagonally into the chamber, with the head in the southwest and red ochre at the feet. No grave goods were found inside the grave, but two poorly preserved cattle skulls were placed at the eastern end of the burial pit outside the wooden chamber, and the grave fill

contained a ceramic fragment and a bronze pin. *Cultural affiliation & dating:* North Caucasian culture, dendro-dated to 2671±13 BC.

- **MK5004,** BZNK-0073/4, kurgan 1, grave 25. *Context information*: Disturbed inhumation in a square chamber surrounded by wooden posts and covered by wooden planks. Outside the wooden chamber, a stone filling and a stone packing on top of the grave and the related mound embankment was documented. The skeleton was placed in a flexed position on organic material. Bones had been dislocated. Below the skeleton, an area of 2 m in diameter was plastered with clay. The grave inventory included two bronze daggers, one golden ear-ring, one ceramic vessel, one bone arrowhead, and two cattle skulls with nose rings and a badly preserved wooden object (yoke?, fixing pole?) outside the chamber. *Cultural affiliation & dating:* Maykop, late, MAMS-13001/MA-110554: 4498±30 BP (cal. BC 3336-3104, cal. BC 3352-3094), wood, *Comment:* 4-5th degree related to ESY005. Paleoproteomic analysis proved the consumptions of dairy products from milk of bovids[31].
- **MK5008**[29]**,** BZNK-0066/2, kurgan 1, grave 16. *Context information*: Inhumation burial in a rectangular pit with a ceiling and a bottom covering of organic matting (reed?). The skeleton was placed in a right-side flexed position with the head in the southwest. Red ochre was documented on top of the skeleton, as well as the remains of a wooden object (dish?). *Cultural affiliation & dating:* Maykop, late, MAMS-29810: 4544±25 BP (cal. BC 3363-3125, cal. BC 3369-3103), hb.
- **MK5009**[29]**,** BZNK-0064/2, kurgan 1, grave 10. *Context information*: Inhumation in supine position, with the head in the west-north-west, in a deep rectangular pit with stone cover, wooden burial chamber with ceiling of at least seven planks. White and partly yellow coloured organic matter was documented below the skeleton, red ochre below parts of the skeleton and on the grave floor, and parts of the wooden chamber were burned. The grave inventory included a stone object with traces of ochre and animal bones. In the grave fill objects probably originating from grave 12 that was directly situated below grave 10 were found. *Cultural affiliation & dating:* North Caucasian culture, dendrochronologically dated 2644±13 BC.
- **MK5014,** BZNK-0072/2, kurgan 1, grave 26. *Context information*: Inhumation in supine position with head in the northeast, in a long-oval stepped burial pit without remains of a ceiling. Below and on top of the skeleton organic matter and probably leather remains were documented, but no inventory was found. *Cultural affiliation & dating:* Northcaucasian culture.
- **MK5022,** BZNK-0245/4, kurgan 1, grave 14. *Context information*: Poorly preserved inhumation in left-side, flexed position, with the head in the east, in an oval pit. White organic matter was documented below the skeleton. The burial inventory included a ceramic vessel, bronze and jet beads. *Cultural affiliation & dating:* North Caucasian culture, final, MAMS-30833: 3878±26 BP (cal. BC 2453-2299, cal. BC 2464-2236), hb.

- **MK5025,** BZNK-0112/1, kurgan 1, grave 17. *Context information*: Inhumation in supine position, with the head to the south-south-east, in a deep stepped long-rectangular burial pit with ceiling of wooden planks. Organic matter and red ochre were documented below the skeleton, but no inventory was found. *Cultural affiliation & dating:* North Caucasian culture.

2.3.21 Nalchik graveyard

Country: Russian Federation

Region: Nalchik district, Kabardino-Balkaria

Coordinates: 43.48526°, 43.60708°

Excavation details: Expedition 'State Hermitage' 1923 (M. I. Ermolenko), 1929-1930 (A. P. Kruglov, B. B. Piotrovskiy, G. B. Podgackiy)

Site information: The Nalchik cemetery was located in a former area of garden plots on the grounds where the administration of the Republic of Kabardino-Balkaria and a hospital are located today[84]. The first graves were detected in 1923 and 26 of them were opened by M. N. Ermolenko. In 1929-1930 the central area of the cemetery was excavated over a total area of 258 m^2. The site was reported to be a flat hill of ca. 30 m diameter and 0.67 m in height that was part of a group of larger kurgans that covered the wider area. A re-examination of the documentation, however, cannot confirm the presence of a burial mound and suggests a natural elevation[22].

In the central area excavated 1929-1930 a total of 121 burials were documented beside many remains of obviously destroyed graves. They are published with a catalogue and a scheme locating the graves. These formed two main clusters with a nearly empty area in-between, while graves were distributed relatively evenly and separated from each other at the periphery. Some of them were covered by stones, but burial pits were not documented. The individuals were laid rather chaotically in flexed positions on the right (n = 30) or left (n = 27) side and oriented in east-west, north-south or south-west-northeast direction. In 12 cases the deceased were placed in a supine position with bent legs, and in five cases in prone position, also with bent legs. The positioning was probably gender-based. Some graves were double or collective interments. Most individuals were adults, but also 27 children were recorded. The majority of the bones had traces of red ochre, and the intensity of coloration is considered as a chronological marker. The Nalchik graves are generally dated to the Eneolithic period, characterised by informative burial gifts such as stone bracelets, flint blades, copper rings, or boar-tusk pendants, yet pottery was absent. S. N. Korenevskiy suggested based on the variation of ochre coloration and other arguments that some of the graves might date to the MBA. He considered only 51 of the graves excavated in 1929-1930, i.e., those with intensive colour traits, to be from the Eneolithic period. Among them are graves 42 and 86 studied here. Their skeletal material is kept in the State Hermitage in Saint-Petersburg.

- **NCK001,** BZNK-1224/1, Nalchik, grave 42. *Context information*: Inhumation of a group of five individuals that were placed on top of each other. Individual 42 is placed in a crouched

position on the right side. Its skull is situated below the legs of individual 44 and the scattered bones of individual 41. Red ochre was present on all skeletons and the grave goods included several beads and a stone bracelet. *Cultural affiliation & dating:* Eneolithic, MAMS-51679: 5616±28 BP (cal. BC 4489-4370, cal. BC 4531-4359), hb. *Comment*: Related in the 3rd or 4th degree to NCK002.

- **NCK002,** BZNK-1225/1, Nalchik, grave 86. *Context information*: One of the few burials with inventory at Nalchik cemetery is female burial 86. It is a single individual in a crouched position on the right side. The skull was trepanned and the individual was adorned with a rich assemblage of small white cylindrical beads, two stone bracelets and a copper wire ring at the temple. Thirty complete and 17 fragments of perforated deer and bovid incisors, cylindrical tubes and two beads made from fragments of stone bracelets, as well as many small white beads were also found. In addition, the fragment of a rim from a pottery vessel, a fragment of flint blade and a black stone are reported, as was the red ochre on the skeleton. Cultural affiliation & dating: Eneolithic, MAMS-51680: 6776±23 BP (cal. BC 5712-5643, cal. BC 5719-5632), hb, low collagen; MAMS-67249: 6375±17 BP (cal. BC 5365-5321, cal. BC 5465-5232); **GrA-24442[85]: 5910±45 BP (cal. BC 4836-4721, cal. BC 4930-4686), hb**. The radiocarbon dates for the complex are systematically too old. While the date MAMS-51680 suffered from low collagen values, the collagen values of the MAMS-67249 and GrA-24442 dates were sufficient. Since we now know that this individual is related in the 3rd or 4th generation to the one from grave 42, it should not be more than 84-112 years older[86]. The second date is within the average of comparable graves with similar inventories. We therefore suspect a strong reservoir effect in this case, comparable to the difference between charcoal and human bone in grave 37 of the Progress 2 site[29] and suggest a position in the mid-5th millennium BC. We use the GrA-24442 date as the best proxy. *Comment*: Related in the 3rd or 4th degree to NCK001.

2.3.22 Nevinnomyskiy 3

Country: Russian Federation
Region: Nevinomysk district, Stavropol region
Coordinates: 44.73486°, 41.93875°
Excavation details: Expedition 'Research Institute of Archaeology and Ancient History of the North Caucasus', Stavropol 2012, licence №2012-1145 (S. V. Myachin)
Site information: In 2012 several barrows were excavated in the mound field of Nevinnomyskiy 3[87], a group of at least 25 mounds situated in a line running from southwest to northeast along the ridge of a promontory on the right bank of the Kuban River. The region is characterised by a steppe environment, yet is situated near the foothills of the Stavropol highlands. All mounds were rather low and heavily ploughed over. Mound 6 is located in the central part of the group beside the largest mound 1. It had a stone circle of 15 m diameter and contained 14 interments, most of which are attributed to the Bronze

Age. Mound 6 was built by communities associated with the Yamnaya culture, which started the mound with three graves. Five burials belonged to other, later cultures and cultural and chronological groups of the Middle Bronze Age. The largest group of five burials were associated with the post-Catacomb or Lola culture on the basis of similar characteristics. Among them is grave 6 with equipment of a blacksmith and a highly unusual anthropological status. One burial dates to the Iron Age. The second individual in this study was buried in mound 7, which was also low, surrounded by a ditch and revealed the remains of at least two mound shells as well as a stone circle. A complex sequence of burials in pits and catacombs were found in this mound. Grave 23 was the central founding grave and is attributed to the Maykop epoch. It was surrounded by a stone cromlech of about 13 metres in diameter and was covered by an earthen embankment. One individual was published in our previous study[29], but is included here for context:

- **NV3001_NV3002[29],** BZNK-0312/1 & BZNK-1023/1, kurgan 6, grave 5. *Context information*: A single inhumation in left-side, heavily crouched position, with the head in the east-south-east, in a burial pit, which is in fact a reduced catacomb outside of the earlier stone circle. An organic bedding was documented below the inhumation. Anthropological examination revealed several healed fractures of the hands and ribs, as well as severe and chronic inflammations in parts of the upper body and the skull. The musculature of the upper body was well-developed, indicating additional stress on his upper extremities. More pronounced muscle development in the left arm, as well as a fracture in the left hand, characterise the individual as left-handed. The bones of the right leg also show the consequences of a traumatic incident which led to an ossification of the femur after inflammation. The individual probably suffered from myositis, an inflammation of the skeletal musculature, which can be caused by infections, instabilities of the immune system, or a toxic matter. This injury caused severe restrictions to his mobility. In his hands an elevated level of trace elements such as arsenic, lead and mercury were found, which is probably associated with his casting activities. The burial equipment included a metal casting set consisting of a crucible and a tuyere made from clay, a whetstone and a cattle scapula. *Cultural affiliation & dating:* Lola/post-Catacomb horizon, MAMS-29812: 3631±22 BP (cal. BC 2027-1956, cal. BC 2127-1924), hb. *Comment*: NV3002 was processed as a separate individual but was found to be identical to NV3001[29].The genome-wide data of both was merged subsequently. Paleoproteomic analysis proved the consumption of dairy products from sheep and goat milk[31].
- **NV3003,** BZNK-1003/1, kurgan 7, grave 23. *Context information*: Single inhumation in a crouched position, with the head in the north, in the remains of a rectangular burial pit. Dark-brown organic matter was documented below the skeleton, chalk at the knees and under the skull, and red ochre at the pelvis, but no inventory was found. *Cultural affiliation & dating:* Maykop/Late Steppe Eneolithic steppe, MAMS-42353: 4953±20 BP (cal. BC 3765-3655, cal.

BC 3776-3652), hb. *Comment:* 4th-5th-degree related to AY2001. Paleoproteomic analysis proved the consumption of dairy products from sheep and goat milk[31].

2.3.23 Poselenie Kravchenko XII

Country: Russian Federation

Region: Severskiy district, Krasnodar region

Coordinates: 44.92426°, 38.49687°

Excavation details: Excavation 'Rosarcheolog', Rostov-na-Donu 2017, license (T. V. Cybriy).

Site information: The settlement Kravchenko XII is located 460 m south-southwest of the Kravchenko farm in the Abinsky district, Krasnodar region, on the watershed between the Zybza and Bugai rivers. An early mediaeval settlement, Kravchenko 12, is the main archaeological monument in this area. It is located on the left bank terrace of the Kuban River, where it is intersected by a gully. A canal runs along the eastern edge of the monument and a ravine along the western edge. On the right side of the ravine a low but pronounced embankment is found with two burial mounds next to the settlement (kurgans Kravchenko XIII and Kravchenko XVI). It is possible that the embankment is the edge of an ancient flood terrace of the Bugay River, its dike or the bank of an old oxbow. The area slopes towards the north and is ploughed over considerably. The size of the monument is about 410 m in the north-south direction and 215 m in the west-east direction and has a total area of about 60,800 m^2. Traces of settlements as well as graves from different epochs have been discovered in this area. Initially, it was assumed that the burials dated to the Maykop culture, but radiocarbon dates showed that of the seven dated complexes, two are from the end of the 3rd millennium BC (Grave 15-16, Post-Catacomb horizon, Kuban group), one complex (Grave 12) dates to the middle of the 2nd millennium BC (Late Bronze Age), one grave (Grave 4) dates to the second half of the 2nd millennium BC and three are Early Middle Ages, i.e. fall into the second third of the 1st millennium AD. For this study, only the Bronze Age individuals were included. The archaeological material is unpublished.

We sampled and screened 13 individuals from this site (Supplementary Table 2), of which seven were suitable for 1240k SNP capture, after which the following three Bronze Age individuals yielded sufficient ancient human DNA for further analyses (Supplementary Table 1):

- **KVO008,** BZNK-1055/3, Grave 15. *Context information*: No information to the complex available. Cultural affiliation & dating: Kuban group/post-Catacomb horizon, MAMS-45965: 3736±27 BP (cal. BC 2199-2048, cal. BC 2272-2036), hb.
- **KVO009,** BZNK-1056/2, Grave 16. *Context information*: No information to the complex available. Cultural affiliation & dating: Kuban group/post-Catacomb horizon, MAMS-45966: 3759±26 BP (cal. BC 2273-2136, cal. BC 2285-2042), hb.
- **KVO013,** BZNK-1061/1, Grave 1. *Context information*: No information to the complex available. Cultural affiliation & dating: Srubnaya, MAMS-42368: 3303±19 BP (cal. BC 1611-1534, cal. BC 1617-1517).

2.3.24 Sengeleevskiy 7

Country: Russian Federation

Region: Sengeleevskiy district, Stavropol region

Coordinates: 45.98990°, 41.72158°

Excavation details: Excavation 'Research Institute of Archaeology and Ancient History of the North Caucasus', Stavropol, 2006, licence №2006-1143 (V. A. Kuznecov).

Site information: The Sengileevsky 7 cemetery is located in the Shpakovsky District of Stavropol Krai, about 15 km west of the urban area of Stavropol. The burial ground consists of nine barrows, located in a compact group on the edge of promontory situated on the first high terrace of the left bank of the Egorlyk River and 3.2 km south of the Sengileev Lake. The area is part of the western slopes of the Stavropol upland. Barrow 2 was the south-easternmost mound in the group. It was almost completely ploughed down, as a result of which its embankment merged with the embankment of the neighbouring barrow 1. The preserved height of mound 2 was 0.5 m and its diameter was about 30 m. It was constructed in two stages. A total of 14 burials were excavated in the mound, most of which belong to the early Bronze Age, including the Yamnaya and Catacomb cultures. The archaeological material is unpublished, but the studied individual is discussed in a publication on early trepanations[88].

- **SNG001,** BZNK-1011/1, Sengeleevskiy 7, Kurgan 2, Grave 12. *Context information*: Founder grave of the mound. Single inhumation in rectangular pit with rounded corners. The individual laid on its back in a crouched position, with the head in the southeast. Burial gifts included a ceramic vessel. *Cultural affiliation & dating:* Steppe Eneolithic, Poz-63971: 5375±30 BP (cal. BC 4326-4169, cal. BC 4332-4059). *Comment*: The skull of the individual was trepanated at the sagittal line.

2.3.25 Sharakhalsun 3

Country: Russian Federation

Region: Apanasenkov district, Stavropol region

Coordinates: 45.72094°, 43.98044°

Excavation details: Excavation 'Nasledie', Stavropol 2001, licence №2001-791 (A. V. Yakovlev)

Site information: The mound group of Sharakhalsun 3 is one of several lines of burial mounds that run in a slightly north turned to west-east line, parallel to the Kalaus River and about 10 km west of its entry into the Manych River. Other similar mound groups in parallel lines of mounds are Sharakhalsun 1-2, 4-5. The line can be followed on aerial images for ca. 2 km and is less than 1 km south of the major line of Sharakhalsun 6, which runs over 3-4 km. 12 mounds were documented in 2001, and a total of five mounds with 33 graves were excavated during rescue excavations in 2001. Kurgan 5, from where the individual of this study originates, was 0.45 m in height and had a diameter of 19 m. It was constructed in two phases, with the first mound shell associated with Eneolithic grave 8. The other graves and the

second mound shell are attributed to the Early and Middle Bronze Age. The archaeological material is unpublished.

- **SH3001,** BZNK-1004/1, kurgan 5, grave 8. *Context information*: The Eneolithic burial is from the centre of the mound and was found in a long-oval pit. The skeleton was positioned on the back with bent-up legs and the head in the east, but severely destroyed by animal burrows. Pieces of charcoal were preserved under the fragments of the pelvis. Bronze stamped pendants - one whole and seven fragments - were found in the pit fill above the pelvic bones, and a silex blade was found near the right humerus. *Cultural affiliation & dating:* Steppe Eneolithic, MAMS-42355: 5365±20 BP (cal. BC 4319-4169, cal. BC 4326-4058), hb.

2.3.26 Satanaj grotto (Gubsk rock shelter VII)

Country: Russian Federation

Region: Gubskij district, Karachaevo Cherkessia

Coordinates: 44.32367°, 40.62900°

Excavation details: Excavation V. P. Lyubin, P. U. Autlev, X. A. Amirkhanov 1975-1976

Site information: Satanaj grotto is part of a series of Palaeolithic and younger caves and rock shelters in the gorge of the Gubs valley about 50 km southeast of the city of Maykop. The site is also known as Gubs rock shelter 7. It is situated in the rocky outcrops of the gorge, at an altitude of 721 m, about 100 m above the river and has a sequence of ca. 2.8 m of cultural layers in four horizons. Excavations took place on an irregular basis by different scholars between 1961 and 1976.

The stone industry revealed tools characteristic for the local Late Palaeolithic and Mesolithic. Meanwhile the sequence is considered 'Late Epipaleolithic' from the evident similarities in stone tool assemblages to Epipaleolithic sites in the Near East[8]. Modern soil studies recognized charcoal from Holocene tree species in 0.6 cm depth, whereas in 1 m depth a large collection of animal bones have been observed[89]. This layer is associated with the Allerød. The upper horizon (layers 1 & 2) is dated to 7950±140 BP (LE-49, cal. BC 7306-6480) and 7780±200 BP (LE-4982, cal. BC 7247-6231), respectively, while the lower horizon (layers 3 & 4) is dated to 11140±100 BP (SPb-132, cal. BC 11511-11165) and 11200±130 BP (SPb-131, cal. BC 11396-10876), respectively. The studied human bones stem from the second layer.

- **SJG001,** Cultural layer 2. *Context information*: The human bones were found in layer 2 in 1963 (femur) and 1975 (skull, bones of the upper body). This assemblage was not in anatomic order, but with traces of ochre noted. Possible burial gifts in the form of a shell bead and 11 flint tips have been found nearby, which indicate a destroyed burial. *Cultural affiliation & dating:* Epipaleolithic (Mesolithic?), MAMS-37912: 7296±23 BP (cal. BC 6219-6086, cal. BC 6223-6080), hb.

2.3.27 Shushuk 50

Country: Russian Federation

Region: Maykop district, Adygea

Coordinates: 44.275349°, 40.325382°

Excavation details: Excavation Caucasus expedition of the Oriental Museum Moscow 2016-2019, licences 2016-2063; 2019-1824 (V. R. Erlikh)

Site information: The archaeological sites of Shushuk are located on the territory of a gypsum quarry, 1 km southeast of the Svyato-Mikhailovsky monastery located on the outskirts of the settlement Pobeda. Several settlement and dolmen cemeteries have been identified 1.3 km east. The Shushuk dolmen burial ground was investigated by A. D. Rezepkin. The locations are situated on the watershed of the Tolmach and Fars rivers to the east and the Shushuk River to the west. This area is characterised by small hills on gypsum outcrops and karst sinkholes. In total, an area of about 73 hectares is registered as an archaeological monument with 154 identified archaeological objects. Among them are settlement features and dolmen graves.

The partly destroyed dolmen Shushuk 50 is in the eastern part of the archaeological complex[90,91]. At the time of exploration in 2015, the site was overgrown by forest. At the time of excavation, the forest had been cleared and most of the site was covered with overburden from the quarry. Clearing on several levels showed that it represents a megalithic structure that was built on top of a natural hillock.

The contours of the tomb had a rectangular shape of 4.3 × 3.3 m and a northwest-southeast orientation. The tomb was excavated in several layers and only with the third layer the structure of the object became clear, upon noticing that the south-eastern wall of the grave chamber was missing. At this level the first skeletal remains started to appear in disorder. The fourth level revealed fragments of bones and skulls of five to seven individuals in the centre of the grave, as well as a trace of a fire from burning grass or straw. The preservation of bones improved at the 5th level below a thin layer of ash and charcoal. Bones were found without anatomical order, forming an oval cluster stretching along the long axis of the tomb. In the north-western and south-eastern parts of the tomb no bones were found, which suggests that the bones were placed from above through a dismantled central part of the roof, rather than being displaced to the rear wall. The remains of several skulls were found under a fireplace above the north-western half of the bone cluster. The bones of the burials were accompanied by a large number of pottery fragments and bronze items. The burials themselves were placed on a layer of loam with admixture of topsoil, and the slabs of the megalithic construction were buried in the same layer. The bottom of the chamber was made of limestone slabs with longitudinal grooves that formed the foundation of the slabs of the former dolmen with fragments of the side slabs still inserted into these grooves.

We sampled and screened 14 individuals from this site (Supplementary Table 2), of which eight were suitable for 1240k SNP capture, after which five individuals yielded sufficient ancient human DNA for further analyses (Supplementary Table 1):

- **SUS001**, BZNK-1121, grave 1, level 3, individual 1. *Context information*: Megalithic construction, destroyed by a tree growing on it. The contour of the grave construction had a rectangular form and contained remains of 14 individuals. All bones are dislocated and not in anatomical order. The burial contains traces of a fire ritual (traces of burned grass). The inventory consists of fragments of pottery vessels (of at least 14 vessels), stone splitter (n = 4), bronze temporal rings (n = 9), bronze needles (n = 5), fragments of bronze spiral beads (10 beads), and stone beads. *Cultural affiliation & dating:* Dolmen LBA, See above.
- **SUS003,** BZNK-1123/1, grave 1, level 3, individual 3. *Context information*: See above.
- **SUS005,** BZNK-1125/1, grave 1, level 3, individual 5. *Context information*: See above. *Cultural affiliation & dating:* Dolmen LBA: MAMS-55773: 3316±20 BP (cal. BC 1612-1543, cal. BC 1624-1519), hb.
- **SUS006,** BZNK-1126, grave 1, level 4, individual 6. *Context information and dating*: See above. *Cultural affiliation & dating:* Dolmen LBA, layer 4: MAMS-49236: 3084±24 BP (cal. BC 1406-1302, cal. BC 1417-1277), charcoal.
- **SUS008,** BZNK-1128, grave 1, level 5, individual 8. *Context information*: See above.
- **SUS009,** BZNK-1129/1, grave 1, level 5, individual 9. *Context information*: Dolmen LBA, layer 4: MAMS-55774: 3275±20 BP (cal. BC 1599-1504, cal. BC 1612-1501), hb.
- **SUS013,** BZNK-1134/1, grave 1, level 5, individual 13. *Context information*: See above.
- **SUS014,** BZNK-1133, grave 1, level 5, individual 14. *Context information*: See above.

2.3.28 Tipki 1

Country: Russian Federation

Region: Apanasenkovsk district, Stavropol region

Coordinates: 45.740583*°, 44.077167°*

Excavation details: Excavation 'Nasledie', Stavropol 1997, licence № 1997-741 (S. V. Lyakhov)

Site information: Four mounds form the barrow group of Tipki 1, which is located on a gentle slope on the left bank of the Kalaus River, 26.5 km northeast of the village Raguli. The Kalaus River is an important transit route between the Caspian steppe and the North Caucasus piedmont zone. Mound 2 had a diameter of 62 m and a height of 2.86-2.48 m prior to excavations. The construction revealed three building phases, i.e., mound-shells. A ditch surrounded the central part of the mound, and a ritual complex with three concentrations of animal bones was found in the north-eastern part of the mound. A total of 17 tombs were identified in the mound, of which tombs 15 and 16 are the founding graves and belong to the North Caucasus Culture. Graves 4-14 date to different periods of the Bronze Age. Graves 7, 10, 11, and 12 are associated with mound-shell 2, and graves 13 and 14 are associated with mound-shell 3. Graves 8 and 9 also appear to be associated with mound-shell 3. Graves 1 and 17 date to the Early Iron Age, and Grave 3 dates to the Middle Ages.

- **TIP001,** BZNK-1024/1, kurgan 2, grave 4. *Context information*: single inhumation of a male individual in left-side crouched position, with the head in the east, in a rectangular pit with rounded corners. A typical Lola bone pendant or belt hook was found with the human remains. *Cultural affiliation & dating:* Lola/post-Catacomb horizon, GIN-11427: 3640±50 BP (cal. BC 2128-1938, cal. BC 2192-1887), hb. *Comment*: Paleoproteomic analysis proved the consumption of dairy products from sheep and goat milk[31].

2.3.29 Vinogradny 1 (Rostov)

Country: Russian Federation

Region: Ust-Donetsky district, Rostov region

Coordinates: 47.67152°, 40.71792°

Excavation details: Excavation 'Research Institute of Archaeology and Ancient History of the North Caucasus', Stavropol 2017, licence №2017-1159 (S. V. Lyakhov)

Site information: In August 2017 seven of 10 mounds were excavated prior to construction work along the motorway, 3.6 km north-west-west of the village of Krymsky. The area is part of the so-called Donetsk ridge area, represented by the Donetsk denudational and erosional elevation. Its maximum height is 200-300 m with slight slopes and river terraces. The barrow group Vinogradny 1 is located on a watershed formed by the right bank of the Sukhoi Donets River and the right watercourse of the Krymskaya Balka. The necropolis consists of ten barrows located in two groups in the south-eastern corner of the arable field. The north-eastern part are barrows 5-8 lined up in a chain running from northwest to southeast, while the western part with barrows 1-4, 9 and 10 extended in a line with an orientation tilted to the north. Most mounds were heavily ploughed over, only mounds 1, 2 and 7 remained untouched. Mound 1 from where the sampled individual in grave 11 originated was preserved to a dimension of 44-46 m in diameter and had a height of 1.47-3.91 m. It was built with three mound shells. A total of 14 graves were excavated, most of which are attributed to the Middle Bronze Age. Graves 8, 10 and 11 date to the Early Bronze Age, most likely associated with the Yamnaya cultural formation. The archaeological material is unpublished.

One individual sampled yielded sufficient ancient human DNA for further analyses after screening and 1240k SNP capture, while the other did not pass the quality criteria (Supplementary Table 1 and 2).

- **VGD001,** BZNK-1039/1, kurgan 1, grave 11. *Context information*: Single inhumation in a crouched position, with the head in the north-north-east, in an oval pit. Below the skeleton remains of organic matter and spots of ochre were documented, but no inventory was found. *Cultural affiliation & dating:* Yamnaya, MAMS-45970: 4262±27 BP (cal. BC 2905-2882, cal. BC 2918-2781), hb.

2.3.30 Vinsady 4

Country: Russian Federation

Region: Predgorny district, Stavropol region

Coordinates: 44.06013°, 42.96515°

Excavation details: Excavation 'Nasledie', Stavropol 2014, licence №2014-193 (A. V. Lychagin)

Site information: The site of Vinsady 4 is part of a cluster of mounds at the western outskirts of the city of Pyatigorsk. The barrow field consists of at least 12-13 mounds, nine of which were recently excavated but given different site names, including: Vinsadsky 2, Vinsady 4, Vinsady 5, and Skachki 2. More mounds might have been situated beneath a modern industrial park and former military base. Vinsady 4 Mound 2 was situated on the southern slope of a low ridge descending from northwest to southeast in the direction of the Podkumok River valley. The mound was circular in plan, with a diameter of ca. 28 m and a preserved height of ca. 1 m. Inside, the stone cover of the initial mound-shell was found surrounding the Maykop founding grave 11. Fifteen more graves were dug into this mound, most of them associated with the North Caucasus Culture or the Late Bronze Age. The seven North Caucasus burials (Graves 1-5, 8, 12-13) were arranged in two circles surrounding the central grave. Graves 5 and 8 formed the second outer circle. The archaeological material is prepared for publication.

- **VIN001,** BZNK-1007/1, kurgan 2, grave 11. *Context information*: Founding grave of the mound, robbed or intentionally opened to extract parts of the burial material. Large, rectangular grave-pit with a wooden ceiling in the form of perpendicular wooden planks. At the floor dark organic matter with spots of ochre were noted. The chamber was oriented west-east. Two individuals were buried inside, whose skeletal remains were found in different parts along the central axis of the grave mixed with pottery fragments of several Maykop vessels. At the southern wall in its centre a set of a bronze hoe, a bronze shaft-hole axe and bronze chisel was found. Attached to the axe remains of organic mats are still visible. A ritual complex with a large collection of Maykop pottery vessels located at the northwest side just outside the cromlech represents an offering place associated with this burial. *Cultural affiliation & dating:* Maykop, early.
- **VS2001,** BZNK-1008/1, kurgan 2, grave 8. *Context information*: Grave 8 is an unconventional, poorly preserved wooden chamber. The body parts of at least two individuals were disarticulated and placed in the grave. In the centre, a skull was placed on an arm with a shoulder blade, long bones, and hand in anatomical position. Another set of hand bones of similar size were found. Both were placed in an area of red ochre. Somewhat apart from the skull, a third hand was found, belonging to a much larger individual. No burial inventory was found. *Cultural affiliation & dating:* North Caucasian culture, MAMS-45971: 4120±27 BP (cal. BC 2851-2625, cal. BC 2866-2578), hb. *Comment*: Paleoproteomic analysis proved the consumption of dairy products from sheep, goat and cattle milk[31].

2.3.31 Vinsady 5

Country: Russian Federation

Region: Predgorny district, Stavropol region

Coordinates: 44.06000°, 42.96260°

Excavation details: Excavation 'Nasledie', Stavropol 2015, licence №2015-783 (K. B. Kolesnichenko)

Site information: The Vinsady 5 mound group is another part of a larger mound cemetery between the western outskirts of the city Pyatigorsk and the villages Yenisei and Vinsady. The area where the barrows have been built is a flat, gentle slope facing south. Geographically, it is the watershed of two tributaries of the left bank of the Podkumok River and at the same time the edge of the second terrace of this river. The Vinsady 5 mounds are the northern end of an extensive ancient mound group of the Early Bronze Age, including the previously excavated kurgan burial mounds Vinsady 2, Vinsady 4 and Skachki 2. The two Vinsady 5 mounds are separated from each other by 30 metres. The southern mound was labelled No. 1 and the northern one No. 2. The territory of the mounds was subjected to intensive mechanical ploughing for a long time. Excavations took place due to construction work.

Mound 1 was rounded in plan but badly preserved. Its size was approximately 30 m with a remaining height of 0.28-1.38 m. No traces of a ditch were recognizable, but clusters of medium and small limestone fragments on the surface indicated the presence of stone structures in the mound, which after excavation revealed a circular cromlech and a stone mound shell. These constructions are associated with the initial Maykop grave 5. Graves 2-4 date to the Middle Bronze Age, and burial 1 is attributed to the Late Bronze Age. In addition, one extra-burial complex, ritual complex 1, was found. The archaeological material is prepared for publication.

We sampled and screened three individuals from this site (Supplementary Table 2), of which three were suitable for 1240k SNP capture, after which two individuals yielded sufficient ancient human DNA for further analyses (Supplementary Table 1):

- **VS5001,** BZNK-1071/3, kurgan 1, grave 5. *Context information*: Single inhumation in a strongly crouched position on its left side, with the head in the east. No grave-pit could be outlined and no inventory was found. *Cultural affiliation & dating:* Maykop, early, MAMS-51688: 4940±22 BP (cal. BC 3753-3652 1σ; cal. BC 3768-3650), hb.
- **VS5002,** BZNK-1074/2, kurgan 1, grave 1, individual 1. *Context information*: Initial grave of the mound, robbed or intentionally opened to extract parts of the burial material. Large, rectangular grave-pit with wooden frames and a wooden floor on top of which organic matter was recorded. At some spots ochre was noted. The chamber was oriented west-east with a slight tilt to the north. Two individuals were buried inside, whose skeletal remains were found in different parts of the grave not in anatomical order. Individual 1 was documented along the eastern wall of the chamber together with a set of eight Maykop vessels, five of which were aligned along the northern wall and two along the eastern wall. Individual 2 was found in the north-west corner without any objects nearby. A large collection of Maykop pottery sherds in the mound shell indicate an offering place associated with this burial. *Cultural affiliation &*

dating: Lola/post-Catacomb horizon, MAMS-51689: 3676±20 BP (cal. BC 2132-1985 1σ; cal. BC 2139-1977), hb.

- **VS5003,** BZNK-1075/1, kurgan 1, grave 2. *Context information*: Poorly preserved single inhumation in a catacomb. The individual was placed stretched out in supine position, with the head in the south. Organic material and ochre were documented below the human remains. Burial gifts include a bronze pendant and animal bones in the area of the head at the catacombs southern wall. *Cultural affiliation & dating:* Catacomb culture, late, MAMS-51690: hb, no collagen; MAMS-49235: 3820±21 BP (cal. BC 2292-2205 1σ; cal. BC 2395-2149), ab (ovis/capra).

2.3.32 Zimnyaya Stavka 1

Country: Russian Federation

Region: Neftekumsk district, Stavropol region

Coordinates: 44.87001°, 45.30669°

Excavation details: Excavation 'Nasledie', Stavropol 2009, licence №2009-21 (A. V. Lychagin)

Site information: The mounds of Zimnyaya Stavka 1 are located in the eastern part of Stavropol region, where it merges the Caspian lowlands of Kalmykia. The area is today in a semi-desert to desert environment with rather shallow relief. At least 10 mounds are situated on a shallow watershed running west-east just south of the small village Zimnyaya Stavka. Mounds 2 and 6 in the centre are larger in construction, whereas the other mounds are rather small in size. All mounds were rather low, but the embankment of mound 6 was preserved to a height of 1,4 m and with a diameter of ca. 40 m. The mound was built with two shells and a total of 11 graves were documented. A ditch with 16.75 m diameter marked the initial phase associated probably with Middle Bronze Age grave 10. Five of the graves date to the Middle Bronze Age, another five graves (Grave 5, 6, 10-12) are associated with the final Middle Bronze Age, i.e., the post-Catacomb Lola culture. Two more burials are attributed to the Late Medieval. The archaeological material is unpublished. Two of the Lola graves produced genome wide data.

- **ZIM001,** BZNK-1028/1, kurgan 6, grave 11. *Context information*: Single inhumation in a strongly crouched position, with the head in the west, in a small rectangular to oval pit. Behind the individual a set of nine stone tools including whetstones and shaft smoothers, four bone tools and as well as eight flint instruments were placed in a bundle. *Cultural affiliation & dating:* Lola/post-Catacomb horizon, MAMS-45972: 3885±25 BP (cal. BC 2455-2309, cal. BC 2464-2290), hb; **MAMS-11428/MA-103174: 3719±29 BP (cal. BC 2195-2040, cal. BC 2202-2029), ab (ovis/capra)**.
- **ZIM002,** BZNK-1027/1, kurgan 6, grave 10. *Context information*: Single inhumation in left-side crouched position, with the head in the north, in a large and deep rectangular grave-pit. In front of the individual an animal shoulder blade, a stone pestle, stone tools (whetstones?) and a large bone object as well as pottery fragments were placed in a bundle. *Cultural affiliation &*

dating: Lola/post-Catacomb horizon, MAMS-45973: 3703±27 BP (cal. BC 2139-2036, cal. BC 2199-1983), hb.

2.3.33 Zolotaryevka 1

Country: Russian Federation
Region: Ipatovo district, Stavropol region
Coordinates: 45.661255°, 42.555274°
Excavation details: Excavation 'Nasledie', Stavropol 2000, licence №2000-400 (A. A. Kalmykov)
Site information: The Zolotaryevka mound groups represent several distinct clusters of numerous barrows in the vicinity of the village Zolotaryevka at the watershed of the rivers Kalaus and Bolshaya Kugulta, ca. 80 km upstream of Krasnogvardeyskoe. The area is a wide plain, shaped by the small meandering river. All mounds have been heavily ploughed over and have been rather low prior to excavation. The Zolotaryevka 1 group is formed by a line of no less than 79 mounds, running southeast-northwest. The excavated part was situated at its eastern end. The excavations could reveal a chronological shift of the mounds starting from the Maykop period. Mounds 23 and 24, that comprised the individuals in this study, were two small constructions south of the major line. Both held only a single grave. Mounds 26 and 27 are larger constructions. Mound 26 had an oval shape with dimensions of 35 to 25 m and a height of 0.5 m with 16 graves attributed to different Bronze Age cultures. The founding grave is Yamnaya-associated grave 4 of mound shell 1 with a diameter of ca. 6.5-7 m. Two more graves date to the Yamnaya phase, while ten other graves are associated with different phases of Catacomb culture. Mound 27 was 25 m in diameter and only 0.2-0,4 m high. It contained five graves of the Early and Middle Bronze Age. The archaeological material is unpublished.
In addition to previously reported individuals[29], we sampled and screened five new individuals from this site (Supplementary Table 2), of which four were suitable for 1240k SNP capture, all the four individuals yielded sufficient ancient human DNA for further analyses (Supplementary Table 1):

- **ZO1002,** BZNK-1041/1, kurgan 26, grave 4. *Context information*: Grave 4 is the initial grave and located in the centre of the mound. It is a single inhumation in crouched position on its left side, with the head in the west. The grave-pit is round-oval and was covered by stone slabs. The skeleton is covered by red ochre and below the skeleton organic matter was found. The burial equipment is a whetstone and a sulphur lump modelled in a round shape. *Cultural affiliation & dating*: a-typical, probably early or Pre-Yamnaya, MAMS-42369: 5029±22 BP (cal. BC 3936-3780, cal. BC 3947-3713), hb.
- **ZO1004,** BZNK-1043/1, kurgan 27, grave 4. *Context information*: Single inhumation in a roundish pit with a huge, limestone slab set vertically into the grave (stele?) on top of the upper part of the buried individual. The grave is the initial grave in the mound. The individual was placed in a right-side crouched position with the upper body on its back and the head in the southeast. The skeleton was slightly covered with red ochre, below it remains of organic matter

were recorded. A bronze awl is the sole burial gift. *Cultural affiliation & dating:* a-typical, probably early or Pre-Yamnaya, MAMS-45974: 5063±27 BP (cal. BC 3945-3801, cal. BC 3953-3792), hb.

- **ZO1005,** BZNK-1030/1, kurgan 23, grave 1. *Context information*: Single inhumation in a catacomb with a broad, rectangular entrance pit. The individual was buried in a left-side, strongly crouched position, with the head in the east. At the head and torso many beads made from faience, ceramic, antimony and gemstones, at the head a metal perforator(?), animal bones (ovis/capra), a flat thin stone disc, an animal tooth pendant, and at the feet a stone pestle were documented. *Cultural affiliation & dating:* Lola/post-Catacomb horizon, GIN-12407: 3520±40 BP (cal. BC 1918-1770, cal. BC 1953-1700), hb. *Comment*: Paleoproteomic analysis proved the consumption of dairying products from sheep and *bovinae* milk[31].
- **ZO1006,** BZNK-1031/1, kurgan 24, grave 1. *Context information*: Single inhumation in a catacomb with a broad, rectangular entrance shaft. The individual was buried in a left-side, strongly crouched position, with the head in the east. At or in the arms a ceramic vessel, at the hand bones animal bones were documented. In the mound three lower jaws and the extremity bones of adult cattle were found. *Cultural affiliation & dating:* Lola/post-Catacomb horizon, MAMS-42370: 3740±21 BP (cal. BC 2199-2059, cal. BC 2271-2038), hb.

3 Archaeological Site and Sample Information for the non-Caucasus Sites

3.1 Iran

3.1.1 Bozroodpey – Ghal-e Ben

Region: Iran, Mazandaran Province
Coordinates: 36.38000°, 52.56700°
Excavation details: Excavation University of Tehran and University of Babol (H. Fazeli Nashli)
Site information: Ghal-e Ben is a settlement mound that exhibits a sequence from ca. 3300-1500 BC, located at the southern end of Bozroodpey city. Archaeological field work and regional surveys were recently conducted by H. Fazeli Nashli and his team. The ancient mound is partly cut by a modern street and since the beginning of the archaeological activities heavily endangered by building activities. Two small test trenches (5 x 2 m) revealed the stratigraphy of the site. Preliminary radiocarbon dates provide a timeframe of 3300- 3000 BC for the Chalcolithic layers and 2900-1500 BC for the Bronze Age occupation with mudbrick buildings that exhibit plastered clay floors and silo installations. According to surface sampling we can estimate a possible dimension of up to 15 ha for the Bronze Age settlement. The final Bronze Age layers were covered by one metre of oxbow lake deposits indicating that the site's abandonment relates to a profound topographic transformation of the region around 1500 BC.

The studied human bones originate in graves within the site[92]. Two of the four sampled individuals passed the quality criteria for further analysis (Supplementary Table 1 and 2).

- **BOE001,** BZNK-1154/1, Bozroodpey Tepe settlement, Grave Q-31/330-334. *Context information*: Single inhumation of a 45-50-year-old female individual in a simple earth pit. Her flexed body was placed on the left side, with the head in the south. No other grave gift except one small lapis lazuli bead was found. The burial is "sandwiched" between two 'kilns', i.e. fired mud alignments below and above it. *Cultural affiliation & dating*: Iranian Early Bronze Age, Poz-151232: 4080±35 BP (cal. BC 2840-2501, cal. BC 2861-2489), hb.
- **BOE003**, BZNK-1156/1, Bozroodpey Tepe settlement, Grave X35/128. *Context information*: Burial of a senile male individual buried on the right side in a flexed position in a simple pit 2 m above virgin soil. *Cultural affiliation & dating*: Iranian Early Bronze Age, Poz-151233: 4155±35 BP (cal. BC 2871-2671, cal. BC 2881-2623), hb.

4. Summary information on genetic analyses

4.1. The Mesolithic-Neolithic transition

Outgroup-f_3-statistics and various f_4-symmetry-statistics show that SJG001 and EHG individuals from Karelia form a clade with respect to CHG individuals and other *test* populations (Extended Data Fig. 3a, Supplementary Table 6 and 7). The outgroup-f_3-statistics of the form f_3(Mbuti, Test, SJG001/EHG), shows the genetic affinity of SJG001 individual to both Ancient North Eurasian (ANE) and Western European HGs (WHG) ancestries, similar to other EHG individuals as described in previous studies[93–95]. Indeed, we could distally model most of the tested EHG groups as a mixture of Villabruna and ANE (AG3, TTK) ancestry (Supplementary Table 8). The Tutkaul 1 individual was included to increase the representation of the ANE-related ancestry and improve the resolution of the qpAdm models. However, as both these ancestries are much older than the targeted SJG001, we also modelled the SJG001 with other EHG as a single source (Fig. 2c). Individuals from Karelia are a better temporal proxy for SJG001 than Sidelkino_EHG from the Samara region (Supplementary Table 7). The outgroup set for modelling SJG001 is different from the sets used to model subsequent groups. This set was selected to accommodate the distal sources used in the tests (Methods, Supplementary Table 8). In addition, we found evidence of long IBD sharing between SJG001 and UOO033[95] and I0061[96] from Karelia (Supplementary Table 18). However, these results should be viewed with caution as the imputation quality, i.e., genotype probabilities (GP), for individual SJG001 is borderline (0.538) which could result in false positive results.

By contrast, the four Neolithic individuals from Arukhlo (ARO/AO2, Georgia_Neolithic) together with Armenia_Neolithic and Azerbaijan_LN form a cline between CHGs and central Anatolian Neolithic individuals (e.g. Çatalhöyük), which themselves fall on a genetic cline between Anatolian and Levantine ancestry[31–33] (Fig. 2a, Extended Data Fig. 4 and Supplementary Table 5). This genetic affinity to both Anatolian and Levantine Neolithic groups is also apparent in the outgroup f_3-statistics (Extended Data Fig. 3b). Thus, we used qpAdm to model all Neolithic groups from the region using Anatolia_PPN, Levant_PPN and CHG/Iran_N as distal ancestry sources (Fig. 2c, Supplementary Table 8 and Extended Data Fig. 5). We find that Georgia_Neolithic and Armenia_Aknashen_N carry the highest proportion of CHG-like ancestry while Armenia_Masis_Blur_N and Azerbaijan_LN carry more Levant_PPN-related ancestry. Using Iran_N as one of the sources for the Arukhlo individuals was not supported. Indeed, we find that CHG-like ancestry is the preferred source, consistent with results of f_4-statistics (Supplementary Table 7) and more detailed qpAdm involving outgroup rotation (Supplementary Table 8).

Furthermore, using f_4-statistics of the form f_4(Mbuti, *test*; Armenia_N/Azerbaijan_LN, Georgia_Neolithic) we find that Georgia_Neolithic and Armenia_N/Azerbaijan_LN individuals form a clade with respect to *test* populations (Supplementary Table 7). Using qpAdm, all Neolithic South Caucasus individuals can be modelled with each other as a source (from P = 0.2 to P = 0.3), which

suggests a highly similar genetic background (Supplementary Table 8). However, as Georgia_Neolithic are the oldest data so far reported[32,33], we also tested two-way mixture models between Neolithic groups from Anatolia or the Levant as one source and CHG as the second source. We find that Georgia_Neolithic can be modelled as a two-way mixture between Çatalhöyük_N (shown as Anatolia Neolithic in Fig. 2a) or Tell Kurdu and CHG (Fig. 2c, Supplementary Table 5), but not with PPN groups from central Anatolia, Levant and Mesopotamia or Neolithic Northwest Anatolia (i.e., Barcın) (Supplementary Table 8). The proximal qpAdm models for Georgia_Neolithic results in well-fit models with having Cayonu_PPN, NevaliCori_PPNB, Çatalhöyük_N or Tell_Kurdu_EC as the Neolithic source ($0.12 \leq P \leq 0.26$). All feasible models include a Neolithic source that is a mixture of Anatolian-Levantine ancestries. However, exploring the qpAdm output/logile in detail reveals that some of these Çatalhöyük_N as the source in the plot (Fig. 2c). The other models are reported in the Supplementary Table 8.

4.2 Eneolithic (5th millennium BC)

The Steppe_Eneolithic individuals have a genetic affinity to both EHG and CHG, evident from the negative f_3-admixture results (Supplementary Table 9). However, the two-way mixture model with Karelia_EHG is rejected ($P = 1.33\times10^{-6}$) while the one SJG001 is supported ($P = 0.12$) (Supplementary Table 10). The reason for model rejection could be the underestimation of shared genetic drift with Mal'ta 1 (MA1), Levant_PPN and Anatolia_N. To explore whether adding these ancestry components would improve our models, we tested other two- and three-way qpAdm models. First, we replaced CHG with Caucasus_Eneolithic or Azerbaijan_N, which is already mixed with Anatolia_N-like ancestry. In addition, we used Buyukkaya_EC, TellKurdu_EC and Israel_C as a third source to see if it improves model fits. However, these models are still rejected. This suggests that model rejections are unrelated to the presence of this ancestry profile in Steppe_Eneolithic individuals.

The two individuals from Nalchik fall on the intermediate position between *Steppe* and *Caucasus* clusters in PC space (Fig. 2a). The genetic relatedness estimation software calculates background relatedness from the given set of individuals with the assumption that the dataset is made up of mostly unrelated individuals. In cases of small datasets, this value/parameter can be supplied by the user[97]. For this reason, we used a range of background relatedness values (0.2-0.26) as the baseline to calculate genetic relatedness between NCK001 and NCK002. The overall results are consistent with these two individuals being third- or fourth-degree related.

4.3 Late Eneolithic and Early Bronze Age (4th millennium BC)

The Late_Eneolithic individuals genetically resemble the preceding Steppe_Eneolithic and are on the same EHG-CHG cline in PCA. However, the model using Steppe_Eneolithic as a single source lacks statistical support ($P = 0.017$). The reason for the rejection of the model could be attributed to the differential genetic affinity of Steppe_Eneolithic and Late_Eneolithic to CHG in the outgroup set. This

violates one of the theoretical assumptions of qpAdm modelling about experiencing gene flow from an outgroup to either the source or the target[98]. We therefore used more distal sources (CHG mixture with SJG) to model Late_Eneolithic (Supplementary Table 13).

The Maykop_main individuals occupy a similar position to the preceding Caucasus_Eneolithic in PCA, indicating some level of genetic continuity (Fig. 1b and Extended Data Fig. 1). Indeed, f_4-symmetry tests indicate only subtle differences among Maykop_main individuals and between these and the preceding Caucasus_Eneolithic individuals (Supplementary Table 12). For example, Maykop_Novosvobodnaya individuals carry a slightly higher CHG/Iran_N-related ancestry proportion than other Maykop individuals[5], despite the larger geographic distance to the source (Supplementary Table 12). These subtle differences likely resulted in the rejection of the model with Caucasus_Eneolithic as a single source. Hence, we took Caucasus_Eneolithic and Armenia_C as the proximal local substrate, and tested whether a second source with ancestries from the surrounding regions is needed to successfully model Maykop_main individuals. However, all the two-way models were also rejected (Supplementary Table 13). None of the models represents the required genetic ancestry proportions of the target groups. Adding Iran_C groups as a third source, results in improved model fits. Nevertheless, improving the sampling gap from the 5500-4000 BC period in the North and South Caucasus would help the modelling of the Maykop_main individuals.

4.4 From Early to Middle Bronze Age (3rd millennium BC)

As previous studies have shown[96,99,100], the Yamnaya-associated individuals from North Pontic to Samara and Altai mountains form a tight genetic cluster (Fig. 3a). The admixture f_3-statistics confirms a previous observation, the genetic affinity of the Yamnaya groups to distal CHG and EHG ancestries. Compared to individuals from Samara, Yamnaya-associated individuals from the North Caucasus show a higher affinity to Levant_PPN (Z = 3.08) and share less genetic affinity with CHG (Z = -2.13) (Supplementary Table 14). There is some genetic heterogeneity in ancestral composition of Yamnaya-associated individuals which reflects the geography. Western Yamnaya (Ukraine) can be modelled with Eneolithic groups from steppe and CTC/GAC while this model fails for other Yamnaya groups. As mentioned in the main text, addition of WHG-like third source improves the model fits. Moreover, by increasing the number of individuals from Yamnaya, North Caucasus and subsequent Catacomb groups, we discovered subtle genetic variation among them which is also reflected in qpAdm results (Supplementary Table 14 and 15). The two-way model is supported only for North_Caucasus individuals similar to Ukraine_Yamnaya.

Similar genetic heterogeneity is observed among Kura-Araxes individuals. Individuals from Kaps and Talin share higher genetic affinity to Mesopotamia_PPN and Anatolia_N compared to Kura-Araxes from Georgia (Supplementary Table 14). Nevertheless, we grouped the Kura-Araxes individuals from this study with the published ones[29] from our group to increase the resolution of qpAdm models. Most of the Kura Araxes groups can be modelled with Maykop_Novosvobodnaya as a single source

(Supplementary Table 15). However, as some of the f_4-symmetry tests show differential shared drift to the outgroup set between the target (Kura-Araxes) and proposed model (Maykop-Novosvobdnaya), we also tested for a more distal model that was used for Maykop_main individuals and found find statistical support for these models. Thus, we used Maykop_Novosvobdnaya as the single source for the plotting (Fig. 2d).

Two newly reported BA individuals from Iran are positioned similarly in PC space on a Southwest Asian Cline to those from the nearby site Tepe Hissar[101]. We used an alternative (more distal) ancestry modelling, using individuals from Seh Gabi which is on the other end of this cline as a potential source. Since, the f_4-statistcs of the form f_4(Mbuti, *test*; Iran_SehGabi_C, Iran_BA) suggests excess affinity of Iran_BA to Chalcolithic groups from Turkmenistan we used them as the second non-local source (Supplementary Table 14 and 15). Although there is evidence to suggest a local origin (Tepe Hissar), non-local contributions, that is from regions in today's Turkmenistan, cannot be ruled out.

4.5 Final Middle and Late Bronze Age (2nd millennium BC)

The Lola individuals fall close in PC space to Steppe_Maykop individuals and thus represent the westernmost legacy of ANE/WSHG-like ancestry that is visible as red and mint green components in ADMIXTURE (Extended Data Fig. 2). Lola individuals cannot be modelled with Steppe_Maykop as a single source (Lola: P = 2.40×10-16, Lola_2: P = 5.89×10-22), but two-way admixtures of Steppe Maykop and North Caucasus or Steppe_Maykop and Catacomb are supported (Supplementary Table 17). Kazakhstan Kumsay EBA represents a temporally proximal ANE/WSHG source.

We also report one Srubnaya-associated individual KVO013 at the site Pos. Kravchenko XII in the westernmost corner of the North Caucasus steppe zone that falls close to the Corded-Ware/Fatyanovo/Srubnaya cluster in PC space (Fig 3b). The f_4 statistics of the form f_4(Mbuti, Russia_MLBA_Sintashta; Russia_Srubnaya, KVO013) is negative (Z=-3.608). Indeed, this is also confirmed by qpAdm as Russia_Srubnaya can be modelled with Russia_MLBA_Sintashta as a single source but not KVO013 (Supplementary Table 16 and 17). Adding MBA *Steppe* groups from North Caucasus as a second source improves the model fit (Supplementary Table 17).

The positioning of the five Caucasus_LBA_south individuals in the PCA, suggests a certain amount of genetic continuity since the MBA. Thus, we used Armenia_MBA as the local baseline to compute f_4-statistics of the form f_4(Mbuti.DG, test; Armenia_MBA/LBA, Caucasus_LBA_south), which characterises the difference between the preceding Armenia_MBA and Caucasus_LBA_south individuals with respect to ancient test populations (Supplementary Table 16). The resulting f_4-values are mostly consistent with zero, which indicates that Caucasus_LBA_south and Armenia_MBA/LBA individuals are symmetrically related to test groups. Therefore, we could successfully model Caucasus_LBA_south with ARM_Tavshut_Trialeti_MBA as the proximal local source.

4.6 Y-chromosome haplogroup diversity through time

The distribution patterns of Y-chromosome lineages/haplogroups reflect the general genetic clustering observed in the North Caucasus[29]. The extended time transect and increased number of individuals confirms stable Y-chromosome haplogroup composition that remains distinct and mutually exclusive between *Steppe* (mainly R1a/b and Q1a/b) and *Caucasus* (mainly J1/2, G2, H2 and L) clusters (Extended Data Fig. 7b). Y-chromosomal haplogroup diversity is best described over time for the *Steppe*, *Caucasus* and *Intermediate* genetic groups, separately.

The EHG individual SJG001 with the oldest ^{14}C date (~6150 BC) carries R1a2, which although rare, has been found in a Ukraine Mesolithic individual (~8708 BC), and a Khvalynsk individual from Russia (~4611 BC). Then, from around 4300 BCE we observe mostly R1b-V1636 in the Steppe Eneolithic individuals, a haplogroup which was also found in Khvalynsk II[102] in the middle Volga region. However, we also observe two individuals carrying an R1b-M269 derived haplogroup, although we cannot tell which downstream haplogroup they might carry due to coverage. Interestingly, the mixed NCK001 individual of the *Caucasus* cluster also carries the Y-chromosome lineage R1b-V1636. By contrast, Late Eneolithic Steppe outlier individual ZO1002 who carries J2b-L283 is the only case of a South Caucasus-related Y-chromosome lineage in the *Steppe* cluster. Following this, the Steppe Maykop individuals carry Q1b (likely Q1b2), which has been previously found in a Steppe Maykop individual, and one individual carries a lineage of the R1a-M420 major haplogroup. We then observe that the Yamnaya-associated individuals, North Caucasus individuals and Catacomb individuals carry a mix of the Khvalynsk-related R1b-V1636, the Yamnaya-related R1b-Z2103 and in one case a hunter-gatherer-related I2 haplogroup (Catacomb). Interestingly though, while the subsequent Lola individuals also carry R1b-Z2103, we observe reoccurrences of the Steppe Maykop-associated Q1b and in one case, haplogroup N2 (which has also been found in individuals from the Central Steppe, Mongolia and Siberia). Finally, the Steppe Prescythian individuals carry R1b-M269 and R1b-Z2103.

For the *Caucasus*-ancestry related individuals we observe a very different haplogroup distribution over time compared to those of the steppe-related individuals. Around 5500 BCE we observe H2, a haplogroup associated with the spread of agriculture[103]. However, we mostly observe the near-Eastern associated haplogroups J1 and J2 from as early as ~5500 BCE in Neolithic Georgia all the way up to the Highland LBA in ~1700 BCE, also spanning the Caucasus Eneolithic, Maykop, Late Maykop, Kura Araxes and Highland MBA. Interestingly, we also observe several individuals carrying the relatively rare haplogroup L2 in the Maykop and Late Maykop, which has only been found in Maykop[29]- (n=3) and Near Eastern-associated individuals[101,104,105] (n=3) (excluding one such case in Viking Sweden ~987 AD[106]). Finally, it is only in the most recent ^{14}C dated individuals from the Caucasus South, KVO008 and LER005 from the MBA and LBA, respectively, where we find the first occurrence of Y-lineage R1b-Z2103, which is characteristic for, and predominantly found in, EBA steppe pastoralists[107].

For the mixed-ancestry individuals of the *Intermediate* groups we observe a mix of the Y haplogroups observed in the *Steppe*- and *Caucasus*-related individuals. Specifically, we observe R1b-V1636, J1, J2,

R1a and R1b-Z2103 randomly dispersed over the time transect, indicating no apparent pattern of paternally-mediated mixture. One exception is the Steppe Maykop outlier individual carrying the otherwise unobserved Y-haplogroup T1a-FGC1340, however this haplogroup, while rare, is also associated with the Near East[108].

4.7 Mitochondrial haplogroup diversity through time

In contrast to the autosomal and Y haplogroup diversity we find no striking phylogeographic pattern in the mitochondrial haplogroup diversity between the Caucasus and the steppe zone, similar to our previous observation[29]. In general, the mitochondrial haplotype diversity is higher than the Y chromosome haplotype diversity, as estimated by the number of unique haplotypes (Supplementary Table 1). However, it is clear that both uniparentally inherited marker systems cannot be compared directly due to different biological structures and nomenclatures.

The most frequent haplogroups in the *Steppe* are H, T, and U5, whereas K, T, and U4 are the most frequent haplogroups in the *Caucasus* cluster. Comparing the three main autosomal ancestry clouds, haplogroup U8 is the only haplogroup exclusive to the *Caucasus* cluster, whereas haplogroup N1a is exclusive to the *Steppe*. Haplogroups U3 and X are found in the *Caucasus* and the *Intermediate* group, but not in the *Steppe*. By contrast, haplogroup U2 is exclusive to the *Steppe* and the *Intermediate* groups. However, we cannot infer a direction of gene flow from these few observations. In fact, the mitochondrial haplogroup diversity of the mixed individuals of the *Intermediate* groups could be described as a random intersection of both main clusters, and thus rather suggests reciprocal exchange. The large overlap in mitochondrial haplogroups between the *Steppe* and the *Caucasus* cluster is suggesting a small but steady level of female exogamy across larger regions, which also encompassed the two major eco-regions in question. At the same time, the extent of exchange must have been sufficiently low to maintain the autosomal genomic profiles of the main *Steppe* and *Caucasus* meta-populations. When separated into temporal cohorts, we observe that the homogenisation of the mitochondrial haplogroup diversity was already in place during the Late and Final Eneolithic period of the 4th millennium BC (Supplementary Fig. S2), yet acknowledging the small and uneven number of individuals in the preceding periods. The onset of mtDNA homogenisation is consistent with the earliest observation of individuals with mixed *Steppe*- and *Caucasus*-related ancestry. Interestingly, the existing Eneolithic mtDNA diversity is maintained during the Early and Middle Bronze Age, the millennium for which we observe limited gene flow between the *Steppe* and the *Caucasus*.

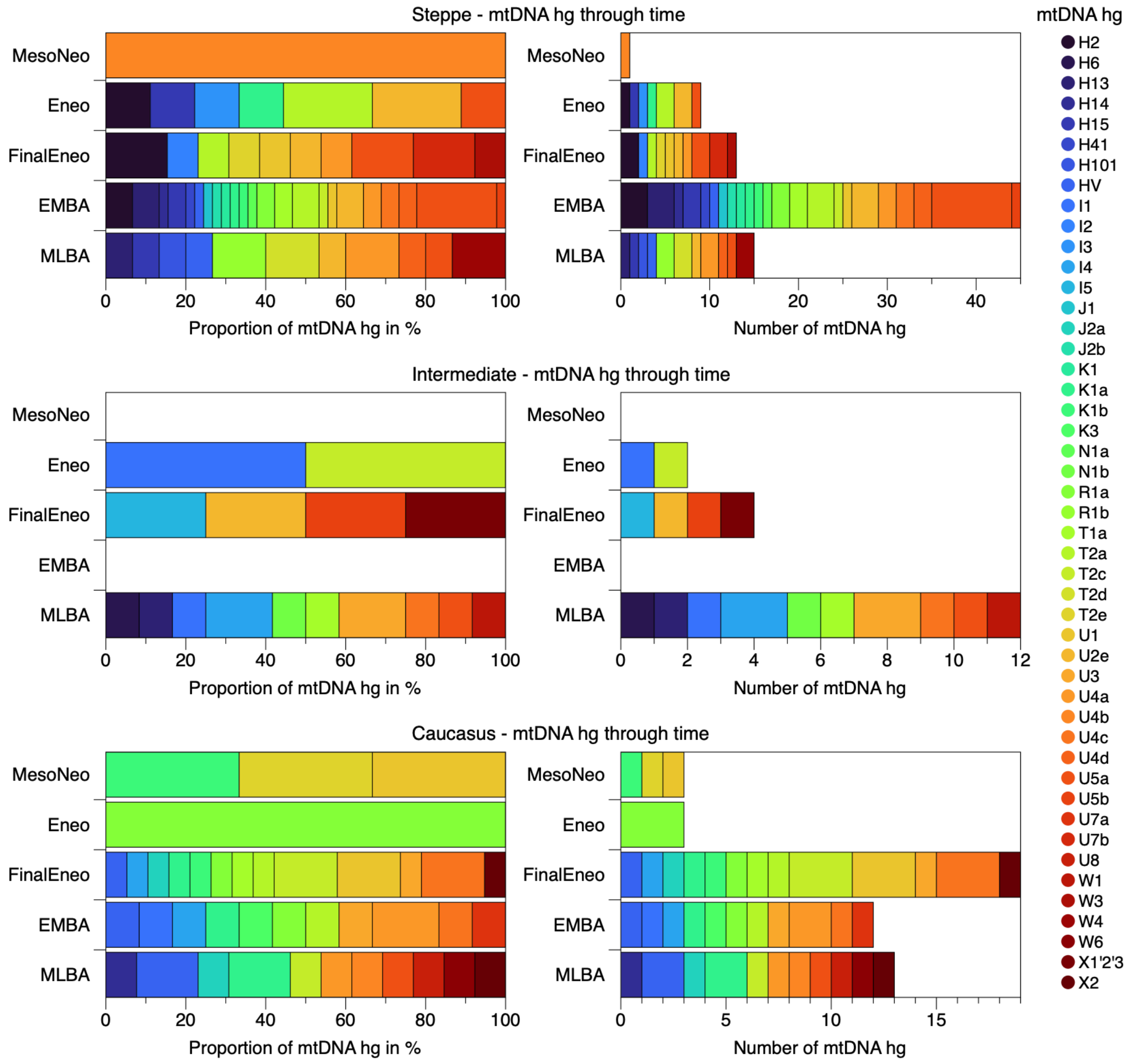

Supplementary Fig. S2. Mitochondrial haplogroup diversity through time. Mitochondrial haplogroups observed in the main autosomal genetic clusters *Steppe*, *Intermediate* and *Caucasus* are separated into the chronological time periods of the 7/6th millennium BC (Mesolithic-Neolithic; MesoNeo), 5th millennium BC (Eneolithic; Eneo), 4th millennium BC (Final Eneolithic; FinalEneo), 3rd millennium BC (Early/Middle Bronze Age; EMBA), and 2nd millennium BC (Middle/Late Bronze Age; MLBA).

5 Supplementary References

1. Bohn, U., Zazanashvili, N. & Nakhutsrishvili, G. The Map of the Natural Vegetation of Europe and its application in the Caucasus Ecoregion. *Bull. Ga. Acad. Sci.* **175**, 112–121 (2007).
2. Dinerstein, E. *et al.* An Ecoregion-Based Approach to Protecting Half the Terrestrial Realm. *Bioscience* **67**, 534–545 (2017).
3. Giemsch, L. & Hansen, S. *The Caucasus: Bridge between the Urban Centres in Mesopotamia and the Pontic Steppes in the 4th and 3th Millennium BC: The Transfer of Knowledge and Technologies between East and West in the Bronze Age: Proceedings of the Caucasus Conference, Frankfurt Am Main*. vol. 34 (Schnell & Steiner, Regensburg, 2021).
4. Barge, O. *et al.* Diffusion of Anatolian and Caucasian obsidian in the Zagros Mountains and the highlands of Iran: Elements of explanation in 'least cost path' models. *Quat. Int.* **467**, 297–322 (2018).
5. Marro, C. & Stöllner, T. *On Salt, Copper and Gold: The Origins of Early Mining and Metallurgy in the Caucasus*. (MOM Éditions, Lyon, 2021).
6. Kohl, P. L. & Trifonov, V. The Prehistory of the Caucasus: Internal Developments and External Interactions. in *The Cambridge World Prehistory 3 Volume Set* (eds. Renfrew, C. & Bahn, P.) 1571–1595 (Cambridge University Press, Cambridge, 2014).
7. Sagona, A. G. *The Archaeology of the Caucasus: From the Earliest Settlements to the Iron Age*. (Cambridge University Press, New York, 2018).
8. Golovanova, L. V. *et al.* The Epipaleolithic of the Caucasus after the Last Glacial Maximum. *Quat. Int.* **337**, 189–224 (2014).
9. Leonova, E. V. Yesho raz o neolite Severnogo Kavkaza. *Rossiskaya Arkheologiya* 43–53 (2019).
10. Rostunov, V. L., Ljachov, S. & Reinhold, S. Tsmi - An open site of the Late Mesolithic and early neolithic in Northern Ossetia (North Caucasus). *Archäologische Mitteilungen aus Iran und Turan* **41**, 47–74 (01 2009).
11. Jones, E. R. *et al.* Upper Palaeolithic genomes reveal deep roots of modern Eurasians. *Nat. Commun.* **6**, 8912 (2015).
12. Amirkhanov, C. A. *Chochskoe Poselenie. Chelovek I Ego Kul'tura v Mezolite I Neolite Gornogo Dagestana*. (Vostochnaya Literatura, Moskva, 1987).
13. Piezonka, H. The World's Oldest Pots: On the Dispersal of the Ceramic Innovation among Eurasian hunter-gatherers since the Late Glacial period. in *Contextualising Ancient Technology* (eds. Klimscha, F., Hansen, S. & Renn, J.) 259–283 (Edition Topoi, Berlin, 2021).
14. Helwing, B. *et al. The Kura Projects: New Research on the Later Prehistory of the Southern Caucasus*. vol. 16 (Dietrich Reimer Verlag, Berlin, 2017).
15. Baudouin, E. *L'architecture de Mésopotamie et du Caucase de la fin du 7e á la fin du 5e millénaire*. vol. 2 (Brepols, Turnhout, 2021).

16. Gorelik, A., Tsybryi, A. V. & Tsybryi, V. V. 'Neolithisation' in the NE Sea of Azov region: one step forward, two steps back? *Documenta Praehistorica* **43**, 139–160 (2016).
17. Mazurkevich, A. N. & Dolbunova, E. V. The oldest pottery in hunter-gatherer communities and models of Neolithisation of Eastern Europe. *Documenta Praehistorica* **42**, 13–66 (2015).
18. Hansen, S. & Helwing, B. Der Beginn der Landwirtschaft im Kaukasus. in *Gold & Wein: Georgiens älteste Schätze (Exhibition Catalogue, Frankfurt)* (eds. Giemsch, L. & Hansen, S.) 27–41 (Nünnerich-Asmus Verlag & Media, Mainz, 2018).
19. Hansen, S. & Bastert-Lamprichs, K. Ein gescheitertes Experiment: Frühe Landwirtschaft im Südkaukasus. *Archäologie Weltweit. Magazin des Deutschen Archäologischen Instituts* 51–55 (2022).
20. Iserlis, M. Sioni und Tsopi - Bauern und Viehzüchter. Ihre Rolle in den kulturellen Transformationen des 5. und 4. Jahrtausends v. Chr. in *Gold & Wein: Georgiens älteste Schätze (Exhibition Catalogue, Frankfurt)* (eds. Giemsch, L. & Hansen, S.) 63–73 (Nünnerich-Asmus Verlag & Media, Mainz am Rhein, 2018).
21. Reinhold, S. *et al.* At the onset of settled pastoralism - Implications of archaeozoological and isotope analyses from Bronze age sites in the North Caucasus. *Quat. Int.* (2023) doi:10.1016/j.quaint.2023.05.008.
22. Korenevskiy, S. N. *Rozhdenie Kurgana: (pogrebalnye Pamyatniki Eneoliticheskogo Vremeni Predkavkazya I Volgo-Donskogo Mezhdurechia)*. (TAUS, Moskva, 2012).
23. Hambleton, E. & Maltby, M. The Animal Bones from Excavations in Meshoko Cave in the Northern Caucasus: Project Report. (2016).
24. Ostashinskiy, S. M., Cherlenok, E. A. & Loskutov, I. G. Novyye dannyye o drevnem zemledelii Severo-Zapadnogo Kavkaza. *Arkheologicheskie vesti* **22**, 35–40 (2016).
25. Anthony, D. W. *et al.* The Eneolithic cemetery at Khvalynsk on the Volga River. *Praehistorische Zeitschrift* **97**, 22–67 (2022).
26. Dellinger, F. *et al.* A 14 C Calibration with AMS from 3500 to 3000 BC, Derived from A New High-Elevation Stone-Pine Tree-Ring Chronology. *Radiocarbon* **46**, 969–978 (2004).
27. Korenevskiy, S. N. & Kalmykov, A. A. Maikop burials of barrow 22 of the Aygurskiy-2 cemetery. *Rossiyskaya Arkheologiya* 77–94 (2017).
28. Shishlina, N. *Reconstruction of the Bronze Age of the Caspian Steppes: Life Styles and Life Ways of Pastoral Nomads*. vol. 1876 (Archaeopress, Oxford, 2008).
29. Wang, C.-C. *et al.* Ancient human genome-wide data from a 3000-year interval in the Caucasus corresponds with eco-geographic regions. *Nat. Commun.* **10**, 590 (2019).
30. Korenevskiy, S. N., Berezina, N. Y., Berezin, Y. B. & Gresky, J. Novyye pogrebeniya protoyamnoy kul'tury na Stavropol'e. in *Gory Kavkaza i Mesopotamskaya step' na zare bronzovogo veka. Sbornik k 90-letiyu R.M. Munchayeva* (ed. Amirkhanov, K. A.) 155–178 (Institut arkheologii RAN, Moskva, 2019).

31. Scott, A. *et al.* Emergence and intensification of dairying in the Caucasus and Eurasian steppes. *Nature ecology & evolution* **6**, 813–822 (2022).
32. Knipper, C. *et al.* Diet and subsistence in Bronze Age pastoral communities from the southern Russian steppes and the North Caucasus. *PLoS One* **15**, e0239861 (2020).
33. Hansen, S. Axes and Metal Depositions in the Caucasus from the 5th to the 2nd Millennium BCE. in *The Caucasus* (eds. Giemsch, L. & Hansen, S.) 31–86 (Schnell & Steiner, Regensburg, 2021).
34. Reinhold, S. Transforming the horizon – Early mounds and monumentalised landscapes in the North Caucasus and their social context. in *Constructing kurgans* (eds. Laneri, N., Palumbi, G. & Müller Celka, S.) 21–40 (Arbor Sapientiae editore, Roma (Italia), 2019).
35. Berezin, Y. B. & Kalmykov, A. A. Kurgan u sela Krasnogvardeyskoe Stavropol'skogo kraya. *Materialy po Izucheniyu Istoriko-Kul'turnogo Naslediya Severnogo Kavkaza* **1**, 55–95 (1998).
36. Kaiser, E. *Das dritte Jahrtausend im osteuropäischen Steppenraum: Kulturhistorische Studien zu prähistorischer Subsistenzwirtschaft und Interaktion mit benachbarten Räumen*. (Freie Universität Berlin, Berlin, 2019).
37. Stöllner, T. *et al.* Between Valleys and Mountains. The Dzedzvebi Plateau as an Intermediate Settlement Site of Late Chalcolithic and Early Bronze Age Communities in the Lesser Caucasus. in *Archaeologia Austriaca* vol. 107 65–136 (Verlag der Österreichischen Akademie der Wissenschaften, 2023).
38. Passeini, A., Rova, E. & Boaretto, E. Chronology (and chronologies) of the Kura-Araxes culture in the Southern Caucasus: An integrative approach through Bayesian analysis. *Origini* **XLI**, 81–138 (2018).
39. Greenberg, R. Oxcarts and the Kura-Araxes Migrations. in *Problems of Early Metal Age archaeology of caucasus and Anatolia. Proceedings* (ed. Narimanishvili, G.) 94–101 (Georgian National Museum, Tbilisi, 2014).
40. Batiuk, S. D. The fruits of migration: Understanding the ʻlongue durée' and the socio-economic relations of the Early Transcaucasian Culture. *Journal of Anthropological Archaeology* **32**, 449–477 (2013).
41. Reinhold, S. *et al.* Contextualising Innovation: Cattle Owners and Wagon Drivers in the North Caucasus and Beyond. in *Appropriating innovations* (eds. Stockhammer, P. W. & Maran, J.) 78–97 (Oxbow Books, Oxford; Philadelphia, 2017).
42. Balanovsky, O. *et al.* Parallel evolution of genes and languages in the Caucasus region. *Mol. Biol. Evol.* **28**, 2905–2920 (2011).
43. Yunusbayev, B. *et al.* The Caucasus as an asymmetric semipermeable barrier to ancient human migrations. *Mol. Biol. Evol.* **29**, 359–365 (2012).
44. Mimokhod, R. A. *Lolinskaja Kul'tura: Severo-Zapadnyy Prikaspiy Na Rubezhe Srednego I Pozdnego Periodov Bronzovogo Veka*. (Institut arkheologii RAN, Moskva, 2013).
45. Reinhold, S., Belinskiy, A. B. & Atabiev, B. C. The Late Bronze Age in the North Caucasus high

mountain zone and beyond. in *Der Kaukasus zwischen Osteuropa und Vorderem Orient in der Bronze- und Eisenzeit* (eds. Kašuba, M. T., Reinhold, S. & Piotrovskiĭ, J. J.) vol. 19 405–456 (Dietrich Reimer Verlag, Berlin, 2020).
46. Mimokhod, R. A., Gak, E. I., Khomutova, T. E., Ryabogina, N. E. & Borisov, A. E. Paleoekologiya – kul'tura – metalloproizvodstvo: printsipy i mekhanizmy epokhi v kul'turnom nasledii Vostochnoy Yevropy v sredniye veka - pervyye pozdney bronzy. *Rossiyskaya Arkheologiya* 24–38 (2022).
47. Librado, P. *et al.* The origins and spread of domestic horses from the Western Eurasian steppes. *Nature* **598**, 634–640 (2021).
48. Manning, S. W. *et al.* A new chronological model for the Bronze and Iron Age South Caucasus: radiocarbon results from Project ArAGATS, Armenia. *Antiquity* **92**, 1530–1551 (2018).
49. Smith, A. T. *The Political Machine: Assembling Sovereignty in the Bronze Age Caucasus*. (Princeton University Press, Princeton; Oxford, 2015).
50. Reinhold, S., Korobov, D. S. & Belinskiy, A. B. *Landschaftsarchäologie im Nordkaukasus: Studien zu einer neu entdeckten bronzezeitlichen Kulturlandschaft im Hochgebirge des Nordkaukasus*. vol. 38 (Habelt-Verlag, Bonn, 2017).
51. Vanchugov, V. P. Das Ende der Bronzezeit im nördlichen Schwarzmeergebiet: die Belozerka-Kultur. *Eurasia Antiqua* **2**, 287–309 (1997).
52. *Srubnaja Obshchnost' Vostochnoj Evropy*. (Bashkirskiy gosudarstvennyy pedagogicheskiy universitet, Ufa, 2006).
53. Chataigner, C., Badalyan, R. S. & Arimura, M. The Neolithic of the Caucasus: Chataigner, Christine, Ruben Badalyan, and Makoto Arimura, 'The Neolithic of the Caucasus'. in *The Oxford Handbook of Topics in Archaeology* (Oxford University Press, Oxford, 2014).
54. Burmeister, S. Early Wagons in Eurasia: Disentangling an Enigmatic Innovation. in *Appropriating innovations* (eds. Stockhammer, P. W. & Maran, J.) 69–77 (Oxbow Books, Oxford and Philadelphia, 2017).
55. Shishlina, N., Ankusheva, P., Orfinskaya, O. & Kiseleva, D. Wool Fibers of the Northern Eurasian Bronze Age: The Cultural and Geographical Contexts. in *The Indo-European Puzzle Revisited. Integrating Archaeology, Genetics, and Linguistics* (eds. Kristian, K., Kroonen, G. & Willerslev, E.) 275–281 (2023).
56. Ryabogina, N., Borisov, A., Idrisov, I. & Bakushev, M. Holocene environmental history and populating of mountainous Dagestan (Eastern Caucasus, Russia). *Quat. Int.* **516**, 111–126 (2019).
57. Borisov, A. V. & Mimokhod, R. A. Aridisaciya: formy proyaveniya i vliyanie na naselenie stepnoy zony v bronzom veke. *Rossiyskaya Arkheologiya* 63–75 (2017).
58. Perşoiu, A., Ionita, M. & Weiss, H. Atmospheric blocking induced by the strengthened Siberian High led to drying in west Asia during the 4.2 ka BP event – a hypothesis. *Clim. Past* **15**, 781–

793 (2019).

59. Eisenmann, S. *et al.* Reconciling material cultures in archaeology with genetic data: The nomenclature of clusters emerging from archaeogenomic analysis. *Sci. Rep.* **8**, 13003 (2018).
60. Burmeister, S. Does the concept of genetic ancestry reinforce racism? *TATuP - Zeitschrift für Technikfolgenabschätzung in Theorie und Praxis* **30**, 41–46 (2021).
61. Mathieson, I. & Scally, A. What is ancestry? *PLoS Genet.* **16**, e1008624 (2020).
62. Roberts, B. W. & Vander Linden, M. *Investigating Archaeological Cultures: Material Culture Variability and Transmission*. (Springer, New York; Heidelberg, 2011).
63. Stuiver, M. & Polach, H. A. Discussion reporting of 14C data. *Radiocarbon* **19**, 355–363 (1977).
64. Reimer, P. J. *et al.* The IntCal20 Northern hemisphere radiocarbon age calibration curve (0–55 cal kBP). *Radiocarbon* **62**, 725–757 (2020).
65. Vardanyan, B., Davtyan, R., Manukyan, S. & Saribekyan, M. Lernakert: ein neues archäologisches Projekt in Shirak. in *Archaeology of Armenia in Regional Context* (eds. Avetisyan, P. & Bobokhyan, A.) 189–200 (Institute of Archaeology and Ethnography NAS RA, Yerevan, 2021).
66. Stoellner, T. & Gambaschidze, I. Das prähistorische Siedlungsplateau Dzedzwebi nahe Balitschi in Unterkartli. in *Gold & Wein: Georgiens älteste Schätze (Exhibition Catalogue, Frankfurt)* (eds. Hansen, S. & Giemsch, L.) 150–157 (Nünnerich-Asmus Verlag & Media, Mainz, 2018).
67. Hollund, H. I., Higham, T., Belinskiy, A. B. & Korenevskiy, S. N. Investigation of palaeodiet in the North Caucasus (South Russia) Bronze Age using stable isotope analysis and AMS dating of human and animal bones. *J. Archaeol. Sci.* **37**, 2971–2983 (2010).
68. Sheremetev, A. G. Raskopki poseleniy I kurganov v Krasnodarskom krae. *Arkheologicheskiye Otkritiya* **2017** , 234–235 (2019).
69. Shilov, V. P. *Drevnie Skotovody Kalmykskykh Stepey*. (Gerel, Elista, 2009).
70. Malek, K. & Ochir-Goryaeva, M. Wagengrab 3 der Kurgan-Nekropole Ergeninskij, Teilrepublik Kalmykien, Russische Föderation – ein Vorbericht. in *Gräberlandschaften der Bronzezeit: Internationales Kolloquium zur Bronzezeit, Herne, 15. - 18. Oktober 2008* (eds. Bérenger, D., Bourgois, J., Talon, M. & Wirth, S.) 499–508 (Landschaftsverband Westfalen-Lippe & APRAB, Darmstadt, 2012).
71. Khokhlova, O., Sverchkova, A., Myakshina, T. & Kalmykov, A. A. A geoarchaeological study of the large Early Bronze Age Essentuksky 1 kurgan in Ciscaucasia, Russia. *Geoarchaeology* **37**, 400–417 (2022).
72. Markovin, V. I. Novyy pamyatnik epokhi bronzy v gornoy Chechne (mogil'nik u sel. Gatyn-Kale). in *Drevnosti Checheno-Ingushetii* (ed. Krupnov, E. I.) 48–138 (Nauka, Moscow, 1963).
73. Berezin, Y. B., Karapetian, M. K. & Kartsev, P. P. Osteological materials from Chechnya and Ingushetia in the collections of the MSU Research Institute and Museum of Anthropology. *Moscow University Anthropology Bulletin (Vestnik Moskovskogo Universiteta. Seria XXIII.*

Antropologia) 83–95 (2021).
74. Gadzhiev, M. G. *Iz Istorii Kul'tury Dagestana v Epokhu Bronzy (Mogilnik Ginchi)*. (Dagestanskiy filial AN SSSR, Makhachkala, 1969).
75. Belinskiy, A. B., Kalmykov, A. A., Korenevskiy, S. N. & Härke, H. The Ipatovo kurgan on the North Caucasian Steppe (Russia). *Antiquity* **74**, 773–774 (2000).
76. Korenevskiy, S. N., Belinskiy, A. B. & Kalmykov, A. A. *Bol'shoy Ipatovskiy Kurgan Na Stavropol'e: Kak Archeologicheskij Istochnik Po ėpoche Bronzovogo Veka Na Stepnoj Granice Vostochnoy Evropy I Kavkaza*. (Nauka, Moskva, 2007).
77. Siliézar, A. *et al.* Analysen stabiler Isotope zur Ernährungsrekonstruktion von bronzezeitlichen Individuen aus Kurgan 2, Ipatovo, Russland. *Bulletin der Schweizerischen Gesellschaft für Anthropologie* **24**, 3–17 (2018).
78. Key, F. M. *et al.* Emergence of human-adapted Salmonella enterica is linked to the Neolithization process. *Nature ecology & evolution* **4**, 324–333 (2020).
79. Korenevskiy, S. N. Utochneniye kolonki radiouglerodnykh dat Bol'shogo Ipatovskogo kurgana i osobennostey simvoliki yego konstruktsii. *Kratkiye soobshcheniya Instituta arkheologii.* **236**, 42–49 (2014).
80. Reinhold, S. *Die Spätbronze- Und Frühe Eisenzeit Im Kaukasus: Materielle Kultur, Chronologie Und überregionale Beziehungen*. Vol. 144 (Habelt-Verlag, Bonn, 2007).
81. Korenevskiy, S. N. & Kalmykov, A. A. Eneoliticheskie pogrebeniya kurgana 6 mogil'nika Konstantinovskiy-6. *Stratum Plus* 385–396 (2017).
82. Voronin, K. V. & Malashev, V. Y. *Pogrebal'nye Pamyatniki Epokhi Bronzy I Rannego Zheleznogo Veka Rasvninnoy Zony Respubliki Ingushetiya*. (Institut arkheologii RAN, Moskva, 2006).
83. Kantorovich, A. R., Maslov, V. E. & Petrenko, V. G. Pogrebeniya maykopskoj kultury kurgana No. 1 mogilnika Marinskaja 5. *Materialy po Izucheniyu Istoriko-Kulturnogo Nslediya Severnom Kavkaza* **10**, 71–108 (2013).
84. Kruglov, A. P., Piotrovskiy, B. B. & Podgayetskiy, G. V. Mogil'nik v g. Nal'chike. in *Materialy po arkheologii Kabardino-Balkari* 67–146 (Institute arkheologii Akademiya nauk SSSR, Moskva and Leningrad, 1941).
85. Shishlina, N. *Reconstruction of the Bronze Age of the Caspian Steppes: Life Styles and Life Ways of Pastoral Nomads*. vol. 1876 (Archaeopress, Oxford, 2008).
86. Fenner, J. N. Cross-cultural estimation of the human generation interval for use in genetics-based population divergence studies. *Am. J. Phys. Anthropol.* **128**, 415–423 (2005).
87. Kalmykov, A. A., Berezina, N., Dobrovol'skaya, M. V., Gresky, J. & Buzhilova, A. P. The burial of a Lola smelter in the Stavropol' region. *Kratkie Soobshcheniya Instituta Arkheologii* **251**, 64–79 (2018).
88. Gresky, J. *et al.* New cases of trepanations from the 5th to 3rd millennia BC in Southern Russia in

the context of previous research: Possible evidence for a ritually motivated tradition of cranial surgery? *Am. J. Phys. Anthropol.* **160**, 665–682 (2016).

89. Aleksandrovskiy, A. L., Aleksandrovskaya, E. I., Belyaeva, E. V. & Golovanova, L. V. Deposits in Caves. Sediments and cultural layers of caves in the Northwest Caucasus. in *Peshchery (Caves): Collection of scientific papers* 35–68 (Institute of Geography RAN, Perm, 2015).
90. Erlikh, V. R. & Gak, E. I. Drevneyshiye olovyannyye bronzy na Severo-Zapadnom Kavkaze. Novyye dannyye. *Oriens* **5**, 36–53 (2020).
91. Erlikh, V. R., Kleshchenko, A. A., Gak, E. I. & Godizov, G. L. The Shushuk archaeological complex – a reference post-dolmen site in the piedmont Northwest Caucasus of the Middle–Late Bronze Ages. *Rossiyskaya Arkheologiya* **1**, 7–23 (2022).
92. Sołtysiak, A., Fazeli Nashli, H., Safari, M. & Thomalsky, J. Human remains from Ghal e-Ben, Iran, 2019. *Bioarchaeology of the Near East* **13**, 97–104 (2019).
93. Raghavan, M. *et al.* Upper Palaeolithic Siberian genome reveals dual ancestry of Native Americans. *Nature* **505**, 87–91 (2014).
94. Fu, Q. *et al.* The genetic history of Ice Age Europe. *Nature* **534**, 200–205 (2016).
95. Posth, C. *et al.* Palaeogenomics of Upper Palaeolithic to Neolithic European hunter-gatherers. *Nature* **615**, 117–126 (2023).
96. Haak, W. *et al.* Massive migration from the steppe was a source for Indo-European languages in Europe. *Nature* **522**, 207–211 (2015).
97. Rohrlach, A. B., Tuke, J., Popli, D. & Haak, W. BREADR: An R Package for the Bayesian Estimation of Genetic Relatedness from Low-coverage Genotype Data. *bioRxiv* 2023.04.17.537144 (2023) doi:10.1101/2023.04.17.537144.
98. Harney, É., Patterson, N., Reich, D. & Wakeley, J. Assessing the performance of qpAdm: a statistical tool for studying population admixture. *Genetics* **217**, iyaa045 (2021).
99. Allentoft, M. E. *et al.* Population genomics of Bronze Age Eurasia. *Nature* **522**, 167–172 (2015).
100. Penske, S. *et al.* Early contact between late farming and pastoralist societies in southeastern Europe. *Nature* **620**, 358–365 (2023).
101. Narasimhan, V. M. *et al.* The formation of human populations in South and Central Asia. *Science* **365**, (2019).
102. Mathieson, I. *et al.* Genome-wide patterns of selection in 230 ancient Eurasians. *Nature* **528**, 499–503 (2015).
103. Rohrlach, A. B. *et al.* Using Y-chromosome capture enrichment to resolve haplogroup H2 shows new evidence for a two-path Neolithic expansion to Western Europe. *Sci. Rep.* **11**, 1–11 (2021).
104. Feldman, M. *et al.* Late Pleistocene human genome suggests a local origin for the first farmers of central Anatolia. *Nat. Commun.* **10**, 1218 (2019).
105. Skourtanioti, E. *et al.* Genomic History of Neolithic to Bronze Age Anatolia, Northern Levant, and Southern Caucasus. *Cell* **181**, 1158–1175.e28 (2020).

106. Margaryan, A. *et al.* Population genomics of the Viking world. *Nature* **585**, 390–396 (2020).
107. Balanovsky, O. *et al.* Genetic differentiation between upland and lowland populations shapes the Y-chromosomal landscape of West Asia. *Hum. Genet.* **136**, 437–450 (2017).
108. Mendez, F. L. *et al.* Increased resolution of Y chromosome haplogroup T defines relationships among populations of the Near East, Europe, and Africa. *Hum. Biol.* **83**, 39–53 (2011).
