# Supplementary material for: The rise and transformation of Bronze Age pastoralists in the Caucasus
Source: Nature. 2024 Oct 30;635(8040):917–25. doi: 10.1038/s41586-024-08113-5 (PMC11602729; doi:10.1038/s41586-024-08113-5)
Supplement: Supplementary file 2 — Reporting Summary [file 41586_2024_8113_MOESM2_ESM.pdf]

nature portfolio

Corresponding author(s): Ayshin Ghalichi
Wolfgang Haak

Last updated by author(s): Sep 19, 2024

Reporting Summary

Nature Portfolio wishes to improve the reproducibility of the work that we publish. This form provides structure for consistency and transparency in reporting. For further information on Nature Portfolio policies, see our Editorial Policies and the Editorial Policy Checklist.

Statistics

For all statistical analyses, confirm that the following items are present in the figure legend, table legend, main text, or Methods section.

n/a	Confirmed	
☐	☒	The exact sample size (*n*) for each experimental group/condition, given as a discrete number and unit of measurement
☐	☒	A statement on whether measurements were taken from distinct samples or whether the same sample was measured repeatedly
☐	☒	The statistical test(s) used AND whether they are one- or two-sided<br>*Only common tests should be described solely by name; describe more complex techniques in the Methods section.*
☒	☐	A description of all covariates tested
☐	☒	A description of any assumptions or corrections, such as tests of normality and adjustment for multiple comparisons
☐	☒	A full description of the statistical parameters including central tendency (e.g. means) or other basic estimates (e.g. regression coefficient) AND variation (e.g. standard deviation) or associated estimates of uncertainty (e.g. confidence intervals)
☐	☒	For null hypothesis testing, the test statistic (e.g. *F*, *t*, *r*) with confidence intervals, effect sizes, degrees of freedom and *P* value noted<br>*Give P values as exact values whenever suitable.*
☒	☐	For Bayesian analysis, information on the choice of priors and Markov chain Monte Carlo settings
☒	☐	For hierarchical and complex designs, identification of the appropriate level for tests and full reporting of outcomes
☒	☐	Estimates of effect sizes (e.g. Cohen's *d*, Pearson's *r*), indicating how they were calculated

Our web collection on statistics for biologists contains articles on many of the points above.

Software and code

Policy information about availability of computer code

Data collection	No specific software was used for sample and data collection. Software used for the processing of raw sequence data and genotype data is listed below.
Data analysis	We used the following freely available software for data analyses and preparation of figures and maps, and provide the corresponding citations in the Material & Methods section: EAGER (v2.4.0), nf core/eager v2.3.2 (https://nf-co.re/eager), (FastQC (v0.11.9), AdapterRemoval (v2.3.2), BWA (v0.7.17), CircularMapper (v1.93.5), MarkDuplicates (v2.26.0), MapDamage (v2.2.1), samtools (v1.3), pileupCaller (v1.5.2), bamUtils (v1.0.13), Sex.DetERRmine tool (v1.1.2), ANGSD (v0.935), contamMix (v1.0 12), Schmutzi (v1.0), ADMIXTOOLS (v7.0.2) (qp3Pop, qpDstats, qpWave and qpAdm [v1520]), admixr (v0.9.1), ADMIXTURE (v1.3.0), EIGENSOFT package (v7.2.1), smartpca (v16000), Haplogrep 2 (v2.4.0), ancIBD (v0.4), hapROH (v1.0), GLIMPSE (v1.0.1), DATES (v4010), OxCal (v4.4.2), READ2 (v2.0), BREADR (v1.0), Geneious (v2019.2.3), Sankeymatic (no versioning), Datagraph (v5.2), R (v4.3.3), R Studio (v2023.12.1+402), and QGIS (v3.36.0). The maps were generated using QGIS and data from Base Relief: © Mapzen, OpenStreetMap (no versioning), and rivers, lakes and border were added using free vector and raster map data from naturalearthdata.com, with final touches in Adobe Illustrator 28.3 and Photoshop 25.5.

For manuscripts utilizing custom algorithms or software that are central to the research but not yet described in published literature, software must be made available to editors and reviewers. We strongly encourage code deposition in a community repository (e.g. GitHub). See the Nature Portfolio guidelines for submitting code & software for further information.

Data

Policy information about availability of data

All manuscripts must include a data availability statement. This statement should provide the following information, where applicable:

- Accession codes, unique identifiers, or web links for publicly available datasets
- A description of any restrictions on data availability
- For clinical datasets or third party data, please ensure that the statement adheres to our policy

Genomic sequence data (fastq and BAM format) will be available at the European Nucleotide Archive under project accession number PRJEB73987. The published genotype data available in compiled and annotated format as Allen Ancient DNA Resource (AADR v44.3) as well as the POSEIDON repository was used for comparative analyses and is available here:
https://reich.hms.harvard.edu/allen-ancient-dna-resource-aadr-downloadable-genotypes-present-day-and-ancient-dna-data.
https://www.poseidon-adna.org/#/
The human mitochondrial revised Cambridge Reference Sequence (NC 012920.1): https://www.ncbi.nlm.nih.gov/nuccore/251831106.
The human reference genome GrCh38 (hg38): https://www.ncbi.nlm.nih.gov/datasets/genome/GCF_000001405.26/
The human reference genome GrCh37 (hg19): https://www.ncbi.nlm.nih.gov/datasets/genome/GCF_000001405.13/
hs37d5 is consistent with GRCh37, and contains the rCRS mitochondrial sequence, Human herpesvirus 4 type 1 and concatenated decoy sequences: https://ftp.1000genomes.ebi.ac.uk/vol1/ftp/technical/reference/phase2_reference_assembly_sequence/hs37d5.fa.gz
The 1000 human genomes reference panel: http://hgdownload.cse.ucsc.edu/gbdb/hg19/1000Genomes/phase3/

Research involving human participants, their data, or biological material

Policy information about studies with human participants or human data. See also policy information about sex, gender (identity/presentation), and sexual orientation and race, ethnicity and racism.

Reporting on sex and gender	not applicable
Reporting on race, ethnicity, or other socially relevant groupings	not applicable
Population characteristics	not applicable
Recruitment	not applicable
Ethics oversight	not applicable

Note that full information on the approval of the study protocol must also be provided in the manuscript.

Field-specific reporting

Please select the one below that is the best fit for your research. If you are not sure, read the appropriate sections before making your selection.

☒ Life sciences ☐ Behavioural & social sciences ☐ Ecological, evolutionary & environmental sciences

For a reference copy of the document with all sections, see nature.com/documents/nr-reporting-summary-flat.pdf

Life sciences study design

All studies must disclose on these points even when the disclosure is negative.

Sample size	We did not determine ancient DNA sample size a priori. Sample sizes for ancient groups/populations depend entirely on availability and preservation of human skeletal remains (and thus ancient DNA molecules) associated to archaeologically described cultures and/or techno-complexes.
Data exclusions	We processed and screened samples from 253 samples from 211 prehistoric individuals, of which 131 were deemed suitable for downstream analyses, following pre-defined data quality and authentication criteria, described in the Methods section. Data from specimens that showed insufficient levels of ancient DNA content or high levels of DNA contamination were excluded from further analyses.
Replication	We study unique entities of past populations and did not use different treatments or variations of data analyses. Experiments are carried out once and replication only occurs partially and randomly, e.g. by generating multiple DNA extracts and/or DNA libraries from the same sample or individuals, or when several samples turn out to belong to the same individual, as can be the case when dealing with commingled remains in collective burials. We recognize that individuals from the same region and time period of the past show similarities, and that their particular ancestry composition does not exist in the same form anymore today. Genome-wide data with hundreds of thousands of SNPs allows for multiple realisations of the sample history.
Randomization	Following model-free approaches such as principal component analysis, prehistoric individuals are grouped by chrono-cultural contexts, i.e.

Randomization	time period (archaeological culture, radiocarbon date), geographic region and genetic similarity. Randomisation is thus not relevant/ applicable to this study.
Blinding	The archaeological and anthropological context of our samples (date, location, material culture etc.) is critical to the interpretation of the data, blinding is not applicable to our study.

Reporting for specific materials, systems and methods

We require information from authors about some types of materials, experimental systems and methods used in many studies. Here, indicate whether each material, system or method listed is relevant to your study. If you are not sure if a list item applies to your research, read the appropriate section before selecting a response.

Materials & experimental systems

n/a	Involved in the study
☒	☐ Antibodies
☒	☐ Eukaryotic cell lines
☐	☒ Palaeontology and archaeology
☒	☐ Animals and other organisms
☒	☐ Clinical data
☒	☐ Dual use research of concern
☒	☐ Plants

Methods

n/a	Involved in the study
☒	☐ ChIP-seq
☒	☐ Flow cytometry
☒	☐ MRI-based neuroimaging

Palaeontology and Archaeology

Specimen provenance	All specimens were collected and analyzed with permissions from the respective local organizations for the handling of the archaeological material, and represented by local curators and collaboration partners who are listed among the co-authors of this study. All relevant organizations and contact persons are listed per site, including excavation licence numbers, are listed under paragraph 2 in the Supplementary Information.
Specimen deposition	Original specimens are stored at the Shirak Armenology Research Center of Armenian Academy of Sciences in Armenia, the Eurasia Department of the German Archaeological Institute in Germany, the Georgian National Museum in Georgia, the Nasledie' Cultural Heritage Stavropol in the Russian Federation, and the Research Institute and Museum of Anthropology of the Lomonosov Moscow State University (RIMA) in the Russian Federation.<br>Specimens will be returned to the respective heritage organization and museums after completion of the joint collaborations. DNA extract and libraries will remain stored at the ancient DNA laboratories of the Max Planck Institute for Evolutionary Anthropology, Jena & Leipizg, Germany.
Dating methods	New AMS 14C dates were obtained from ultra-filtrated collagen of 84 individuals. Collagen extraction and 14C measurements were carried out at the Curt-Engelhorn-Zentrum Archäometrie gGmbH, Mannheim, Germany. All new and published dates from the Caucasus were calibrated on the basis of the IntCal20 database and using OxCal v4.4.2.

☒ Tick this box to confirm that the raw and calibrated dates are available in the paper or in Supplementary Information.

Ethics oversight	No ethics oversight was required. Permission to work on the archaeological samples was granted by the respective excavators, archaeologist, and curators and museum directors of the sites, who are co-authoring the study and who approved and provided guidance on the study protocol. All steps in the analyses followed standard ethical guidelines with regards to respectful handling, documentation, storage, transport, sampling and processing of human skeletal elements. Excavation licence numbers for each site are provided in the Supplementary Information.

Note that full information on the approval of the study protocol must also be provided in the manuscript.

Plants

Seed stocks	not applicable
Novel plant genotypes	not applicable
Authentication	not applicable
