# Supplementary material for: The rise and transformation of Bronze Age pastoralists in the Caucasus
Source: Nature. 2024 Oct 30;635(8040):917–25. doi: 10.1038/s41586-024-08113-5 (PMC11602729; doi:10.1038/s41586-024-08113-5)
Supplement: Supplementary file 3 — A guide to Supplementary Tables 1–20 (tables supplied separately). [file 41586_2024_8113_MOESM3_ESM.pdf]

Supplementary Tables Guide

Supplementary Table 1: General Information
Excel sheet containing general and context information of the individuals newly reported in this study and from Wang et. al. 2019.

Supplementary Table 2: Report of shotgun screening
Excel sheet containing the EAGER report statistics for all individuals after initial shotgun screening.

Supplementary Table 3: Relatedness sites
Excel sheet containing genetic relatedness results for all individuals from each site.

Supplementary Table 4: Relatedness groups
Excel sheet containing genetic relatedness results for all individuals belonging to the same genetic grouping.

Supplementary Table 5: Group Labels
Excel sheet containing context information about the published and modern individuals used in the analysis, as well as additional umbrella group labels for published individuals and groups used in this study.

Supplementary Table 6: Cornerstone f_4-statistics
Excel sheet containing results for f_4-statistics of the form f4(Mbuti, Test; Anatolia N/CHG, X), where *X* denotes CHG/EHG/Iran N and Anatolia N/EHG/Iran N respectively, and *Test* denotes various genetic groups from the *Caucasus* and *Steppe* clusters.

Supplementary Table 7: MesoNeo f_4-statistics
Excel sheet containing results of f_3- and f_4-statistics for genetic groups from the Mesolithic Neolithic transition period. The Z-scores of the threshold $|Z| \geq 2$ and $|Z| \geq 3$ are highlighted in blue and green respectively. The f_4-statistics with low SNP overlap among the tested groups are highlighted in yellow.

Supplementary Table 8: MesoNeo qpAdm
Excel sheet containing results for qpAdm modelling of genetic groups from the Mesolithic Neolithic transition period. Highlighted rows in green represent feasible qpAdm models, red highlights represent infeasible qpAdm models with the reasoning mentioned in the Notes column. Yellow highlighted rows indicate the low SNP overlap among the tested groups in that qpAdm model or an elevated Z-score. The models in bold are shown in the plots.

Supplementary Table 9: Eneolithic 5th f_4-statistics
Excel sheet containing results for f_3- and f_4-statistics for genetic groups from the Eneolithic (5th millennium BC) period.

Supplementary Table 10: Eneolithic 5th qpAdm
Excel sheet containing results for qpAdm modelling of genetic groups from the Eneolithic (5th millennium BC) period.

Supplementary Table 11: DATES
Excel sheet containing estimates for admixture dates for Neolithic, Eneolithic and EBA genetic groups as well as the ancient individuals included in the respective sources.

Supplementary Table 12: Eneolithic 4th f_4-statistics
Excel sheet containing results for f_4-statistics for genetic groups from the Eneolithic (4th millennium BC) period.

Supplementary Table 13: Eneolithic 4th qpAdm
Excel sheet containing results for qpAdm modelling of genetic groups from the Eneolithic (4th millennium BC) period.

Supplementary Table 14: EMBA 3rd f_4-statistics
Excel sheet containing results for f_3- and f_4-statistics for genetic groups from the Early to Middle Bronze Age (3rd millennium BC) period.

Supplementary Table 15: EMBA 3rd qpAdm
Excel sheet containing results for qpAdm modelling of genetic groups from the Early to Middle Bronze Age (3rd millennium BC) period.

Supplementary Table 16: MLBA 2nd f_4-statistics
Excel sheet containing results for f_4-statistics for genetic groups from the Middle to Late Broze Age (2nd millennium BC) period.

Supplementary Table 17: MLBA 2nd qpAdm
Excel sheet containing results for qpAdm modelling of genetic groups from the Middle to Late Broze Age (2nd millennium BC) period.

Supplementary Table 18: IBD analysis
Excel sheet summarizing the results of IBD sharing from ancIBD, reporting both number and total length of IBD tracts longer than 8, 12, 16 and 20 cM, as well as longest IBD block. We also report genotype probabilities (GP) of imputed individuals.

Supplementary Table 19: ROH analysis
Excel sheet containing the results of estimated runs of homozygosity (ROH) for all individuals having more than 400,000 SNPs covered on the 1240k SNP panel (n=X) using the hapROH software.

Supplementary Table 20: PCA populations
Excel sheet containing the list of populations used in the West Eurasian and extended West Eurasian PCAs.
